# Preparation of β-cyclodextrin-based dimers with selectively methylated rims and their use for solubilization of tetracene

**DOI:** 10.3762/bjoc.18.170

**Published:** 2022-11-25

**Authors:** Konstantin Lebedinskiy, Volodymyr Lobaz, Jindřich Jindřich

**Affiliations:** 1 Department of Organic Chemistry, Faculty of Science, Charles University, Hlavova 8, 128 43 Prague, Czech Republic; 2 Institute of Macromolecular Chemistry, Department of Supramolecular Systems and Self-Assembling Processes, Heyrovského nám. 2, 162 06 Prague, Czech Republic

**Keywords:** cyclodextrin, dimer, methylation, solubilization, tetracene

## Abstract

A series of β-cyclodextrin dimers selectively permethylated on the primary or secondary rim with two different types of spacers have been synthesized effectively utilizing conventional and newly developed methods. Their structure analyses by ^1^H NMR and NOESY NMR imply the dependence of molecular symmetry on the type of spacer. The ability of synthesized dimers to increase the solubility of tetracene in DMSO was evaluated and compared to native cyclodextrins and their methylated derivatives. The newly synthesized compounds expressed better effectiveness than other tested supramolecular hosts.

## Introduction

Cyclodextrins (CDs) are cyclic oligomers of glucose that play an important role in supramolecular chemistry [1]. The structure of any CD contains a hydrophobic cavity inside the molecule, while all hydrophilic hydroxy groups are arranged outside the cavity. This feature determines the main practical application of CDs as supramolecular hosts for host–guest interaction. Due to their low cost, low toxicity, and good complexation ability, they are frequently used in pharmaceutical, food, and chemical industries, agriculture, and environmental engineering [1]. They possess many hydroxy groups and are suitable for further chemical transformations that could alter their complexation ability [2]. Every glucose unit in CDs bears three hydroxy groups, i.e., the CD contains three types of hydroxy groups with different chemical reactivity. The difference in the reactivity and combination of synthetic procedures allow obtaining many molecules with selectively substituted positions [3]. Perhaps, the most investigated CD derivatives with selectively substituted rims are partially methylated CDs. Methylation reduces the formation of intramolecular hydrogen bonds, enhancing CDs water solubility, and also extends the hydrophobic cavity, thus improving its binding potential. A substantial increase of binding constant (*K*) for per-6-methylated CD compared to native CD was described, but an order of magnitude decrease of *K* was found for permethylated CD [2]. Also, methylation changes the solubility of CDs in organic solvents, expanding their potential field of application. CDs form the most stable complexes with hydrophobic compounds in polar solvents such as water [4]. The complexation of organic molecules by CDs in nonpolar media has not been widely studied yet, but in several cases, such results have been achieved [5–6]. Another interesting type of CD transformation is a connection of two CD molecules by some spacer via covalent bonds. Many such "dimers" (not exactly the correct name, but widely used) have been reported in the literature [7–17]. The most crucial feature of such dimers is a sufficient increase in binding constants with several potential guests, particularly with ditopic or stick-shaped molecules. So it could be expected that the extension of the hydrophobic cavity by the combined methylation and “dimerization” may improve the binding potential towards such substrates even more.

Linear polycyclic aromatic hydrocarbons (acenes) and their derivatives are good organic semiconductors and show interesting light-absorbance properties. They found their application in material science, where they are used in developing organic photovoltaic prototypes as potential dichroic dyes and organic thin-film transistors. However, due to strong π-stacking interaction, these compounds are not readily soluble, and the solubility decreases with the increased number of aromatic rings in a molecule [18]. Pentacene with five conjugated aromatic rings has been extensively investigated; however, it is quite challenging to investigate the spectral and electrical properties of acenes with a number of rings higher than 6 because of poor solubility and mainly because of their low stability [18–20]. Moreover, a general method for the preparation of long acenes have been recently published [21]. According to the literature [22], the only reasonable solution to overcome the solubility and stability problems is functionalizing these molecules, for example, by inserting some protecting groups. Substituted heptacenes demonstrate remarkable stability and exceptional electric properties.

Nevertheless, studying the properties of unsubstituted acenes is also essential. We guessed that some increase in solubility of acene might be achieved by a supramolecular interaction with a suitable host. Our initial plan was to enhance the solubility of higher acenes by the complexation with CDs. However, linear acenes with more aromatic rings (such as pentacene or heptacene) are, in addition to their poor solubility, also quite unstable, for example, towards oxidation. So, we have chosen tetracene as the object of our research. This molecule is also known as an organic semiconductor and is poorly soluble, but it demonstrates some stability to oxidation, making it easier to work with. We assumed these results might be extended to pentacene and other larger linear acenes if we would achieve some success with it. In this work, we report on the efficient synthesis of new cyclodextrin supramolecular hosts based on selectively methylated β-CD derivatives and their dimers; moreover, we compare their effectiveness in the solubilization of tetracene in DMSO.

## Results and Discussion

### Synthesis of β-cyclodextrin dimers with selectively methylated rims

Selective methylation has been used in CD chemistry for at least 30 years [23–24], and since then, scientists have developed advanced and relatively cheap synthetic procedures [25]. Almost all of these methods are also applicable for the methylation of 6-mono-azido derivatives.

**Scheme 1:**
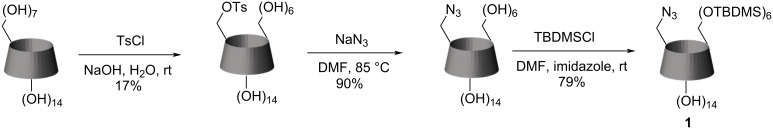
The synthesis of 6^A^-azido-6^A^-deoxy-per-6-*O*-*tert*-butyldimethylsilyl-β-cyclodextrin.

Compound **1** was used as the starting compound to prepare all dimers with partially methylated rims. It contains one azido group in position 6, and all remaining 6-OH are protected with TBDMS groups; it was prepared according to the published procedure (Scheme 1) [26] with some modifications to achieve better results. We started with silylation of 6-azido-β-CD, using imidazole/DMF base/solvent mixture instead of pyridine, which gave higher yields and lower reaction time. Also, we found the recrystallization from the mixture of methanol/acetonitrile to be more suitable for the purification of the compound rather than DCM/acetone [26]. However, we do not recommend recrystallization when the presence of oversilylated compounds in the reaction mixture is too high; here, the column chromatography with CHCl_3_/MeOH elution mixture gives much better yields in a shorter time.

**Scheme 2:**
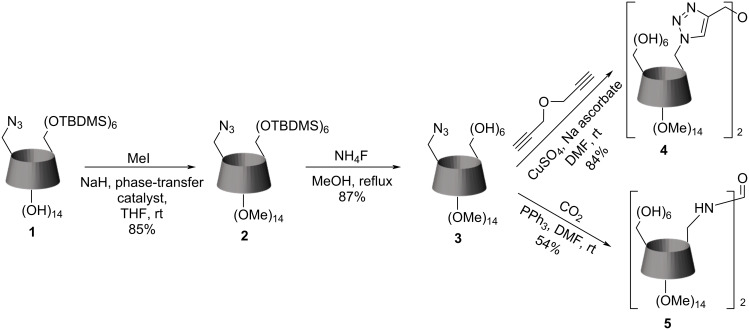
The synthesis of β-cyclodextrin dimers with permethylated secondary rims.

The synthesis of dimers selectively permethylated on the secondary side is shown in Scheme 2. While methylating the secondary rim of the silylated cyclodextrin **1**, we met the obstacle that the reaction mixture contained a non-negligible amount of undermethylated products regardless of the reaction time or the amount of methyl iodide added. In the literature [25], this problem is solved by using methyltriphenylphosphine bromide as a phase-transfer catalyst, and it has also worked in our reaction. Refluxing in NH_4_F methanol solution was chosen to cleave the silyl groups. Both other reagents used for the cleavage in CD chemistry (TBAF and BF_3_^.^Et_2_O) yielded byproducts that unnecessarily complicated the purification.

The CuAAC "click reaction" in CD chemistry is also a well-known approach, allowing coupling reactions of azido-containing CDs with different propargyl-containing compounds, including other CDs, to form a dimer [12]. Usually, such reactions proceed with a Cu(I) catalyst [27]; however, Cu(I) can be generated in situ by the reduction of Cu(II) [12,28] or by the dissolution of metal copper [29]. Moreover, the load of the catalyst varies from catalytical amounts (0.02 equiv) [27] to semi-equivalent [12]. Optimal conditions for a click reaction are a subject to discovery in every case, because temperature, microwave or ultrasonic irradiation, and type of catalyst strongly influence the reaction time and yields.

In the preparation of dimer **4**, the most crucial restriction in coupling two CD units by propargyl ether is the volatility of the latter compound. Thus, we discovered that performing the reaction at room temperature, prolonging the reaction time, and using an equivalent amount of the copper catalyst resulted in the best yields.

Another kind of reaction engaging the azido group in the CD chemistry is the phosphine imide reaction [30–31]. This transformation involves triphenylphosphine and carbon dioxide to convert azide into isocyanate [31], allowing coupling with amines or other nucleophile groups. It is interesting to note that the same conditions lead to a different product in CD chemistry [30]. In the absence of strong nucleophiles such as amines, the CD gives a dimer with a urea bridge instead of providing the isocyanate. The compound **5** was synthesized from **3** according to the standard procedure [30].

**Scheme 3:**
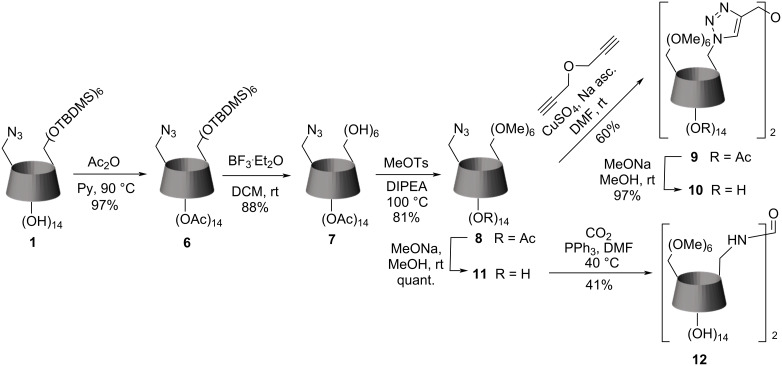
The synthesis of β-cyclodextrin dimers with permethylated primary rims.

The synthesis of dimers selectively permethylated on the primary side is shown in Scheme 3. The method described by Varga [25] was not suitable for the preparation of **11** because of the strong reductive conditions required for the cleavage of benzyl protective groups. Other described procedures [23–24] also have disadvantages, such as the price of some reagents and relatively low yields.

In this work, we describe a method using low-cost reactants, such as methyl tosylate and DIPEA, which provides moderate to high yields and is a reasonable alternative to the existing methods. The acetylation of compound **1** using standard conditions gave the secondary side peracetylated **6** in a high yield. The selective deprotection of silyl groups, yielding compound **7**, was also performed standardly using BF_3_O·Et_2_O. Our new procedure is based on the methylation of this acetyl-protected β-CD **7** using a DIPEA/methyl tosylate mixture without adding any solvent. The DIPEA can be effectively replaced by another relatively weak organic base, such as TEA, TBA, or 2,6-lutidine. The reaction proceeds at 100 °C and is usually finished in 10–12 hours, yielding compound **8**. However, it demands a steady pH control because the basicity of the reaction mixture decreases during the first several hours due to the quaternization of DIPEA. We found that adding small portions of the base over the reaction course allows us to avoid this problem and finish the process successfully. Purifying the reaction mixture requires the removal of excess methyl tosylate, which can be easily done by shaking the reaction mixture with NaI ethanol/water solution in a separation funnel.

The click reaction of compound **8** proceeds at a different rate than **3** under the same conditions. The coupling of **3** can be finished overnight with high yields, whereas the coupling of **8** is much slower and gives a lower conversion. Mourer and co-authors [12] also have reported varying reactivity of 6-azido permethylated CD over 6-azido CD in click reactions, claiming the presence of hydroxy groups on the secondary face reduces the catalytical activity of copper.

Compound **11**, prepared by standard deacetylation of **8**, proved to be not reactive enough to complete the reaction in 24 h under the conditions used to prepare the dimer **5**. The prolongation of the reaction time increased the yield slightly, but it was still too low (17%). To improve the yield, we used lithium iodide and increased the temperature somewhat, giving us a 41% yield after 12 h of the reaction. Testing the same conditions for dimer **5**, we also noticed a speed up in the reaction rate, but a presence of the trimer with an extra CD moiety connected by carbamate group (*M*_r_ ≈ 4000) was spotted, giving us lower yields. We assume the Lewis acid activates isocyanates, affording reactions with alcohols. Since the hydroxy groups on the primary rim of CD express higher nucleophilicity than hydroxy groups on the secondary rim, this type of reaction is not observable with the permethylated primary ring.

### NMR studies of β-cyclodextrin dimers with selectively methylated rims

The important part of this work was proving the structure of the synthesized compounds because we worked with non-symmetrical CDs; moreover, we used protection–deprotection methods for partial methylation, so we could expect a cleavage or even migration of protective groups. Despite this, we have found mass spectrometry and combined NMR analysis to be reliable methods to prove the structure of these compounds. On NMR spectra, we paid particular attention to the H^1^-region, where most of our products have several doublets, which referred to the signals from each hydrogen-1 in every glucose moiety. Sometimes these signals overlap and merge; however, it is still possible to isolate at least one small doublet, whose intensity is 1/7 of the whole H^1^-region. After dimerization, the H^1^-region can be more complex, indicating the further loss of symmetry in the molecules.

The NMR spectra of the dimers possess several features, making them quite different from the spectra of their starting materials. The spectra of the dimers with the urea linker in water contain much fewer distortions in the hydrogen and carbon signals than the spectra of a corresponding azido-compound, so they look more like the symmetrical partially methylated compounds without azido group. This phenomenon could be attributed to the increased molecular symmetry after the dimerization with this type of spacer.

**Figure 1:**
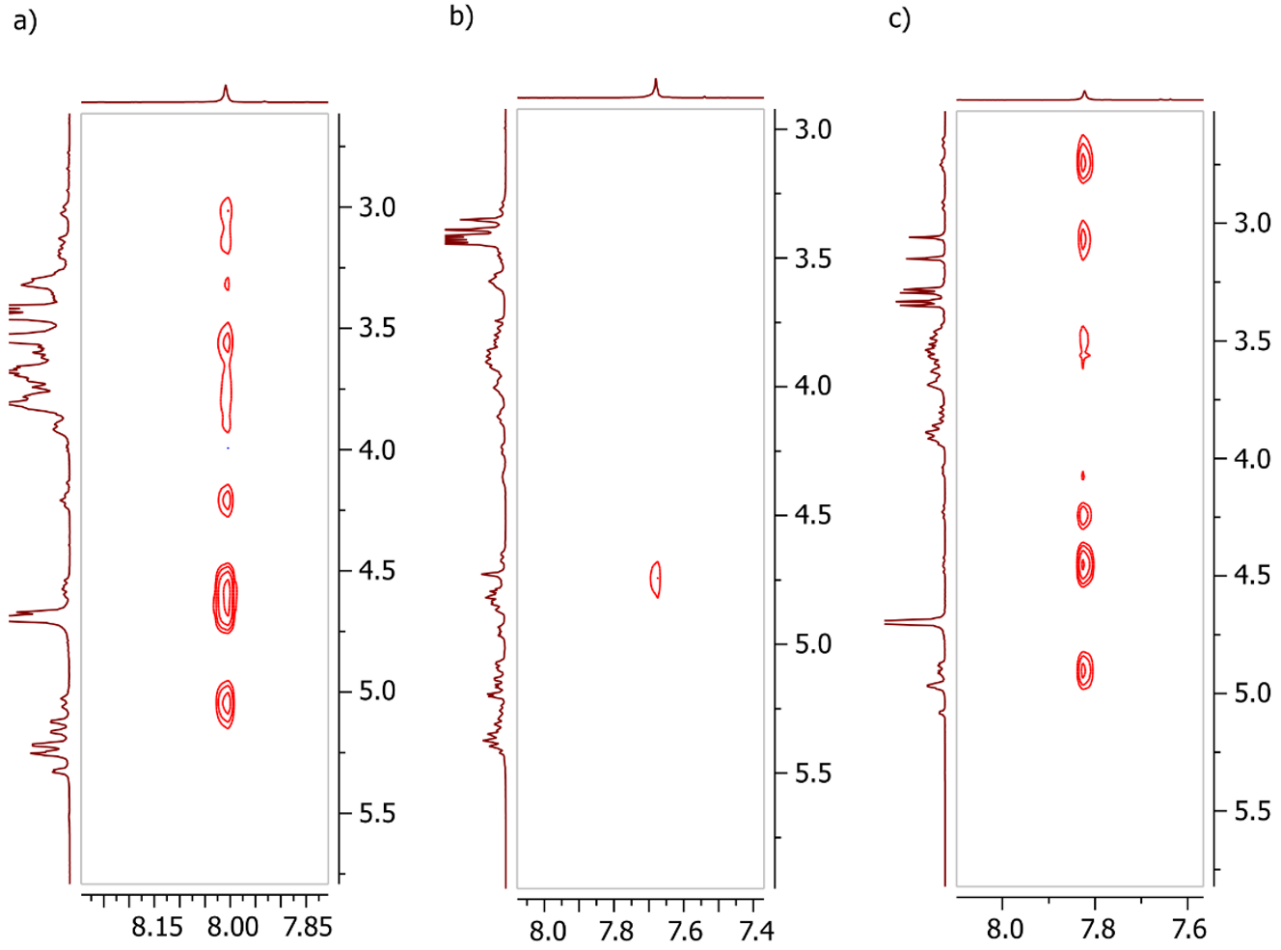
The fragments of ^1^H NOESY NMR spectra of **4** (a), **10** (b), and **9** (c) indicating the interaction between the triazole proton and the internal cavity of the CD.

A methyltriazole ether linker connects the CD moieties in dimers **4**, **9**, and **10**. The aromatic part of the spacer is a less polar substituent than an oxygen atom. It justifies the magnetic anisotropy of the space surrounding the aromatic ring, leading to some specific signals becoming isolated and easily distinguishable. For example, a clearly defined H^6^ signal from the glucose unit A bearing the triazole ring in **10** are at 2.6–3.0 ppm; the 5^A^-hydrogen is also quite distinctive on the spectra (4.17–4.23 ppm). These observations agree with the reported data [13,32] and may help to confirm the structure. The other resonance signals from the CD skeleton are difficult to follow due to total overlapping and the whole asymmetry of the molecule; however, each methyl group in a molecule gives a singlet that is quite prominent on a spectrum, even if it represents only one group. The spectrum of compound **4** contains two sharp peaks at 3.41 (6H) and 3.43 (6H) ppm, which belong to the methyl groups attached to the carbon-3 positions. The other signals (36H) from Me–O–C^3^ are concentrated in two overlapped singlets of different intensities. The single peak at 3.54 ppm represents all signals from Me–O–C^2^, implying that the triazole ring interacts mainly with the hydrogens inside the cavity. Another solid proof of the partial self-inclusion of the triazole into the internal space of CD is the fragment of the NOESY spectrum (Figure 1a), where the interaction of the triazole hydrogen with H–C^6A^, H–C^5A^, and H–C^6^ from the glucose unit next to the unit A is clearly seen indicating the interaction between the triazole proton and the internal cavity of the CD.

**Figure 2:**
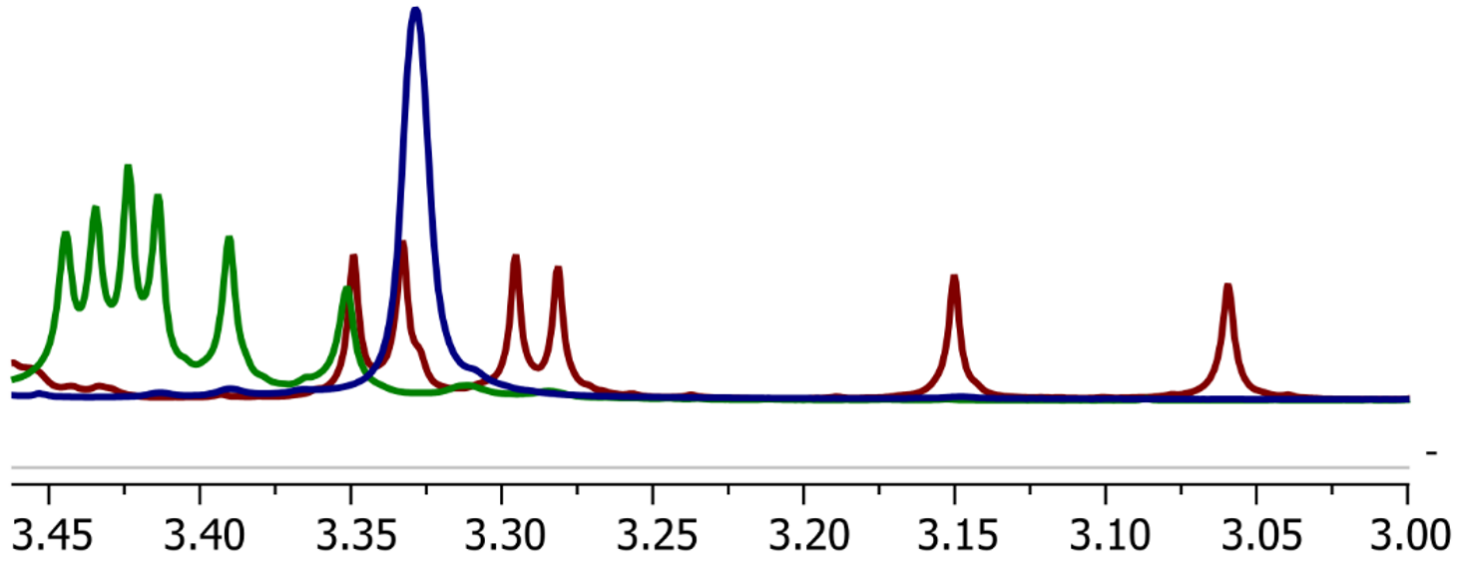
The fragment of the ^1^H NMR spectrum of compounds **9** (green); **10** (red); **12** (blue) representing the signals from Me–O–C^6^.

The spectra of **10** share several trends with **4**, including the scattering of hydrogen-6 signals, the interaction of triazole proton with several hydrogens inside the cavity (Figure 1b) on the NOESY spectrum, and the splitting of signals from methyl groups. However, the latter looks even more prominent on the spectrum since the methyls on the primary rim are near the aromatic system. Six separated singlets could be found in the 3.06–3.34 ppm range, where one peak represents one methyl group from one CD unit. Such observations may be explained by the desymmetrization of the molecule caused by a partial and reversible self-inclusion of the triazole moiety into the CD cavity, as was previously studied in detail for the CD dimers prepared by CuAAC reaction [15]. Although such self-inclusion was not prominent for dimers based on the short propargyl ether linker and unsubstituted CD, in our case, the Me–O–C^6^ groups could have changed the situation due to the steric hindrance. The interaction of the triazole proton with the protons inside the cavity was not observed in the spectra of compound **9,** which is acetyl-protected **10** (Figure 1c). However, it shares with **10** the same pattern of methyl splitting, though a much weaker one (Figure 2). We suppose that the chloroform where the spectrum of **9** was measured prevents the self-inclusion of the triazole ring.

Concluding all the abovementioned, we may assume that a type of spacer between two CD parts in a dimer could affect molecular symmetry, altering its binding ability. The influence of the triazole ring and its potential inclusion into the cavity must also be considered. Nevertheless, a recent study proved that the tumbling of the CD cavity is reversible and a strongly binding quest is capable of filling all CD cavities; thus, reversing the CD tumbling [33].

### The UV determination of tetracene solubility in solutions of supramolecular hosts

**Figure 3:**
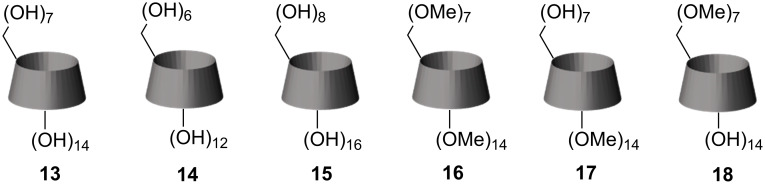
Other cyclodextrins that were used in the solubilization experiments with tetracene.

The obtained CD dimers with partially methylated rims, along with some other CD derivatives (Figure 3), were tested to increase the solubility of tetracene. Tetracene has limited solubility in several organic solvents such as chloroform, DMSO, THF, and chlorinated arenes [34–36]. Its solubility sufficiently increases with temperature, even more in a sealed vial where low boiling point solvents can be heated more. This way, we obtained concentrated hot solutions even in methanol and other solvents that almost do not dissolve tetracene at room temperature. DMSO demonstrated a moderate dissolving ability towards tetracene. Still, it does not have UV absorbance and dissolves all supramolecular hosts we prepared, making it the best choice for our study. Unfortunately, we did not succeed with solvents, such as water or methanol, where an increase in solubility is most desired. But some success we obtained with DMSO. For our experiments, we used the same mass of a host in every experiment, meaning the molar concentration of a CD in the samples was different. Especially it concerned the dimers whose solutions were approximately two times less concentrated than others. However, we decided from the practical point of view that the effectiveness of the solubilization effect should be calculated to the host's mass rather than its molar concentration.

**Figure 4:**
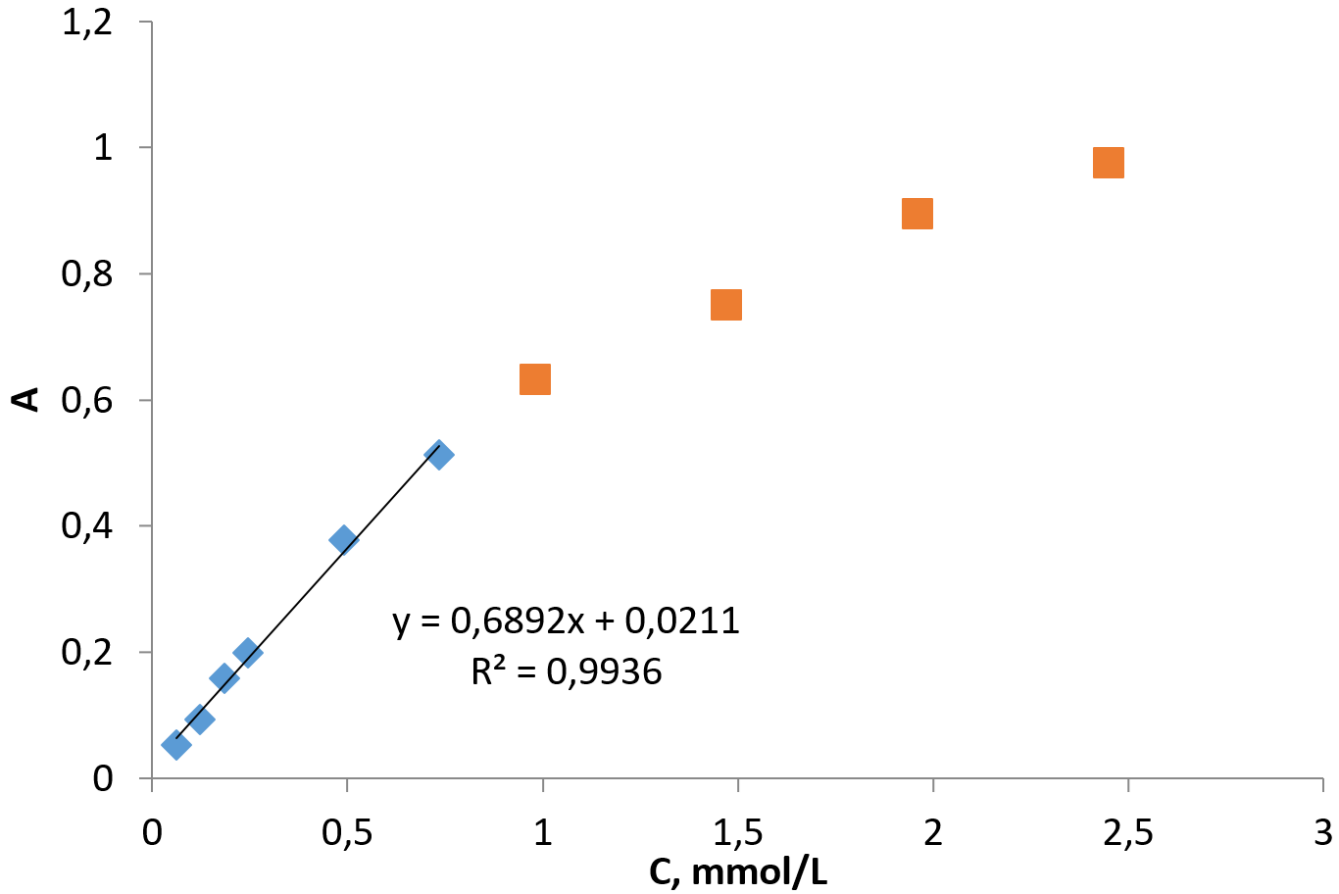
The tetracene UV absorbance dependence on concentration at 476 nm.

The UV absorbance spectrum of tetracene in DMSO consists of several regions gradually rising with the concentration. The absorption band at 476 nm is the best choice for building a calibration plot with a sufficient linear region (Figure 4). Exceeding the concentration above 1 mmol/L slowly decreases the linearity of the plot. Concentrated tetracene solutions with supramolecular hosts have UV absorbances out of the linear region. So we had to dilute them 5 and 10 times for the measurements.

**Figure 5:**
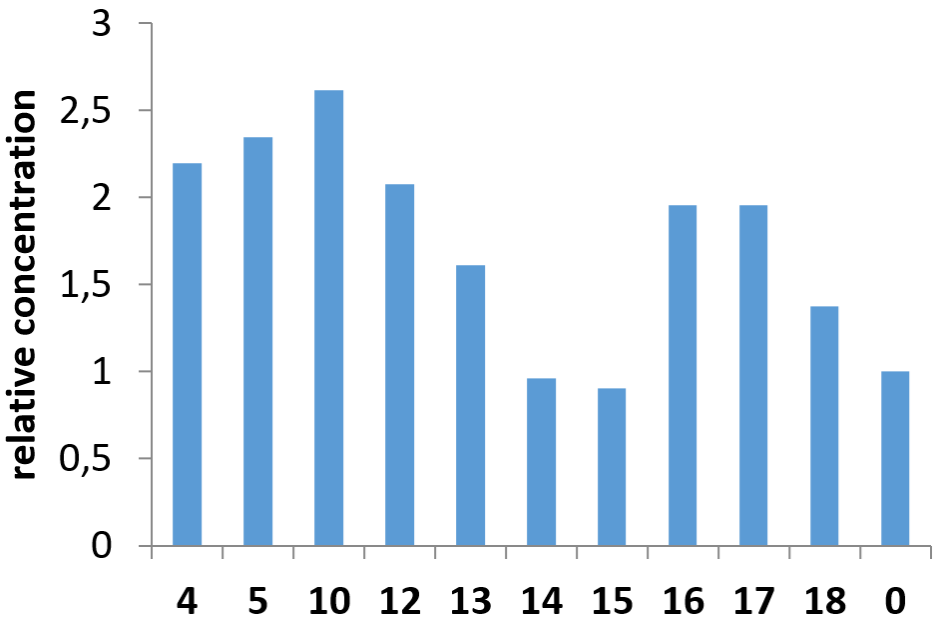
The relative concentrations of tetracene in DMSO solutions with hosts **4**, **5**, **10**, **12**, **13**–**18** referred to its concentrated solution without any hosts (**0**).

The relative concentration of tetracene in the presence of supramolecular hosts, relative to the concentration of tetracene in the saturated solution is shown in Figure 5. This chart implies an actual increase in solubility of tetracene for the majority of the tested samples. Since the size and shape of the cavities of α-CD and γ-CD are not comparable with tetracene's geometry, they do not provide any changes from the blank solution. The other compounds more or less enhanced the solubility. Ranging all β-CD derivatives from worst one to best one, we got the row:

**18** < **13** < **16** ≈ **17** < **12** < **4** < **5** < **10**

The same results recalculated to the molar concentration of hosts in the solutions (Table 1) do not change the order in this row. However, it highlights the difference between the "dimeric" and "monomeric" compounds, suggesting that both the dimers' CD parts are effectively involved in the complexation with tetracene. On the other hand, the recalculation equalizes the effectiveness of compounds **4**, **5,** and **10**, whereas compound **12** is still behind them.

**Table 1:** The calculated concentrations of tetracene in DMSO solutions with various hosts and the relative concentrations of tetracene in these solutions related to the concentrations of the corresponding hosts.

Host	The concentration of tetracene in the solution *c*, mM	The relative concentration of tetracene relative to the host concentration in the solution *c*/*c*_h_

**–**	3.11	–
**4**	6.85	0.38
**5**	7.3	0.39
**10**	8.14	0.42
**12**	6.47	0.32
**13**	5.01	0.11
**14**	3	0.08
**15**	2.81	0.05
**16**	6.09	0.17
**17**	6.08	0.16
**18**	4.28	0.11

**Figure 6:**
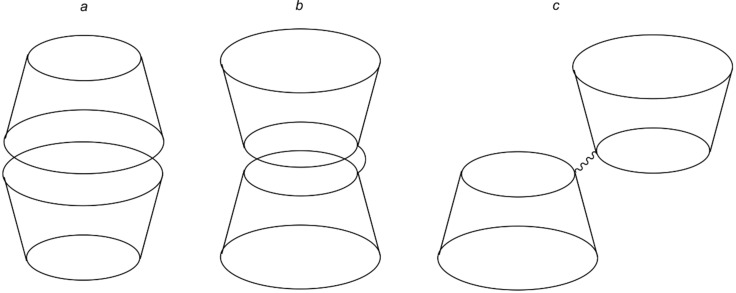
"Tail-to-tail" (a) and "head-to-head" (b) orientation of two cyclodextrin moieties and primary-rim connected CDs with non-interacting rims (c).

Comparing the dimers with the corresponding "monomers", one could find a notable difference in the performances of the compounds with methylated primary rims. The dimer **10** facilitates the dissolution of approximately 2 times more tetracene than **18**. Along with **18**, compound **13** (β-CD) has also shown poor solubilizing activity. We suggest that the presence of hydroxy groups on the secondary rim of CD allows the hydrogen bond formation between two molecules, creating a capsule (tail-to-tail interaction, Figure 6a). It seems the generation of such capsules in solution disfavors the complexation with tetracene. The compounds with occupied secondary rims, such as **16** and **17,** have shown better effectiveness, losing only to the dimers. A spacer connecting two primary rims in a dimer promotes the interaction between them, forming a capsule with the inverted orientation of CD moieties (head-to-head interaction, Figure 6b). We suppose this type of interaction results in better complexation with the guest.

The synthesized dimers have given us the best results in the experiment. However, it is quite difficult to highlight a trend in this small group because the dimers with methylated primary rims have shown the worst and best result, with a significant gap between their performances. In contrast, the dimers with methylated secondary rims have average solubilizing abilities. The phenomena we described above might be a reason for such behavior. The ^1^H NMR spectrum of compound **10** (Figure 2) indicates a significant degree of distortion in the molecule's symmetry. We suppose that the "tail-to-tail" interaction, which is unfavorable for the complexation with tetracene, does not take part in this case because the distorted secondary rims fail to build a system with strong hydrogen bonds. The spectrum of **12** clearly belongs to a compound with high symmetry, favoring this interaction and reducing the solubilization efficiency; also, the spacer length might be too short for the "head-to-head" orientation (Figure 6c).

### Determination of the binding parameters

Despite achieving some success in the increase of tetracene solubility, we struggled to evaluate the supramolecular interaction. Unfortunately, it seems that the complexation does not change the position of the chemical shifts on the NMR spectra and the shape or intensity of the UV–vis spectrum. The decrease in the guest's diffusion coefficient by binding with a high-molecular-weight host serves as a reliable indicator for the determination of binding parameters in a case if, for example, no alternation of chemical shifts is observed on the NMR spectrum. The diffusion coefficient can be estimated by a DOSY experiment [37]. In our case, a gradual, steady decline of tetracene's diffusion coefficient was detected by titration with **4**, but it exceeded the measurement error only at the maximum concentration of the CD. Thus, this method discovers a poor supramolecular interaction where a reliable determination of the binding parameters is impossible.

**Figure 7:**
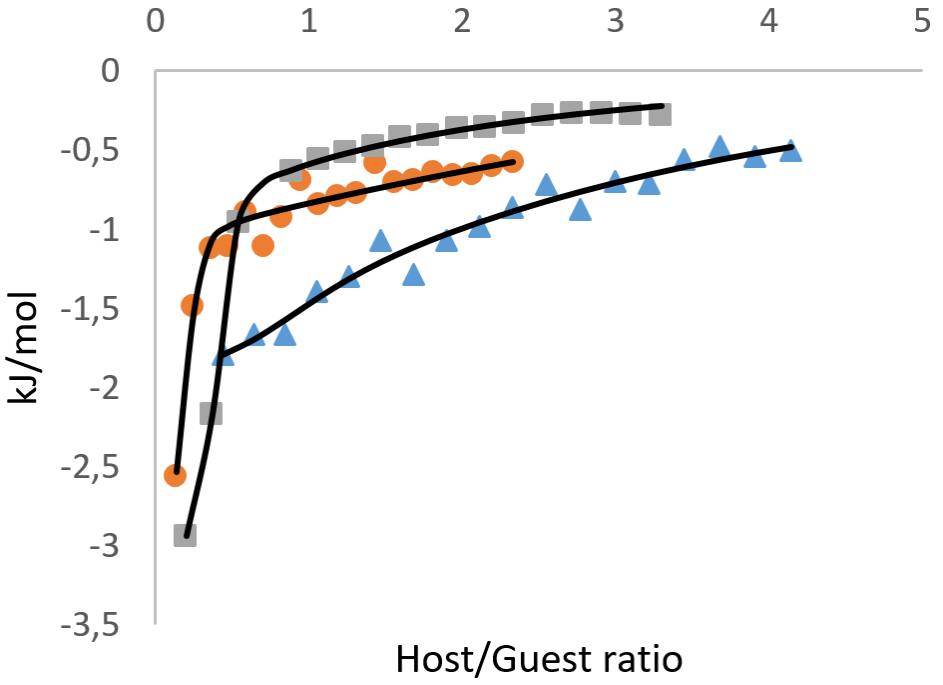
Isotherms of the titration of tetracene with "dimeric" CD solutions in DMSO at 298 K (circles – **10**; squares – **4**; triangles – **5**).

**Figure 8:**
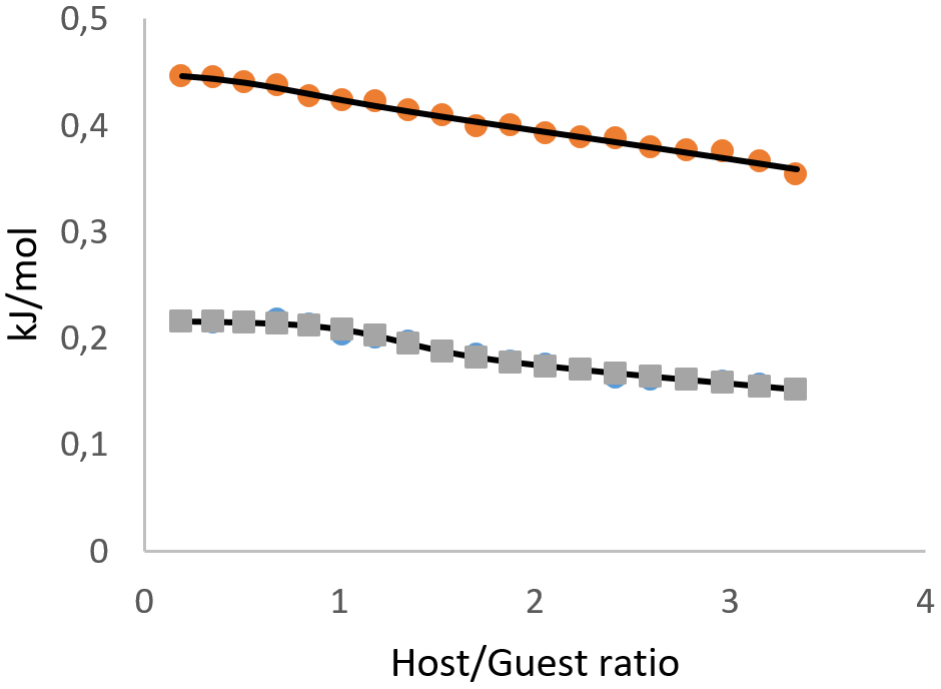
Isotherms of the titration of tetracene with "monomeric" CD solutions in DMSO at 298 K (circles – **16**; squares – **13**).

ITC (isothermal titration calorimetry) is a popular technique for evaluating supramolecular interaction with CDs [38], mainly in the aqueous medium. We performed the titrations of tetracene DMSO solution by the series of CDs used in the solubility experiments (Figure 7, Figure 8). The titrations were accompanied by measurable heat, different from the heat of dilution of both tetracene and CDs with pure solvent (see Supporting Information File 1). Therefore, the measured heat was attributed to the interactions of CDs with tetracene and the titration isotherms, which were successfully fitted to the 1:1 binding model.

The thermodynamic parameters for the interaction of CDs with tetracene are given in Table 2. The dilution of CDs to DMSO was exothermic for all five samples, with the lowest heat recorded for permethylated **16**. The dilution of CDs in an aqueous system, published elsewhere, is always endothermic [39]. The DMSO with 0.02% of water (roughly 11 mM, given by the manufacturer) was used. The dilution experiments demonstrated that DMSO does not absorb additional water in the timescale of the ITC experiment, which may produce measurement artifacts. Additionally, the titration of a DMSO to a DMSO with 5 vol % of water was carried out to manifest the contamination with water (see Supporting Information File 1). Therefore, the exothermic signal from the dilution of CDs in DMSO was attributed to the hydration of hydroxy and, to a lesser extent, methoxy groups from partially methylated CDs with the admixture of water in DMSO.

**Table 2:** The thermodynamic parameters of the binding calculated from ITC experiments.

**CD**	Stoichiometry *n*	Affinity constant, *K*_a _^.^10^−4^, M^−1^	Gibbs free energy Δ*G*, kJ/mol	Enthalpy Δ*H*, kJ/mol	Entropy Δ*S,* J/mol^.^K

**13**	1.22	2.6	−25.2	0.2	85.3
**16**	0.72	1.3	−23.5	0.5	79.9
**10**	0.15	10.9	−28.7	−2.9	86.6
**4**	0.32	8.6	−28.2	−3.1	84.1
**5**	0.83	0.7	−21.9	−2.0	66.5

All CDs interactions with tetracene were accompanied by sufficient entropy gain, which points to the desolvation of interacting molecules. The interaction of CD "monomers" **13** and **16** with tetracene is weakly endothermic and, therefore, understood as an entropy-driven process with the potential formation of a complex in 1:1 stoichiometry. All CD dimers interacted with tetracene exothermically. Since the overall amount of water in the system is one order lower than the amount of hydroxy groups in CDs, they preserve the capacity for hydrogen bonding. The presence of tetracene in the solution enhances inter- or intramolecular associations of CD rings in dimers. The CD dimer **5**, linked with urea, demonstrated stoichiometry close to unity. Still, dimers **4** and **10**, linked with triazole-based spacer, exhibited strong exothermic interaction at an unusual stoichiometry range of 0.15–0.32 (3 to 7 tetracene molecules to one CD dimer). These two CD dimers, according to NMR, have the triazole linker partially included in CD rings. We assume that their interaction with tetracene is a multistep process, and the obtained thermodynamic parameters are the effective values for the sum of all interaction steps.

Although from the sole ITC experiment, the formation of tetracene inclusion complex with CDs can't be affirmed, the observed stoichiometry favors this hypothesis. Additionally, the binding strength in terms of *K*_a_ (M^−1^) well matches the solubility experiments' results and emphasizes the exceptional performance of the synthesized dimers.

## Conclusion

In conclusion, we have prepared four new CD dimers from 6-azido partially methylated β-CD derivatives with two types of spacers between CD moieties. A new method for preparing β-CDs with selectively methylated primary rim based on the remarkable methylation ability of methyl tosylate with relatively mild bases at solvent-free conditions has been developed. It has been proved that using Lewis acid in the phosphine imide reactions with CDs can increase the reactivity of some low-reactive compounds, giving better yields and reducing the reaction time. The UV measurements have confirmed an increase in solubility of tetracene in DMSO in the presence of some CD derivatives. The partially methylated CD dimers are more than twice effective in solubilization than the compounds with one CD unit in the molecule in the molar ratio, implying that both CD fragments are engaged in complexation with tetracene. The compounds possessing hydroxy groups on the secondary rim generally perform worse than those with a methylated secondary rim. ITC studies and tetracene's increased solubility prove the host–guest interaction's existence. The obtained results give hope for a successful continuation of the study with higher acenes.

## Experimental

**Materials.** α- (**14**), β- (**13**), and γ-cyclodextrin (**15**) were purchased from Wacker Chemie AG; methyl tosylate was purchased from Acros Organics; the remaining chemicals were bought from Merck. The methyl tosylate contained traces of *p*-toluenesulfonic acid, so, before use, we washed it with saturated NaHCO_3_ solution in a dropping funnel and dried it over anhydrous MgSO_4_. The other chemicals for synthesis were used without further purification. SiliaFlash P60 40–63 μm from SiliCycle was used for column chromatography. The solvents were supplied by Penta and were distilled before use. The course of the reactions was followed on TLC Silica gel 60 F_254_ bought from Merck company. For the UV measurements, α-, β-, and γ-cyclodextrin were recrystallized from hot water or a water/methanol mixture. The purity of the prepared products was considered enough for use in UV experiments without additional purification.

**Methods.** Low-resolution mass spectra were measured with a Shimadzu LCMS-2020 spectrometer. Samples were ionized by electrospray technique (ESI) and detected by quadrupole or TOF. The drying and nebulizer gas was nitrogen. High-resolution mass spectra were measured with an Agilent Technologies 6530 Accurate-Mass Q-TOF LC/MS spectrometer. Samples were ionized by electrospray technique (ESI) and detected by quadrupole or TOF. UV–vis spectroscopy spectra were measured with Thermo Scientific Helios γ with wolfram and deuterium lamp. The wavelength range is 190–800 nm. ^1^H, ^13^C, and 2D NMR spectra were measured on Bruker Avance III HD 400 spectrometer. For TLC detection of CDs, we charred a TLC plate with 50% sulfuric acid water solution at 250 °C.

### UV measurements

#### The determination of tetracene's solubility in DMSO

To determine the solubility of tetracene in DMSO, we put a small amount of tetracene (2.0–2.5 mg) in a vial, added 2 mL of DMSO, and heated the vial until the whole compound had been dissolved. Then we cooled down the vial in a dark place, and after 1 hour, we filtered off the formed crystals, took 50 and 100 μL of the solution, diluted them to 1 mL, and measured the UV spectra. A comparison with the calibration plot determined the concentration of the tetracene. Thus, we estimated tetracene's solubility in DMSO to be 0.71 mg/mL at room temperature.

#### The calibration of tetracene UV absorption dependence

To determine the UV absorbance dependence of tetracene on its concentration, we prepared a stock solution containing 2.8 mg of tetracene in 5 mL of DMSO. The stock solution was then used to prepare a series of solutions with different concentrations (see the Supporting Information File 1). The solutions' light absorbance at 476 nm was used to build the calibration plot.

#### The determination of tetracene's solubility in DMSO in the presence of the supramolecular hosts

To determine how a supramolecular host affects the guest's solubility, we used the same procedure for preparing the concentrated tetracene solution. We used 3 mg of tetracene and 50 mg of a host for every sample, dissolved in 1 mL of DMSO. The samples were heated until the compounds were fully dissolved and let cool down. The undissolved tetracene was filtered off, and the samples were diluted 5 and 10 times to get the absorbance of the solution into the linear range of the tetracene concentration/UV absorption calibration plot.

#### ITC measurements

The saturated solution of tetracene (0.71 mg/mL, 3.1 mM) was titrated at 298 K with the solutions of cyclodextrins: **13** (57 mg/mL; 50 mM), **16** (71 mg/mL, 50 mM), **10** (27 mM; 70 mg/mL), **4** (41 mM, 114 mg/mL), **5** (48 mM, 129 mg/mL) in DMSO on MicroCal ITC200 (Malvern Panalytical Ltd, UK) isothermal titration calorimeter. The titrations were performed in 20 consecutive injections, where the first injection of 0.4 μL was followed by 19 injections of 2 μL of CD solution. The blank titrations of CDs to the solvent were performed similarly, and the heat of dilution was subtracted from the corresponding isotherms. The isotherms were fitted with ITC 200 1.25.5 (Malvern Panalytical Ltd, UK) software, based on Origin 7SR4 v 7.0552 (OriginLab Corporation, MA, USA), to the two independent binding sites model. From the fit, the stoichiometry (*n*), binding enthalpy change (Δ*H*, kJ·mol^−1^), affinity constant (*K*_a_, M^−1^), binding free energy change (Δ*G*, kJ·mol^−1^), and binding entropy change (Δ*S*, J·mol^–1^·K^−1^) for the first binding site were calculated. The thermodynamic parameters for the second binding site were not discussed but used to subtract the nonlinear residual heat at high molar ratios. Separate titration experiments of pure DMSO to the solution of tetracene in DMSO and DMSO to the solution of water in DMSO were carried out to account for potential measurement artifacts originating from water concentration mismatch in highly hygroscopic DMSO.

## Supporting Information

File 1 Synthetic procedures, characterization, ^1^H, ^13^C DEPT, 2D NMR, IR, UV–vis spectra of synthesized compounds; UV–vis spectra of tetracene solutions in DMSO; ITC thermograms.

## References

[1] Del Valle E M M (2004). Process Biochem (Oxford, U K).

[2] Wenz G (2012). Beilstein J Org Chem.

[3] Řezanka M (2019). Environ Chem Lett.

[4] Connors K A (1997). Chem Rev.

[5] Kida T, Fujino Y, Miyawaki K, Kato E, Akashi M (2009). Org Lett.

[6] Kida T, Iwamoto T, Fujino Y, Tohnai N, Miyata M, Akashi M (2011). Org Lett.

[7] Breslow R, Greenspoon N, Guo T, Zarzycki R (1989). J Am Chem Soc.

[8] Breslow R, Chung S (1990). J Am Chem Soc.

[9] Breslow R, Zhang B (1994). J Am Chem Soc.

[10] Breslow R, Zhang B (1996). J Am Chem Soc.

[11] Liu Y, Chen Y (2006). Acc Chem Res.

[12] Mourer M, Hapiot F, Monflier E, Menuel S (2008). Tetrahedron.

[13] Mourer M, Hapiot F, Tilloy S, Monflier E, Menuel S (2008). Eur J Org Chem.

[14] Blaszkiewicz C, Bricout H, Léonard E, Len C, Landy D, Cézard C, Djedaïni-Pilard F, Monflier E, Tilloy S (2013). Chem Commun.

[15] Potier J, Menuel S, Azaroual N, Monflier E, Hapiot F (2014). Eur J Org Chem.

[16] Hamon F, Blaszkiewicz C, Buchotte M, Banaszak-Léonard E, Bricout H, Tilloy S, Monflier E, Cézard C, Bouteiller L, Len C (2014). Beilstein J Org Chem.

[17] Chaudhuri S, Verderame M, Mako T L, Bandara Y M N D Y, Fernando A I, Levine M (2018). Eur J Org Chem.

[18] Anthony J E (2008). Angew Chem, Int Ed.

[19] Zade S S, Bendikov M (2010). Angew Chem, Int Ed.

[20] Nagano M, Hasegawa T, Myoujin N, Yamaguchi J, Itaka K, Fukumoto H, Yamamoto T, Koinuma H (2004). Jpn J Appl Phys, Part 1.

[21] Jancarik A, Levet G, Gourdon A (2019). Chem – Eur J.

[22] Payne M M, Parkin S R, Anthony J E (2005). J Am Chem Soc.

[23] Takeo K, Ueraura K, Mitoh H (1988). J Carbohydr Chem.

[24] Takeo K, Mitoh H, Uemura K (1989). Carbohydr Res.

[25] Varga E, Benkovics G, Darcsi A, Várnai B, Sohajda T, Malanga M, Béni S (2019). Electrophoresis.

[26] Carofiglio T, Cordioli M, Fornasier R, Jicsinszky L, Tonellato U (2004). Carbohydr Res.

[27] Tungala K, Adhikary P, Krishnamoorthi S (2013). Carbohydr Polym.

[28] Munteanu M, Choi S, Ritter H (2009). Macromolecules.

[29] Cintas P, Barge A, Tagliapietra S, Boffa L, Cravotto G (2010). Nat Protoc.

[30] Sallas F, Marsura A, Petot V, Pintér I, Kovács J, Jicsinszky L (1998). Helv Chim Acta.

[31] Menuel S, Porwanski S, Marsura A (2006). New J Chem.

[32] Cravotto G, Fokin V V, Garella D, Binello A, Boffa L, Barge A (2010). J Comb Chem.

[33] González-Méndez I, Hameau A, Laurent R, Bijani C, Bourdon V, Caminade A-M, Rivera E, Moineau-Chane Ching K I (2020). Eur J Org Chem.

[34] Liang Z, Zhao W, Wang S, Tang Q, Lam S-C, Miao Q (2008). Org Lett.

[35] Kim H Y, Bjorklund T G, Lim S-H, Bardeen C J (2003). Langmuir.

[36] Griffini G, Brambilla L, Levi M, Castiglioni C, Del Zoppo M, Turri S (2014). RSC Adv.

[37] Ferrazza R, Rossi B, Guella G (2014). J Phys Chem B.

[38] Mura P (2014). J Pharm Biomed Anal.

[39] Zheng P J, Wang C, Hu X, Tam K C, Li L (2005). Macromolecules.

