# Supplementary material for: Preparation of β-cyclodextrin-based dimers with selectively methylated rims and their use for solubilization of tetracene
Source: Beilstein J Org Chem. 2022 Nov 25;18:1596–606. doi: 10.3762/bjoc.18.170 (PMC9704020; doi:10.3762/bjoc.18.170)
Supplement: File 1 — Synthetic procedures, characterization, 1H, 13C DEPT, 2D NMR, IR, UV–vis spectra of synthesized compounds; UV–vis spectra of tetracene solutions in DMSO; ITC thermograms. [file Beilstein_J_Org_Chem-18-1596-s001.pdf]

BEILSTEIN JOURNAL OF ORGANIC CHEMISTRY

Supporting Information

for

Preparation of β-cyclodextrin-based dimers with selectively methylated rims and their use for solubilization of tetracene

Konstantin Lebedinskiy, Volodymyr Lobaz and Jindřich Jindřich

Beilstein J. Org. Chem. **2022,** *18,* 1596–1606. doi:10.3762/bjoc.18.170

Synthetic procedures, characterization, ^{1}H, ^{13}C DEPT, 2D NMR, IR, UV–vis spectra of synthesized compounds; UV–vis spectra of tetracene solutions in DMSO; ITC thermograms

License and Terms: This is a supporting information file under the terms of the Creative Commons Attribution License (https://creativecommons.org/licenses/by/4.0). Please note that the reuse, redistribution and reproduction in particular requires that the author(s) and source are credited and that individual graphics may be subject to special legal provisions.
The license is subject to the *Beilstein Journal of Organic Chemistry* terms and conditions: (https://www.beilstein-journals.org/bjoc/terms)

Contents

I Synthesis and characterization S5

6^A-Azido-6^A-deoxy-$6^{B,C,D,E,F,G}$-*O*-hexakis-tert-butyldimethylsilyl-β-cyclodextrin (**1**)..... S5

Fig. S1 ^{1}H NMR spectrum of **1** in CDCl3 at 25 °C S6

Fig. S2 DEPT-^{13}C NMR spectrum of **1** in $CDCl_3$ at 25 °C..... S7

Fig. S3 COSY spectrum of **1** in $CDCl_3$ at 25 °C..... S8

Fig. S4 HSQC spectrum of **1** in $CDCl_3$ at 25 °C S9

Fig. S5 IR spectrum of **1** in KBr pellet S10

Fig. S6 UV–vis spectrum of **1** in $CHCl_3$..... S10

6^A-Azido-6^A-deoxy-$6^{B,C,D,E,F,G}$-*O*-hexakis-*tert*-butyldimethylsilyl-per-2,3-*O*-methyl-β-cyclodextrin (**2**) . S11

Fig. S7 ^{1}H NMR spectrum of **2** in $CDCl_3$ at 25 °C S12

Fig. S8 DEPT-^{13}C NMR spectrum of **2** in $CDCl_3$ at 25 °C..... S13

Fig. S9 COSY spectrum of **2** in $CDCl_3$ at 25 °C..... S14

Fig. S10 HSQC spectrum of **2** in $CDCl_3$ at 25 °C S15

Fig. S11 IR spectrum of **2** in KBr pellet S16

Fig. S12 UV–vis spectrum of **2** in $CHCl_3$..... S16

6^A-Azido-6^A-deoxy-per-2,3-*O*-methyl-β-cyclodextrin (**3**) S17

Fig. S13 ^{1}H NMR spectrum of **3** in $CDCl_3$ at 25 °C S18

Fig. S14 DEPT-^{13}C NMR spectrum of **3** in $CDCl_3$ at 25 °C..... S19

Fig. S15 COSY spectrum of **3** in $CDCl_3$ at 25 °C..... S20

Fig. S16 HSQC spectrum of **3** in $CDCl_3$ at 25 °C S21

Fig. S17 IR spectrum of **3** in KBr pellet S22

Fig. S18 UV–vis spectrum of **3** in $CDCl_3$..... S22

Bis-1,3-[1-(6^A-deoxy-2,3-per-*O*-methyl-β-cyclodextrin-6^A-*C*-yl)-1,2,3-triazol-4-yl]methoxymethylene (**4**) S23

Fig. S19 ^{1}H-NMR spectrum of **4** in D_2O at 25 °C S24

Fig. S20 DEPT-^{13}C-NMR spectrum of **4** in D_2O at 25 °C S25

Fig. S21 COSY spectrum of **4** in D_2O at 25 °C S26

Fig. S22 HSQC spectrum of **4** in D_2O at 25 °C S27

Fig. S23 NOESY spectrum of **4** in D_2O at 25 °C S28

Fig. S24 IR spectrum of **4** in KBr pellet S29

Fig. S25 UV–vis of **4** in $CHCl_3$ S29

N,*N'*- Bis(6^A-deoxy-2,3-per-*O*-methyl-β-cyclodextrin-6^A-*C*-yl)urea (**5**) S30

Fig. S26 ^{1}H-NMR spectrum of **5** in D_2O at 25 °C S30

Fig. S27 DEPT-^{13}C-NMR spectrum of **5** in D_2O at 25 °C S31

Fig. S28 COSY spectrum of **5** in D_2O at 25 °C S32

Fig. S29 HSQC spectrum of **5** in D_2O at 25 °C S33

Fig. S30 IR spectrum of **5** in KBr pellet S34

Fig. S31 UV–vis spectrum of **5** in $CHCl_3$ S34

6^A-Azido-6^A-deoxy-$6^{B,C,D,E,F,G}$-*O*-hexakis-*tert*-butyl-dimethylsilyl-per-2,3-*O*-acetyl-β-cyclodextrin (**6**) .. S35

Fig. S32 ^{1}H-NMR spectrum of **6** in $CHCl_3$ at 25 °C S36

Fig. S33 DEPT-^{13}C-NMR spectrum of **6** in $CHCl_3$ at 25 °C S37

Fig. S34 COSY spectrum of **6** in $CHCl_3$ at 25 °C S38

Fig. S35 HSQC spectrum of **6** in $CHCl_3$ at 25 °C S39

Fig. S36 IR spectrum of **6** in KBr pellet S40

Fig. S37 UV–vis spectrum of **6** in $CHCl_3$ S40

6^A-Azido-6^A-deoxy-per-2,3-*O*-acetyl-β-cyclodextrin (**7**) S41

Fig. S38 ^{1}H-NMR spectrum of **7** in [d_4]-MeOH at 25 °C S42

Fig. S39 DEPT-^{13}C-NMR spectrum of **7** in [d_4]-MeOH at 25 °C S43

Fig. S40 COSY spectrum of **7** in [d_4]-MeOH at 25 °C S44

Fig. S41 HSQC spectrum of **7** in [d_4]-MeOH at 25 °C S45

Fig. S42 IR spectrum of **7** in KBr pellet S46

Fig. S43 UV–vis spectrum of **7** in $CHCl_3$ S46

6^A-Azido-6^A-deoxy-$6^{B,C,D,E,F,G}$-*O*-hexakis-methyl-per-2,3-*O*-acetyl-β-cyclodextrin (**8**) S47

Fig. S44 ^{1}H-NMR spectrum of **8** in $CDCl_3$ at 25 °C S48

Fig. S45 DEPT-^{13}C-NMR spectrum of **8** in $CDCl_3$ at 25 °C S49

Fig. S46 COSY spectrum of **8** in $CDCl_3$ at 25 °C S50

Fig. S47 HSQC spectrum of **8** in $CDCl_3$ at 25 °C S51

Fig. S48 IR spectrum of **8** in KBr pellet S52

Fig. S49 UV–vis spectrum of **8** in $CHCl_3$ S52

Bis-1,3-[1-(6^A-deoxy-$6^{B,C,D,E,F,G}$-*O*-hexakis-methyl-per-2,3-*O*-acetyl-β-cyclodextrin-6^A-*C*-yl)-1,2,3-triazol-4-yl]methoxymethylene (**9**) S53

Fig. S50 ^{1}H-spectrum of **9** in $CDCl_3$ at 25 °C S54

Fig. S51 DEPT-^{13}C-NMR spectrum of **9** in $CDCl_3$ at 25 °C S55

Fig. S52 COSY spectrum of **9** in $CDCl_3$ at 25 °C S56

Fig. S53 HSQC spectrum of **9** in $CDCl_3$ at 25 °C S57

Fig. S54 NOESY spectrum of **9** in $CDCl_3$ at 25 °C S58

Bis-1,3-[1-(6^A-deoxy-$6^{B,C,D,E,F,G}$-*O*-hexakis-methyl-β-cyclodextrin-6^A-yl)-1,2,3-triazol-4-yl]methoxymethylene (**10**) S59

Fig. S55 ^{1}H-NMR spectrum of **10** in D_2O at 25 °C S60

Fig. S56 DEPT-^{13}C-NMR spectrum of **10** in D_2O at 25 °C S61

Fig. S57 COSY spectrum of **10** in D_2O at 25 °C S62

Fig. S58 HSQC spectrum of **10** in D_2O at 25 °C S63

Fig. S59 NOESY spectrum of **10** in D_2O at 25 °C S64

Fig. S60 IR spectrum of **10** in KBr pellet S65

Fig. S61 UV–vis spectrum of **10** in H_2O S65

6-Azido-6-deoxy -6-per-O-methyl -β-cyclodextrin (**11**) S66

Fig. S62 ^{1}H-NMR spectrum of **11** in [d_6]-DMSO at 25 °C ... S66

Fig. S63 DEPT-^{13}C-NMR spectrum of **11** in [d_6]-DMSO at 25 °C ... S67

Fig. S64 COSY spectrum of **11** in [d_6]-DMSO at 25 °C ... S68

Fig. S65 HSQC spectrum of **11** in [d_6]-DMSO at 25 °C ... S69

Fig. S66 IR spectrum of **11** in KBr pellet ... S70

Fig. S67 UV–vis spectrum of **11** in DMSO ... S70

N,N'-Bis(6^{A}-deoxy-$6^{B,C,D,E,F,G}$-*O*-hexakis-methyl-β-cyclodextrin-6^{A}-C-yl)urea (12) ... S71

Fig. S68 ^{1}H-NMR spectrum of **12** in D_2O at 25 °C ... S72

Fig. S69 DEPT-^{13}C-NMR spectrum of **12** in D_2O at 25 °C ... S73

Fig. S70 COSY spectrum of **12** in D_2O at 25 °C ... S74

Fig. S71 HSQC spectrum of **12** in D_2O at 25 °C ... S75

Fig. S72 IR spectrum of **12** in KBr pellet ... S76

Fig. S73 UV–vis spectrum of **12** in DMSO ... S76

II UV experiments with tetracene ... S77

Fig. S74 UV–vis spectrum of tetracene in DMSO at different concentrations ... S77

Fig. S75 Concentration dependence of tetracene's light absorbance in DMSO at 476 nm ... S77

Fig. S76 UV–vis spectrum of 5-times diluted saturated tetracene solutions with the studied CDs and without any supramolecular host (O) ... S78

Fig. S77 UV–vis spectrum of 10-times diluted saturated tetracene solutions with the studied CDs and without any supramolecular host (O) ... S78

III ITC experiments ... S79

Fig. 78 ITC thermograms ... S79

IV References for SI ... S81

I Synthesis and characterization

6-Azido-6-deoxy-β-cyclodextrin was prepared by two-step synthesis, starting with the tosylation of β-cyclodextrin [1] and subsequent azidation [2]. Per-6-*O*-methyl-β-cyclodextrin (**18**) and per-2,3-*O*-methyl-β-cyclodextrin (**17**) were prepared according to the procedures described in [3]. Per-2,3,6-*O*-methyl-β-cyclodextrin (**16**) was synthesized according to the method proposed by Zhou [4].

6^{A}-Azido-6^{A}-deoxy-hexakis-$6^{B,C,D,E,F,G}$-*O*-*tert*-butyldimethylsilyl-β-cyclodextrin (**1**)

6^{A}-Azido-6^{A}-deoxy-β-CD (5.6 g, 4.828 mmol) was dried under vacuum at 85 °C for 3 h. Then, imidazole (4.8 g, 63.7252 mmol) was added, and the mixture was dissolved in dry DMF (100 mL). The reaction mixture was stirred under argon. A portion of *tert*-butyldimethylsilyl chloride (4.8 g, 31.863 mmol), dissolved in THF (15 mL), was added dropwise to the reaction mixture. The course of the reaction was followed by TLC (EtOAc/EtOH/H_2O 30/5/4) every 15 min after 1 h since the start of the reaction. The main product has R_F =0.6, while all spots with lower R_F represent undersilylated compounds and all spots with higher R_F indicate oversilylated compounds. The reaction was allowed to proceed for approximately 1.5 h until all undersilylated cyclodextrins had disappeared. Then, the reaction mixture was poured into cold water, and the bulky precipitate was filtered and washed several times on the frit with distilled water. The powder-like solid was put into a Petri dish and moved to a desiccator for several days. The compound was dried under a vacuum and dissolved in 50 mL of methanol under heating. Then the compound was precipitated by the addition of 200 mL of acetonitrile. The compound was filtered off, and the recrystallization process was repeated until all by-products had disappeared. After drying, we obtained 3.32 g of pure product (79% yield).

^1H-NMR (400 MHz, $CDCl_3$) δ=6.57-6.83 (H-O^2, 7H); 5.2-5.33 (H-O^3, 7H); 4.88-4.98 (H-C^1, 7H); 4.01-4.09 (H-C^4; 3.35-4.09 H-C^3, H-C^5, H-C^4, H-C^2); 0.89 (*t*-butyl-Si, 54H); 0.06 (methyl-Si, 36H). ^{13}C-NMR (100 MHz, $CDCl_3$) δ=101,64-102,32 (C^1); 81.16-83.16 (C^2); 70.44-73.86 (C^3, C^4, C^5); 61.38-62.62 ($C^{6B,C,D,E,F,G}$); 51.47 (C^{6A}); 25.78 (C-*t*-butyl-Si); -5 (C-methyl-Si); HRMS (ESI): *m/z* calcd for $C_{78}H_{153}N_3O_{34}Si_6$+$Na^+$: 1866,88 [M+$Na^+$]; found: 1866.88; [α]$CHCl_3$= +114.3° (the data are consistent with the literature [5]).

Fig. S1 ^1H NMR spectrum of **1** in CDCl3 at 25 °C.

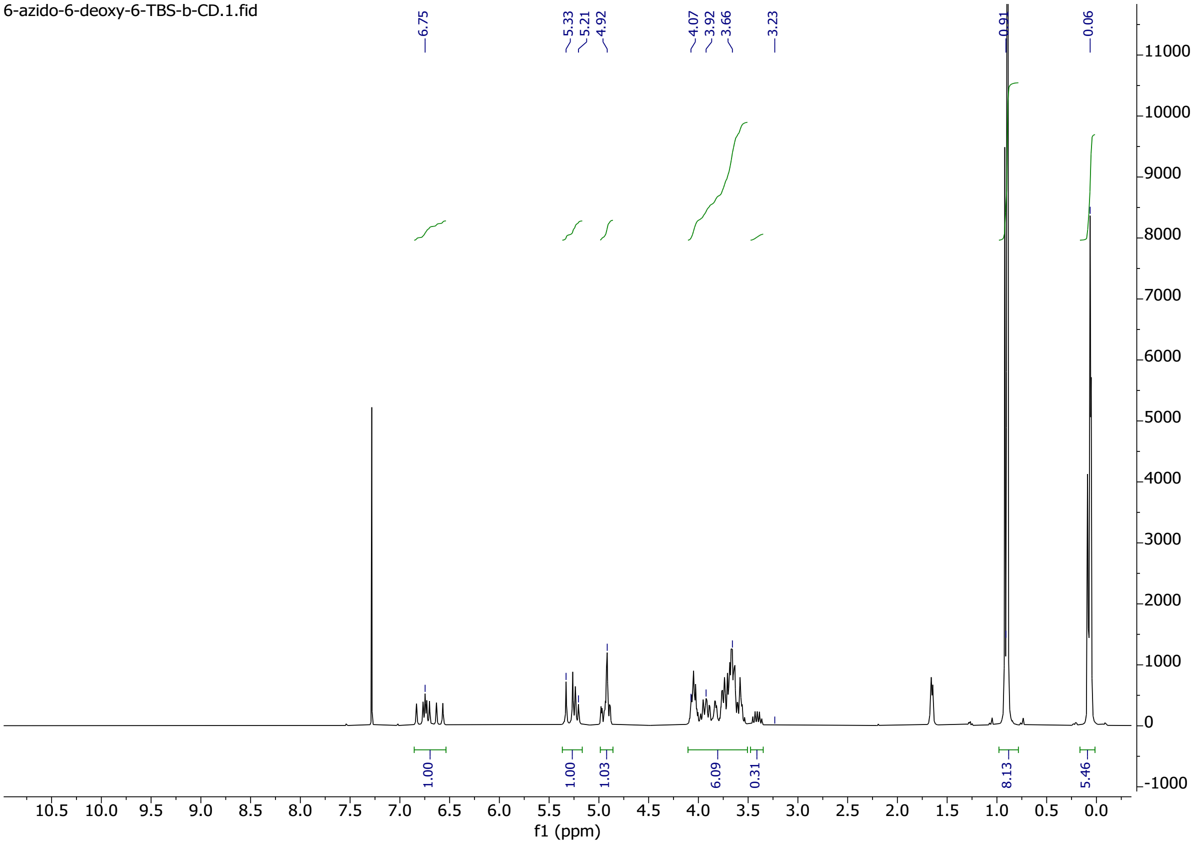

Fig. S2 DEPT-^{13}C NMR spectrum of **1** in $CDCl_3$ at 25 °C.

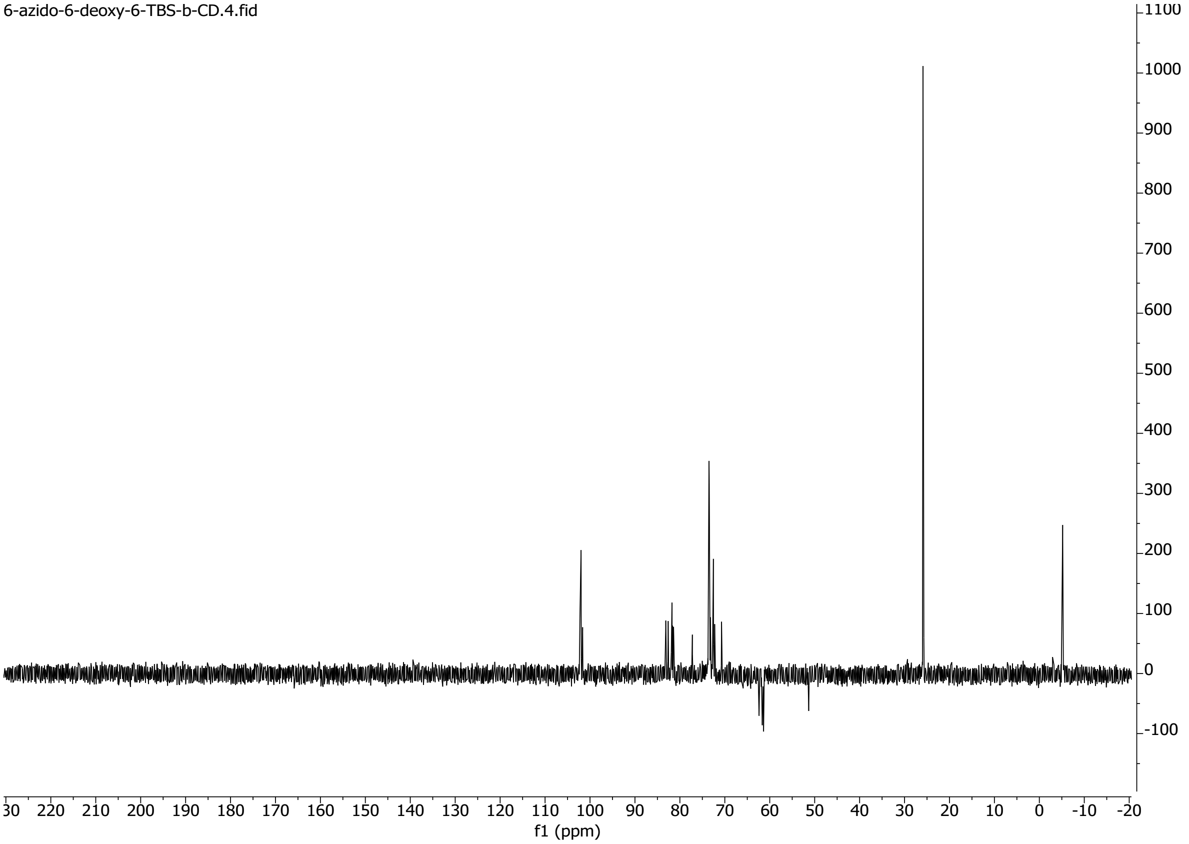

Fig. S3 COSY spectrum of **1** in $CDCl_3$ at 25 °C.

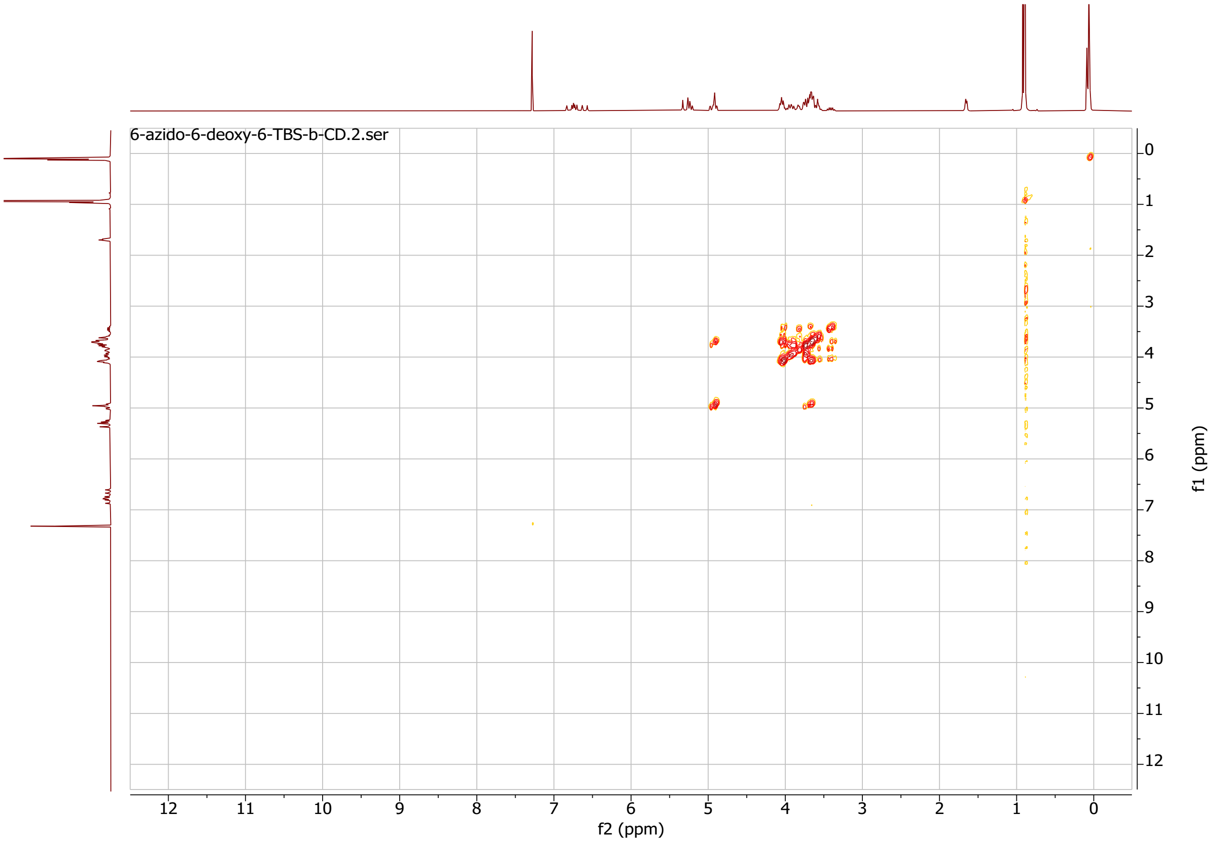

Fig. S4 HSQC spectrum of **1** in $CDCl_3$ at 25 °C.

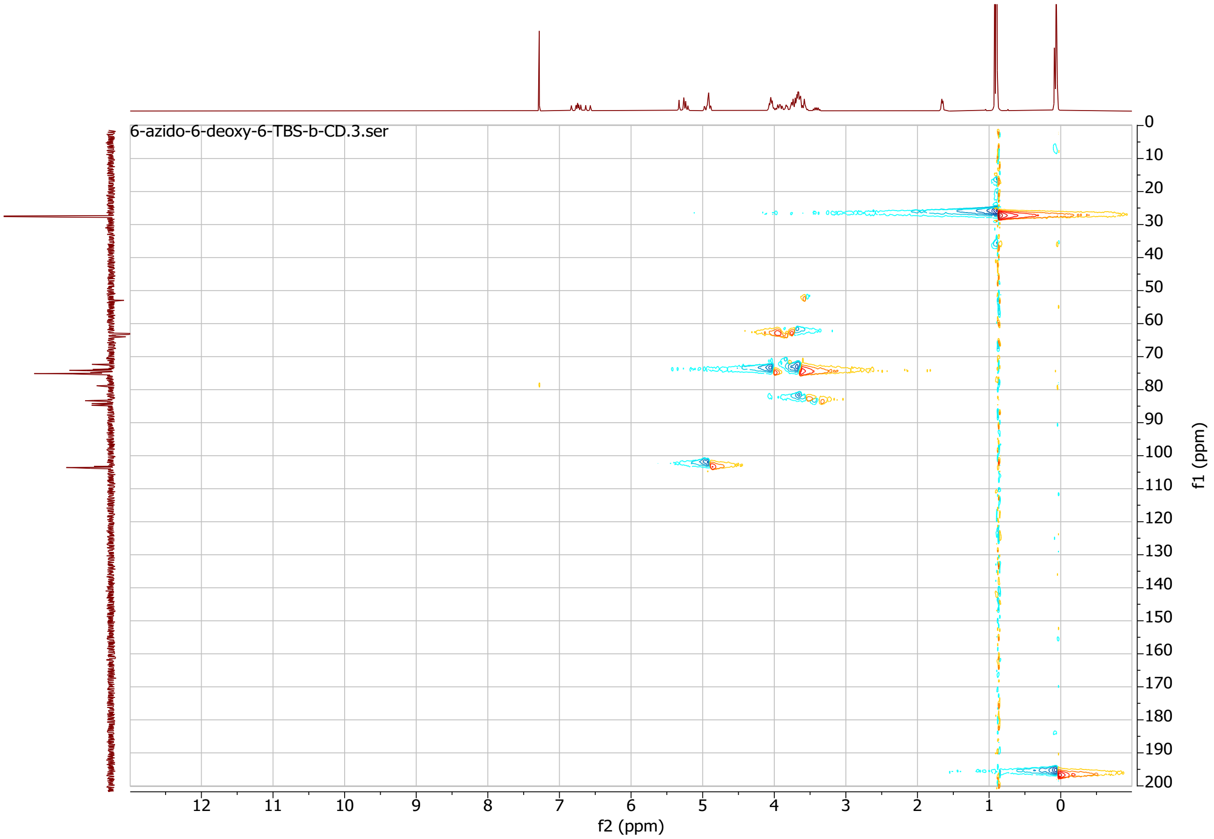

Fig. S5 IR spectrum of **1** in KBr pellet.

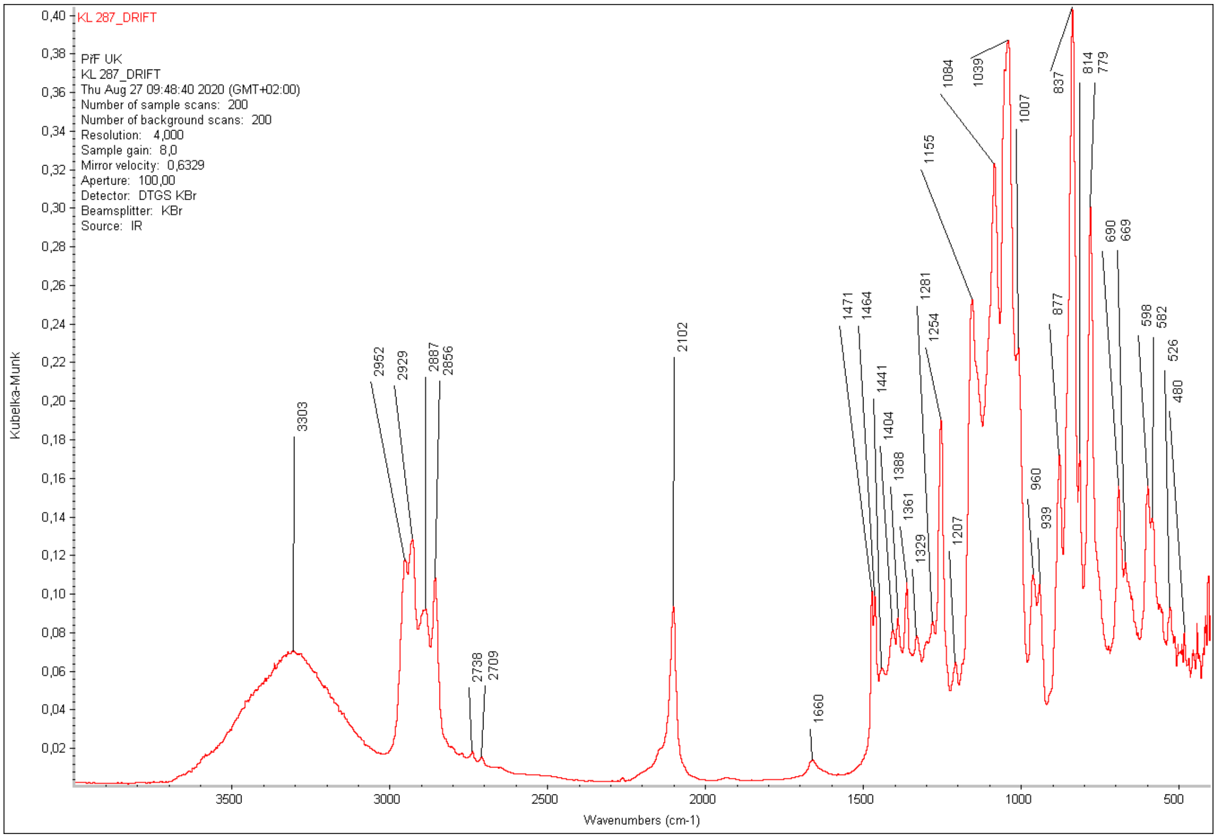

Fig. S6 UV–vis spectrum of **1** in $CHCl_3$.

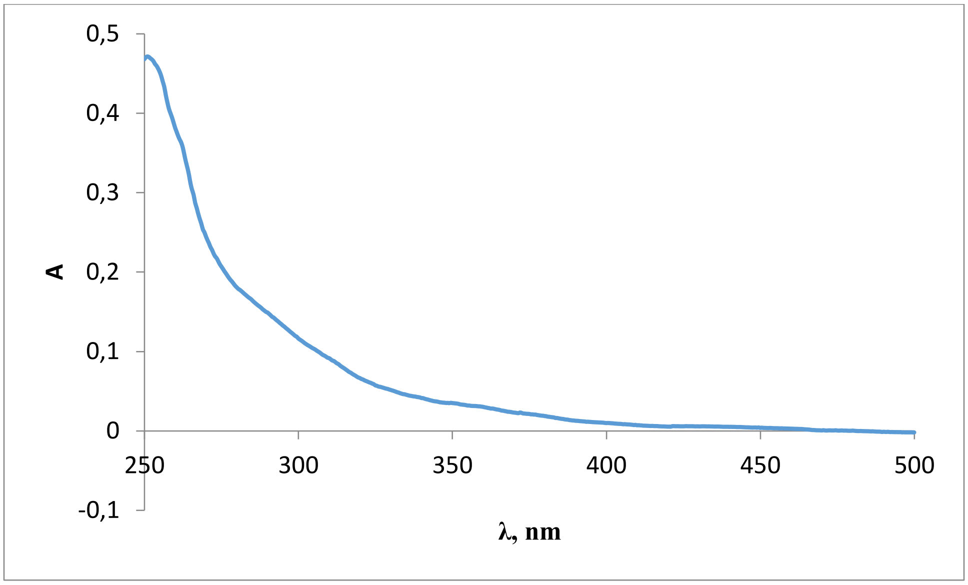

6^A-Azido-6^A-deoxy-hexakis-$6^{B,C,D,E,F,G}$-*O*-*tert*-butyldimethylsilyl-per-2,3-*O*-methyl-β-cyclodextrin (**2**)

6^A-Azido-6^A-deoxy-hexakis-$6^{B,C,D,E,F,G}$-*O*-*tert*-butyldimethylsilyl-β-cyclodextrin (**1**, 3.5 g, 1.9 mmol) together with methyltriphenylphosphine bromide (0.286 g, 0.8 mmol) and ground KOH (5.32 g, 95 mmol) were dissolved in THF (84 mL). The flask was set up in a magnetic stirrer and allowed to stir for 1 h. Then methyl iodide (6.55 mL, 46.2 mmol) was added dropwise. After 2 h, an ice bath was applied to the system, and a portion of NaH (60% suspension in oil, 2.12 g, 53.2 mmol) was added slowly to the reaction mixture. After 1 h, the ice bath was removed, and the reaction continued at room temperature overnight. A portion of methanol (2 mL) was added to quench the reaction, and then all solids were filtered off. The filtrate was dried and moved to a column (50 g silica gel). An elution started with a hexane/ethyl acetate 4/1 mixture and switched to a 5/2 mixture when TLC indicated the presence of the product. The extraction from the column gave 3.3 g of the product (85% yield).

^{1}H-NMR (400 MHz, $CDCl_3$) δ=5.10-5.24 (H-C^1, 7H); 4.07-4.19 (H-$C^{6B,C,D,E,F,G}$, 6H); 3.47-3.88 (H-C^3,H-C^4; H-C^5, Me-O-C^2; Me-O-C^3, H-C^{6A}); 3.05-3.20 (H-C^2, 7H); 0.91 (*t*-butyl-Si, 54H); 0.01 (methyl-Si, 36H; ^{13}C-NMR (100 MHz, $CDCl_3$) δ=98.31-99.21 (C^1); 77.15-82.78 (C^2, C^3, C^4); 71.00, 72.43 (C^5); 61.86-63.00 ($C^{6B,C,D,E,F,G}$); 61.55 (Me-O-C^2); 58.63 (MeO-C^3); 51.88 (C^{6A}); 68.00 (t-Bu-Si); -5.36 (Me-Si). HRMS (ESI): *m/z* calcd for $C_{91}H_{179}N_3O_{34}Si_6+Na^+$: 2063.10 [M+$Na^+$] found: 2063.10. [α]$CHCl_3$= +108,3° (the data are consistent with the literature [5]).

Fig. S7 ^{1}H NMR spectrum of **2** in $CDCl_3$ at 25 °C.

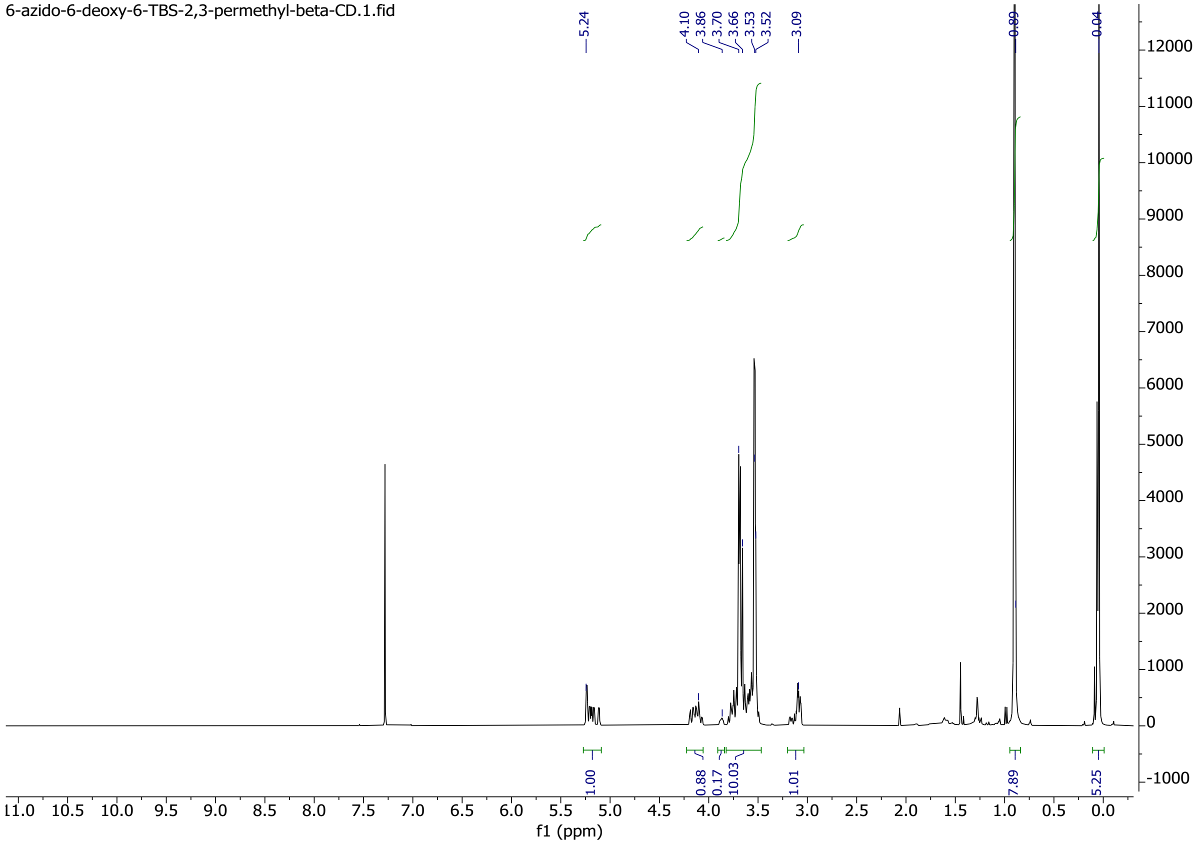

Fig. S8 DEPT-^{13}C NMR spectrum of **2** in $CDCl_3$ at 25 °C.

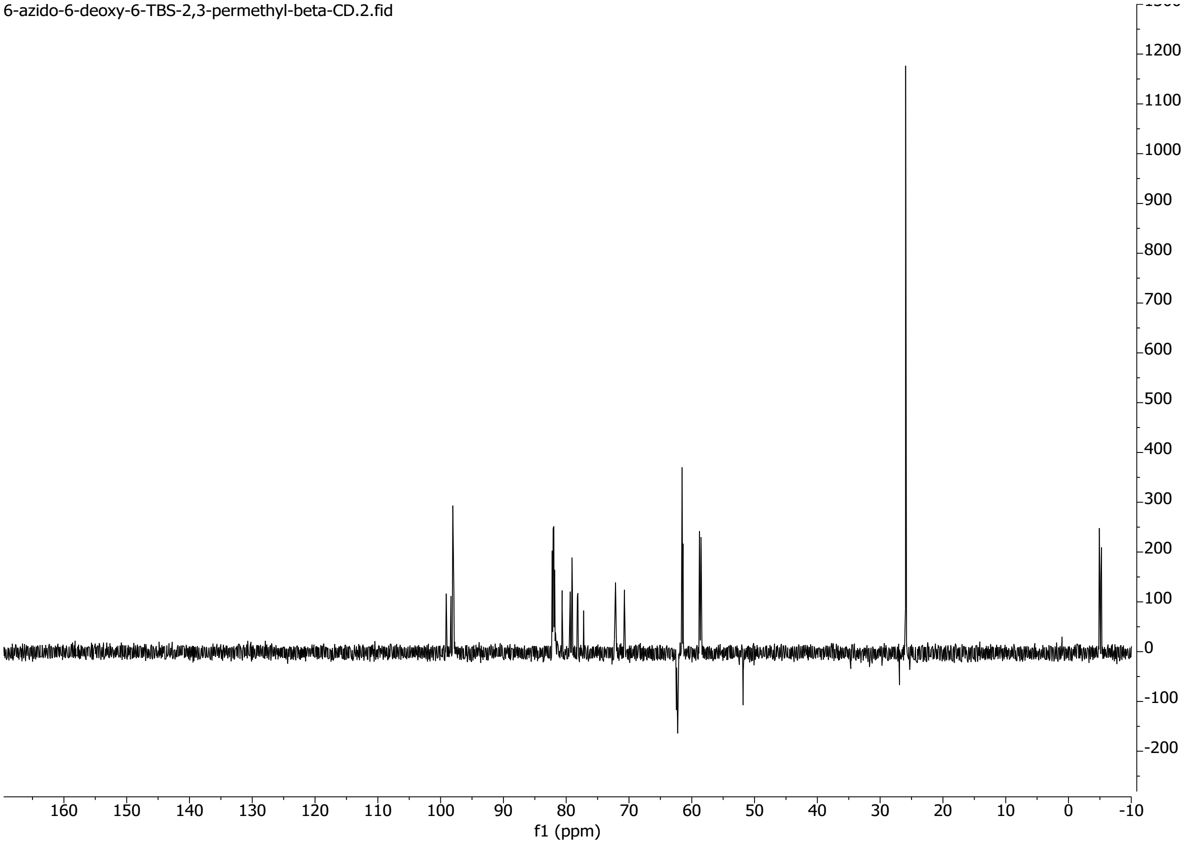

Fig. S9 COSY spectrum of **2** in $CDCl_3$ at 25 °C.

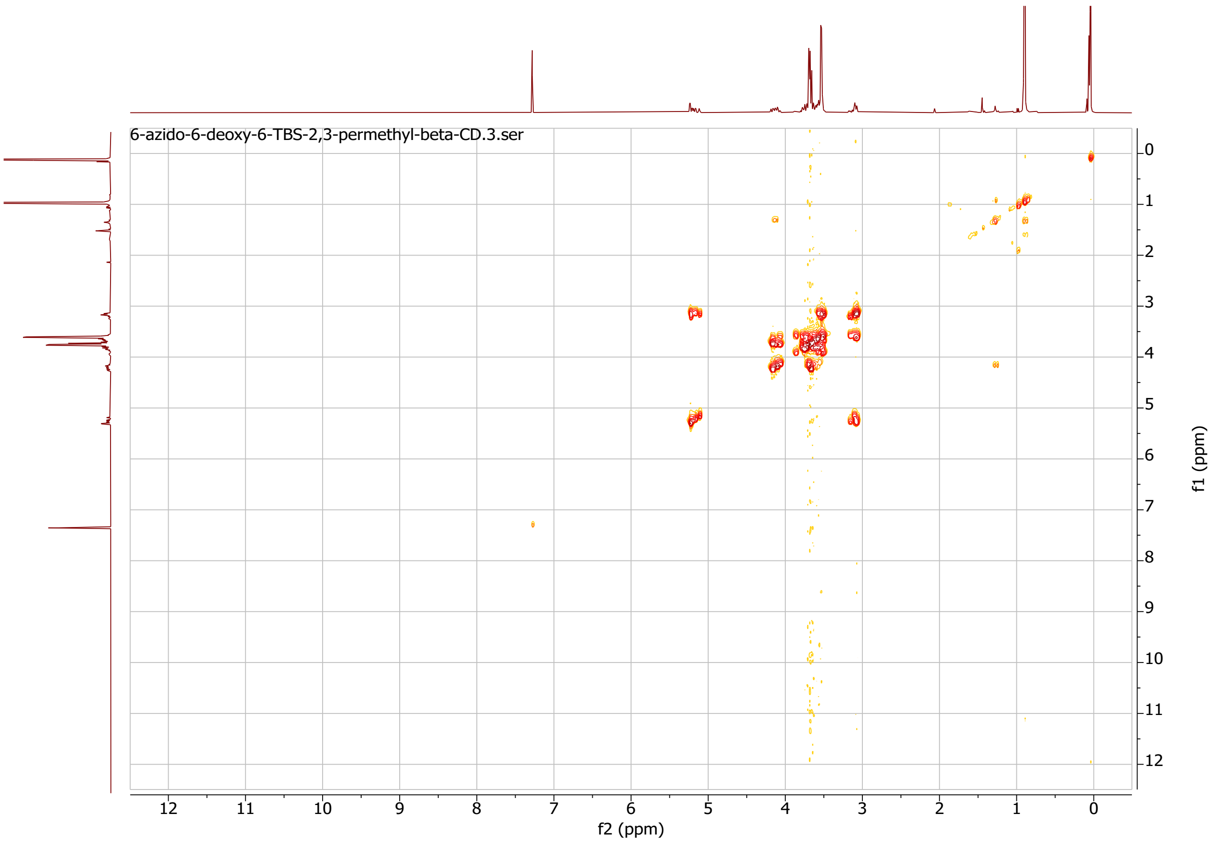

Fig. S10 HSQC spectrum of **2** in $CDCl_3$ at 25 °C.

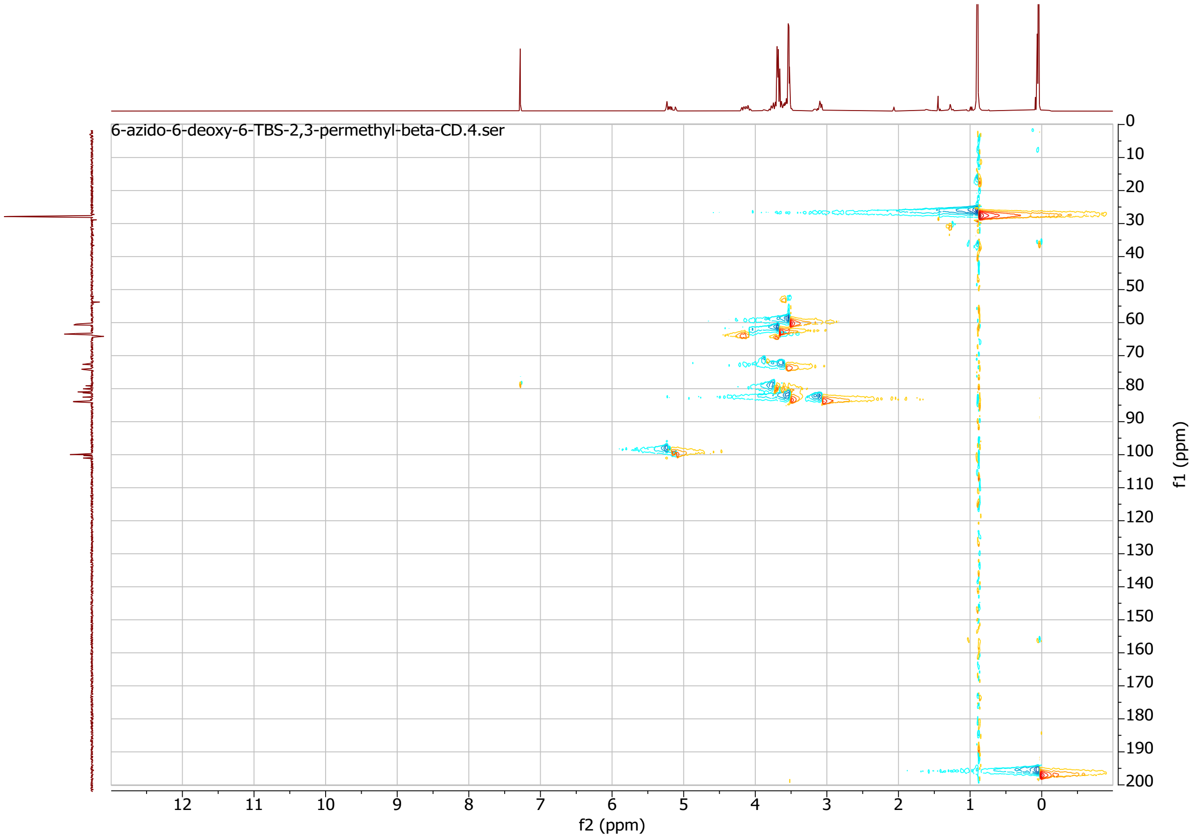

Fig. S11 IR spectrum of **2** in KBr pellet.

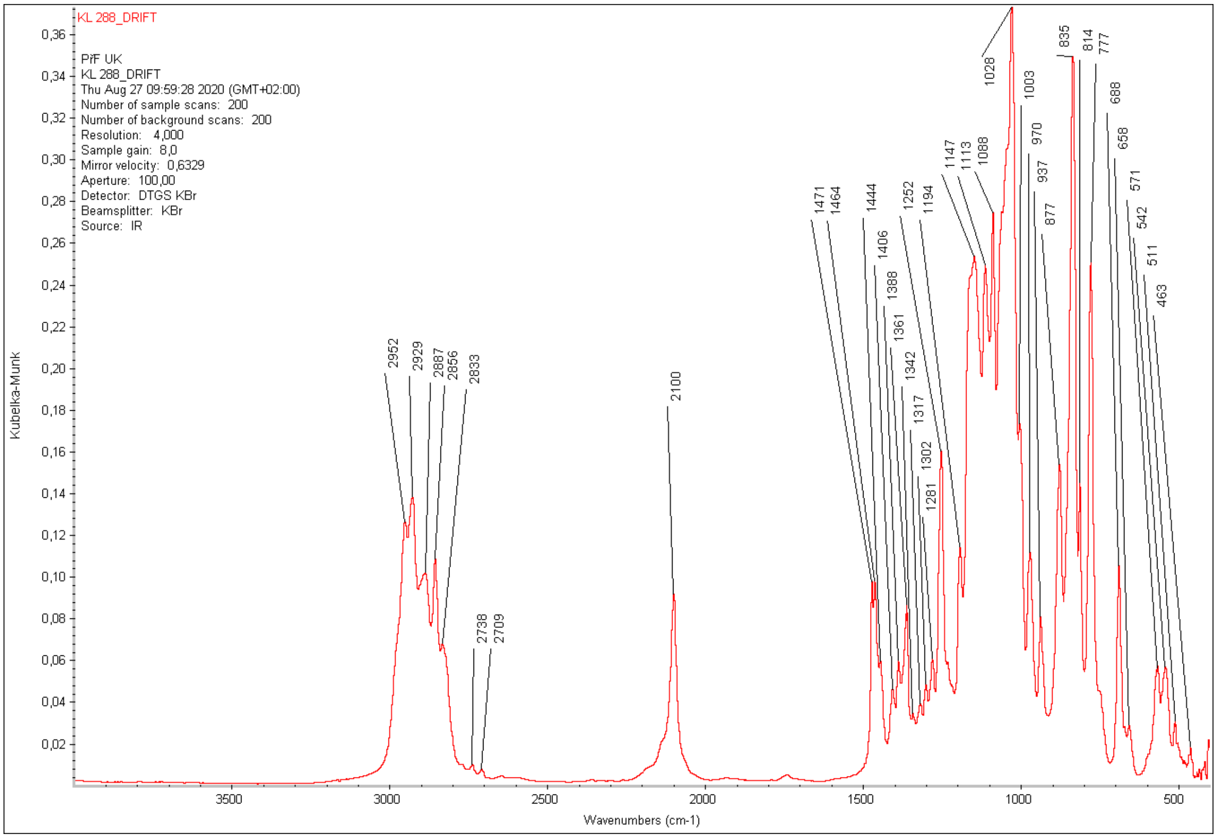

Fig. S12 UV–vis spectrum of **2** in $CHCl_3$.

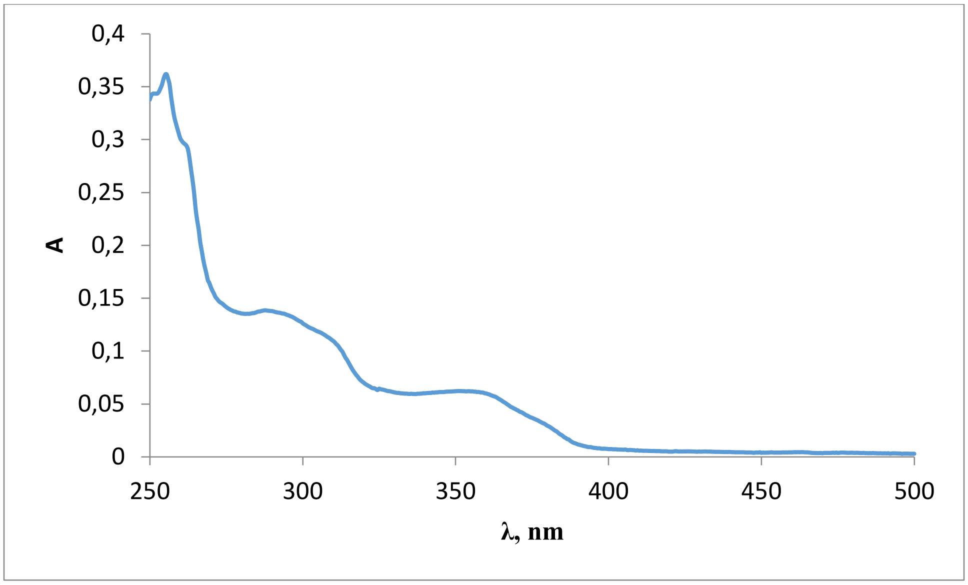

6^{A}-Azido-6^{A}-deoxy-per-2,3-*O*-methyl-β-cyclodextrin (**3**)

6^{A}-Azido-6^{A}-deoxy-hexakis-$6^{B,C,D,E,F,G}$-*O*-*tert*-butyldimethylsilyl-per-2,3-*O*-methyl-β-cyclodextrin (**2**, 1.4 g, 0.686 mmol)) together with NH_4F (1.37 g, 37 mmol) were dissolved in methanol (40 mL) in a small flask. The flask with the mixture was set in a magnetic stirrer and refluxed overnight. After, the compound was dried, dissolved in chloroform and moved to a silica gel column (50 g). The mixture of chloroform/methanol 8/1 was used to wash out the main product (0.81 g; 87% yield) from the column.

^{1}H-NMR (400 MHz, $CDCl_3$) δ=5.0-5.21 (H-C^{1}, 7H); 4.3-4.64 (HO-C^{6}, 6H); 3.25-4.01 (H-C^{6}, H-C^{5}, Me-O-C^{2}, H-C^{4}, Me-O-C^{3}, H-C^{3}); 3.15-3.23 (H-C^{2}, 7H). ^{13}C-NMR (100 MHz, $CDCl_3$) δ=98.23-99.41 (C^{1}); 78.93-82.36 (C^{2}, C^{3}, C^{4}); 71.00-72.48 (C^{5}); 61.75, 61.41, 61.22 ($C^{6B,C,D,E,F,G}$); 60.00-61.55 (Me-O-C^{3}); 58.31-59.19 (Me-O-C^{2}); 51.54 (C^{6A}). HRMS (ESI): *m/z* calcd for $C_{55}H_{97}N_3O_{34}+H^{+}$: 1356.6 [$M+H^{+}$] found: 1356.60078. [α]$CHCl_3$= +140.8° (the data are consistent with the literature [5]).

Fig. S13 ^1H NMR spectrum of **3** in $CDCl_3$ at 25 °C.

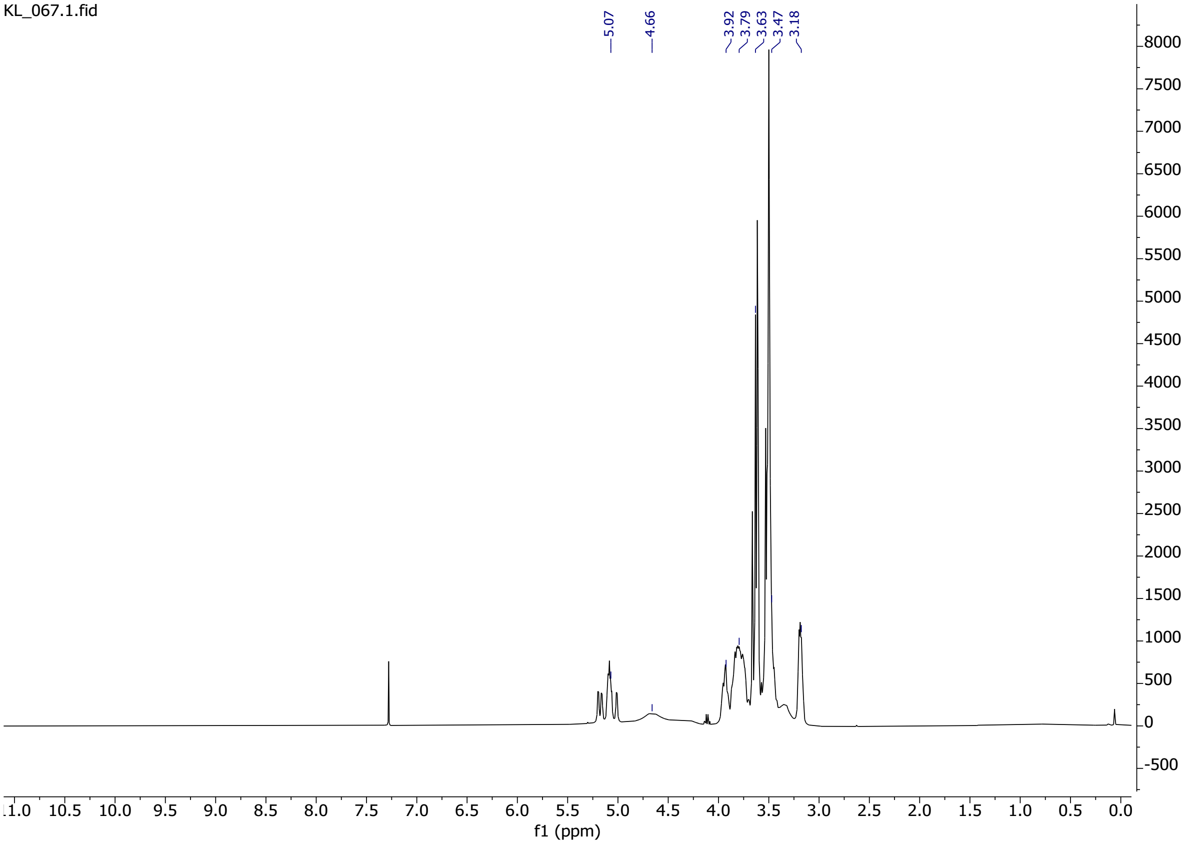

Fig. S14 DEPT-^{13}C NMR spectrum of **3** in $CDCl_3$ at 25 °C.

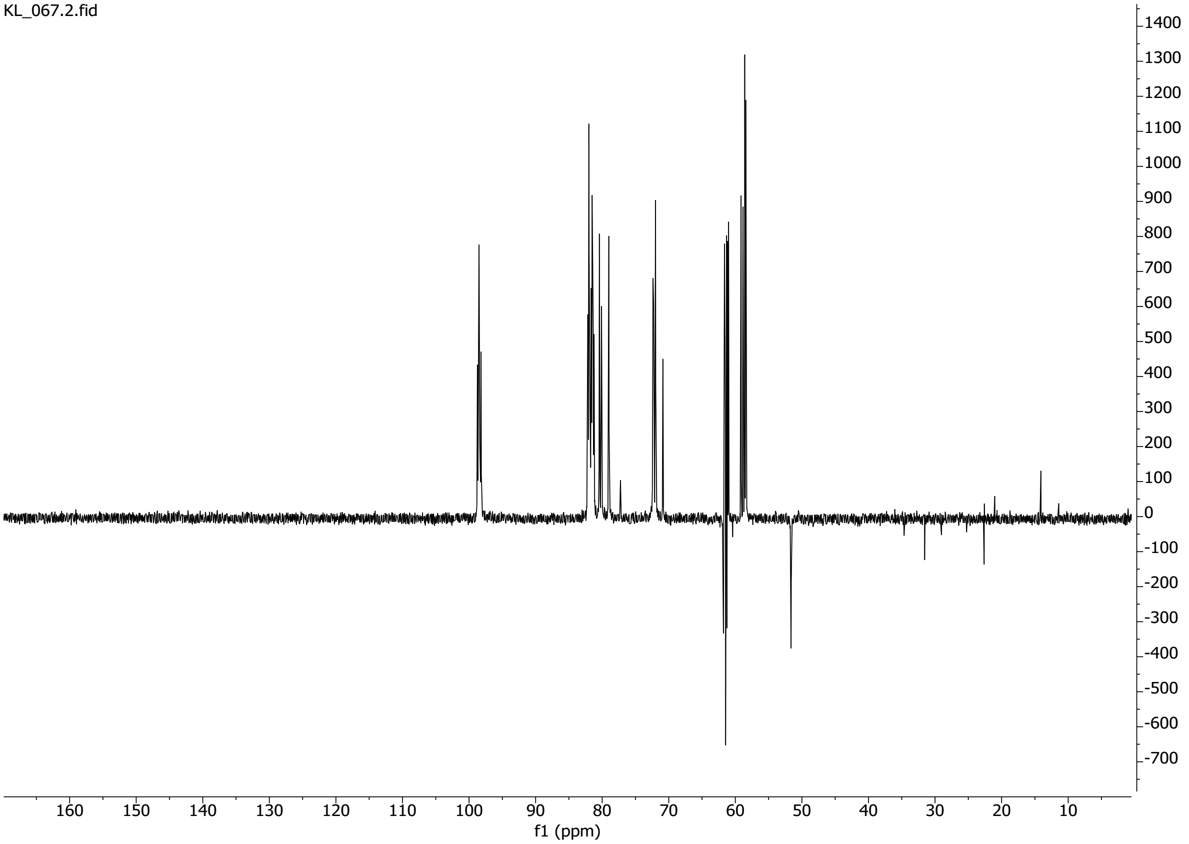

Fig. S15 COSY spectrum of **3** in $CDCl_3$ at 25 °C.

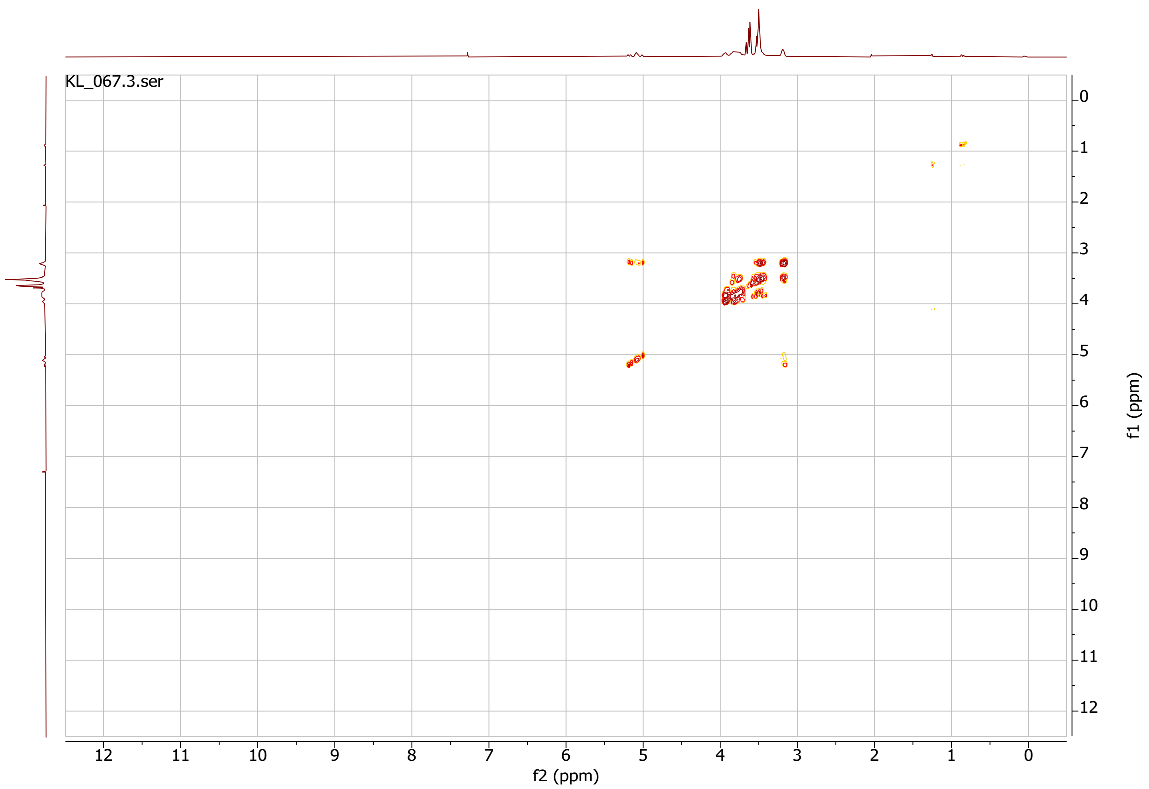

Fig. S16 HSQC spectrum of **3** in $CDCl_3$ at 25 °C.

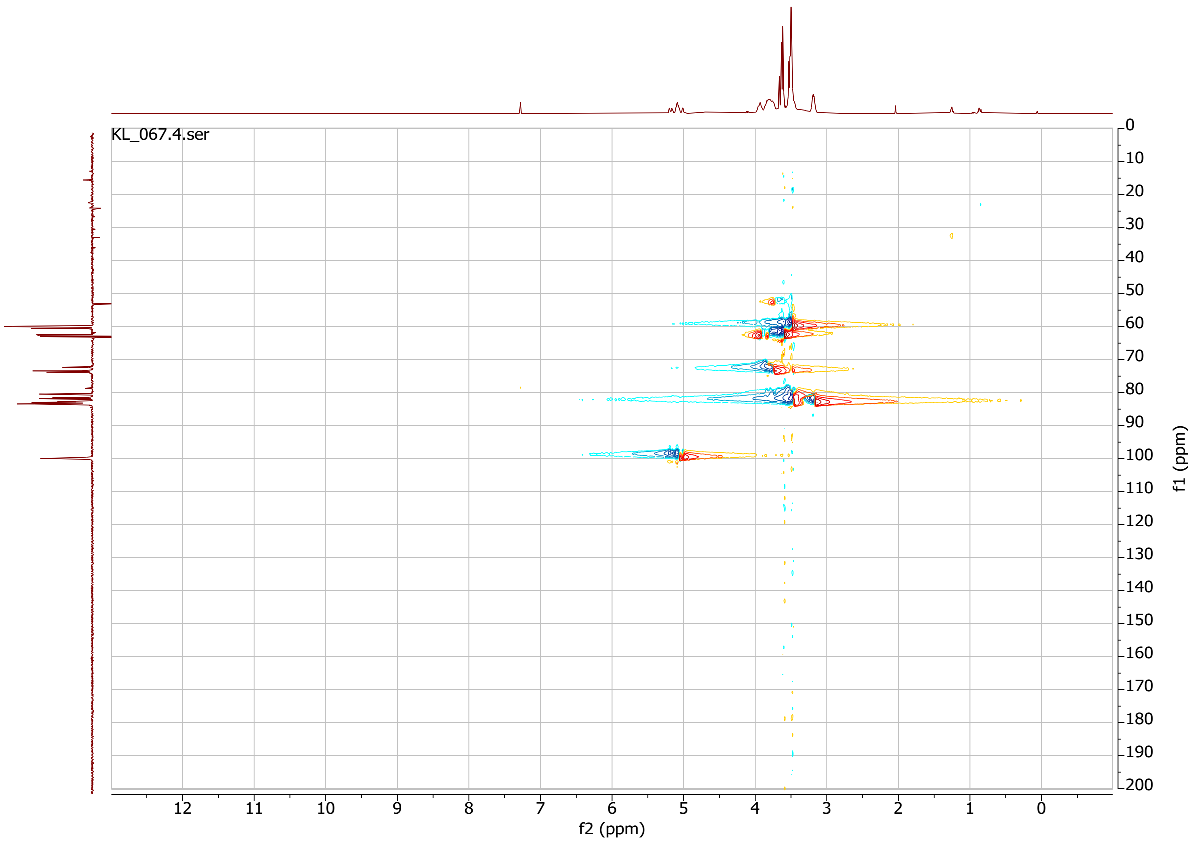

Fig. S17 IR spectrum of **3** in KBr pellet.

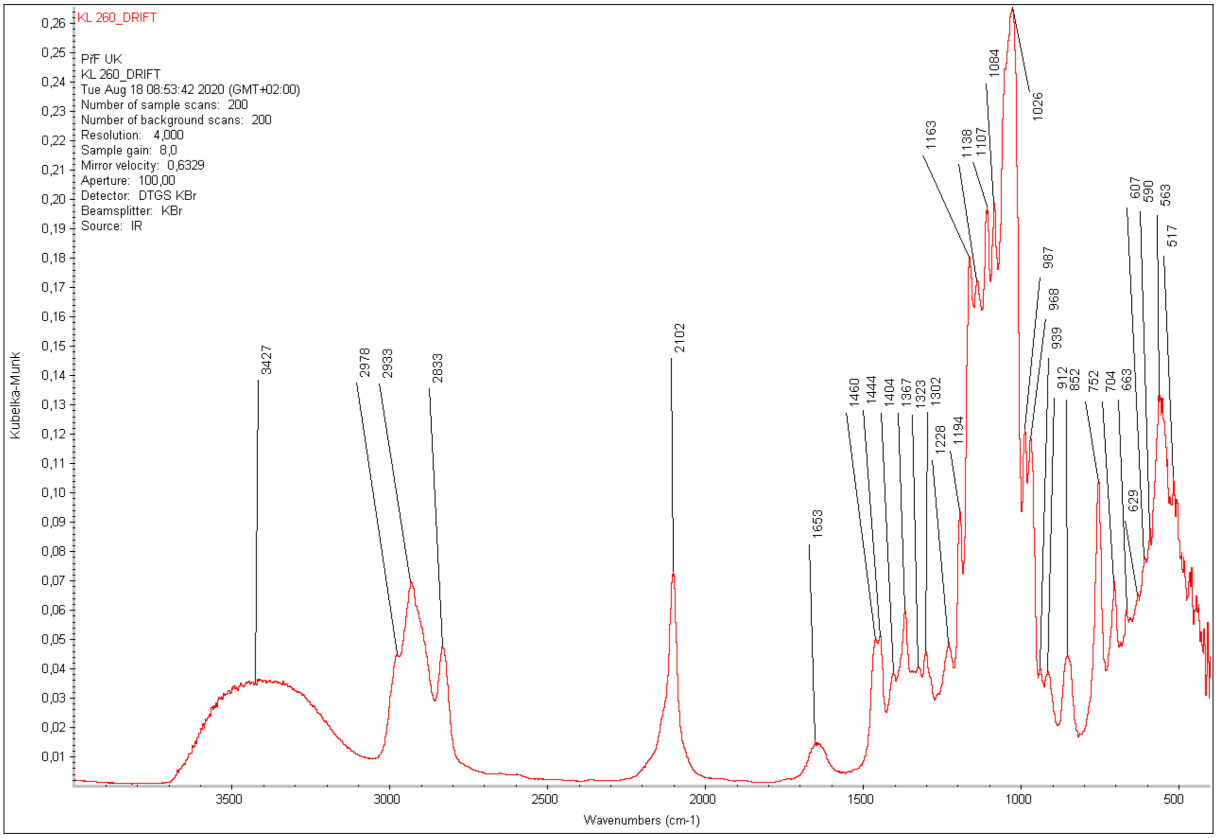

Fig. S18 UV–vis spectrum of **3** in $CHCl_3$.

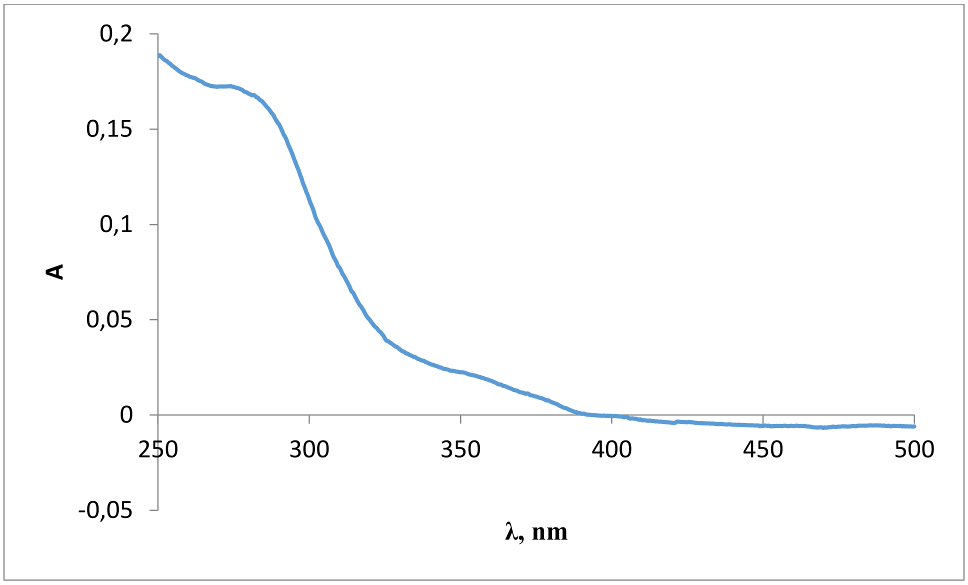

Bis-1,3-[1-(6^A-deoxy-2,3-per-*O*-methyl-β-cyclodextrin-6^A-*C*-yl)-1,2,3-triazol-4-yl]methoxymethylene (**4**)

6^A-Azido-6^A-deoxy-per-2,3-*O*-methyl-β-cyclodextrin (**3**, 1.00 g; 0.7377 mmol) was dissolved in DMF (25 mL), and the solution was bubbled by argon for 30 minutes. Then, portions of 0.5 M propargyl ether solution in DMF (0.664 mL; 0.332 mmol), copper sulfate hydrate (0.184 g; 0.7377 mmol), and sodium ascorbate (0.142 g; 0.7377 mmol) were added to the reaction mixture, and the reaction was allowed at the room temperature overnight. The solvent was removed in vacuo at 90 °C, and a column chromatography (30 g silica gel) with acetonitrile/water/conc. aq. NH_3 12/1/1 gave 0.78 g of the desirable product (84% yield).

^{1}H-NMR (400 MHz, D_2O) δ=8.0 (s, H-triazole 2H); 5.09-5.35 (H-C^1, 14H); 5.04, 4.57 (H-C^{6A} ,4H); 4.67 (s, –CH_2-triazole, 4H); 4.20 (H-C^{5A}, 2H); 2.97-3.97 (H-$C^{6B,C,D,E,F,G}$; H-$C^{5B,C,D,E,F,G}$; Me-O-C^2; H-C^4; Me-O-C^3; H-C^3; H-C^2). ^{13}C-NMR (100 MHz, D_2O) δ=126.85 (C-triazole); 96.77-97.66 (C^1); 70.24-81.44 (C^2, C^3, C^4, C^5); 63.97, 60.53 ($C^{6B,C,D,E,F,G}$); 62.84 (–CH_2-triazole); 51.71 (C^{6A}). HRMS (ESI): *m/z* calcd for $C_{118}H_{200}N_6O_{69}+2H^+$: 1404.13 [M+2H$^+$] found: 1404.12527. [α]$CHCl_3$= +159.4°.

Fig. S19 ^{1}H-NMR spectrum of **4** in D_2O at 25 °C.

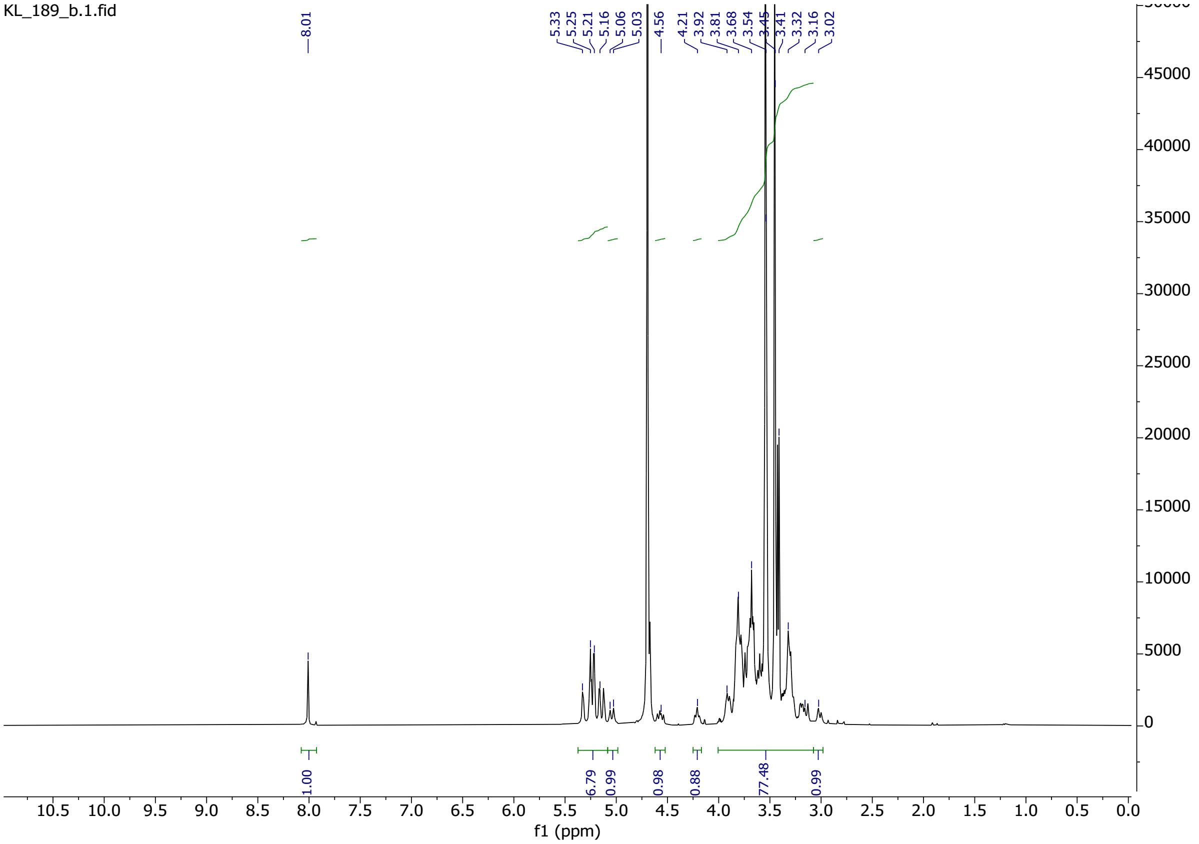

Fig. S20 DEPT-^{13}C-NMR spectrum of **4** in D_2O at 25 °C.

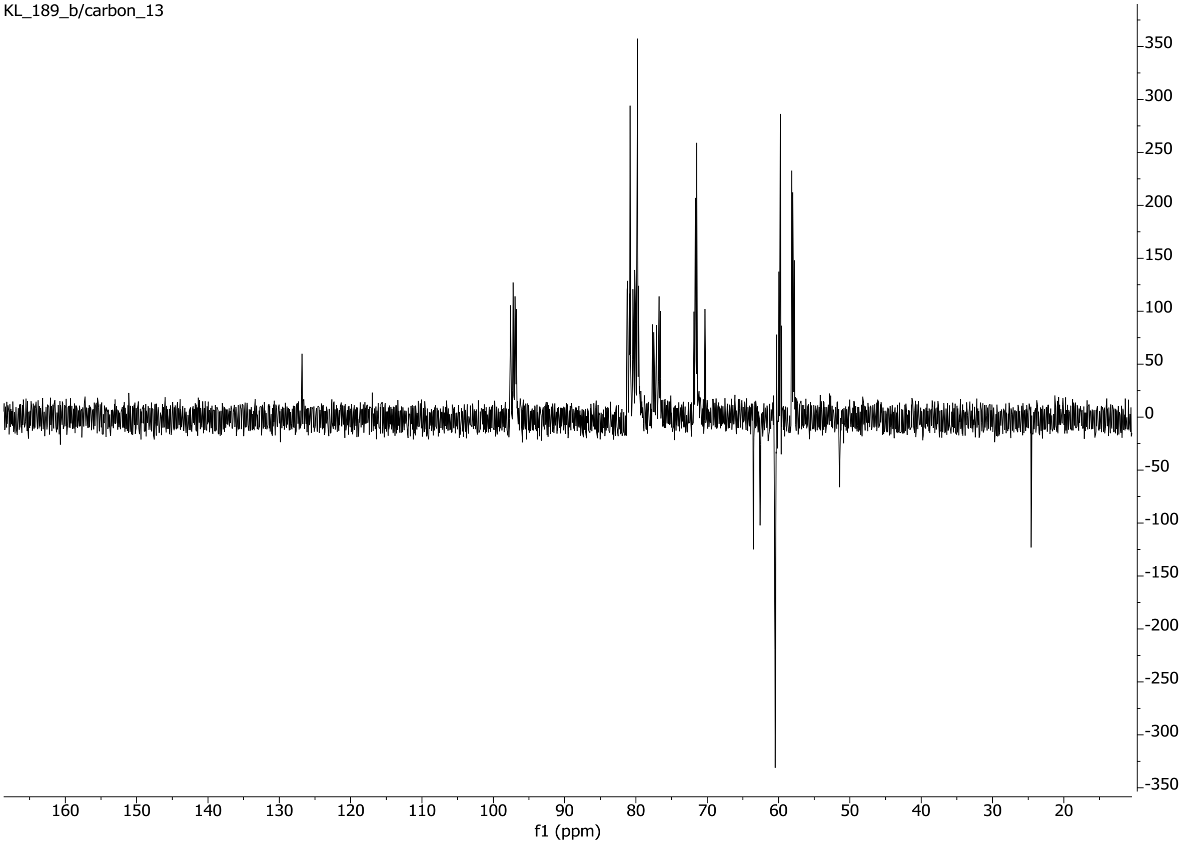

Fig. S21 COSY spectrum of **4** in D_2O at 25 °C.

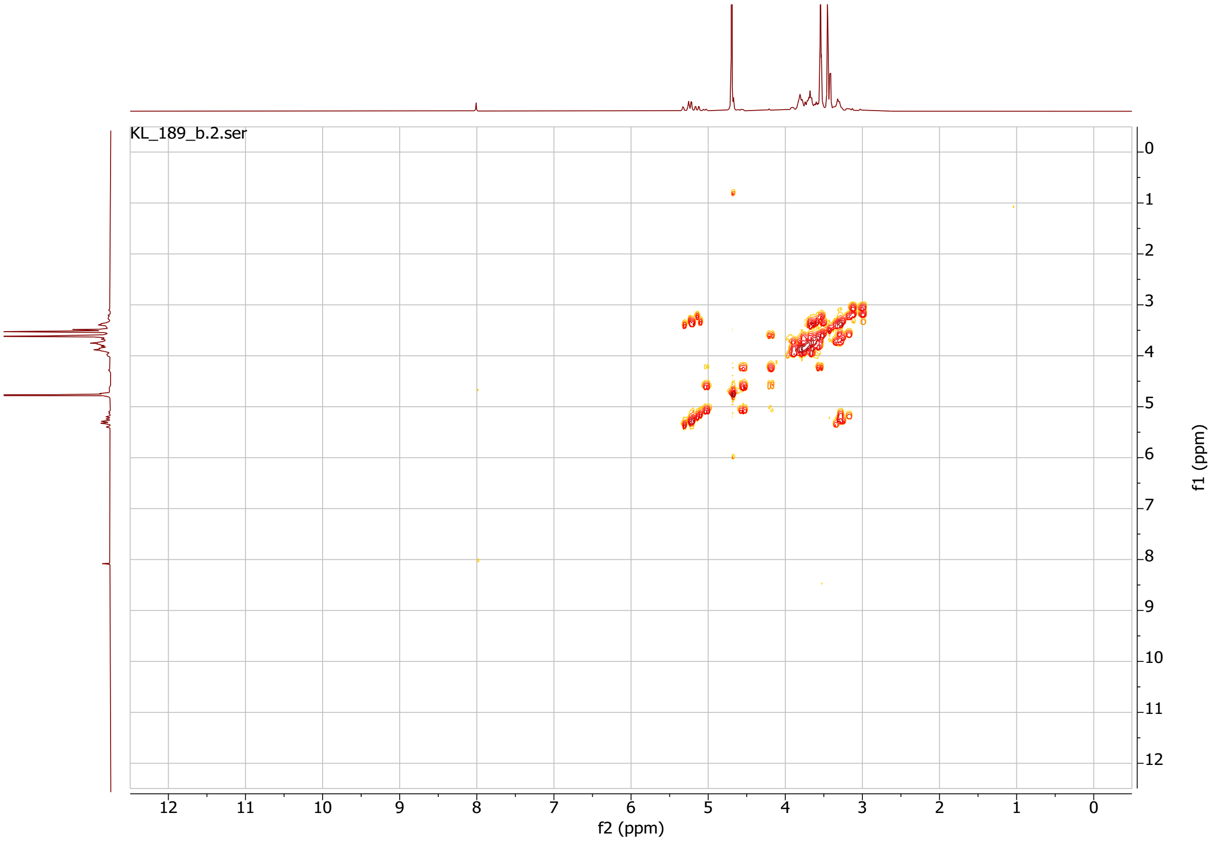

Fig. S22 HSQC spectrum of **4** in D_2O at 25 °C.

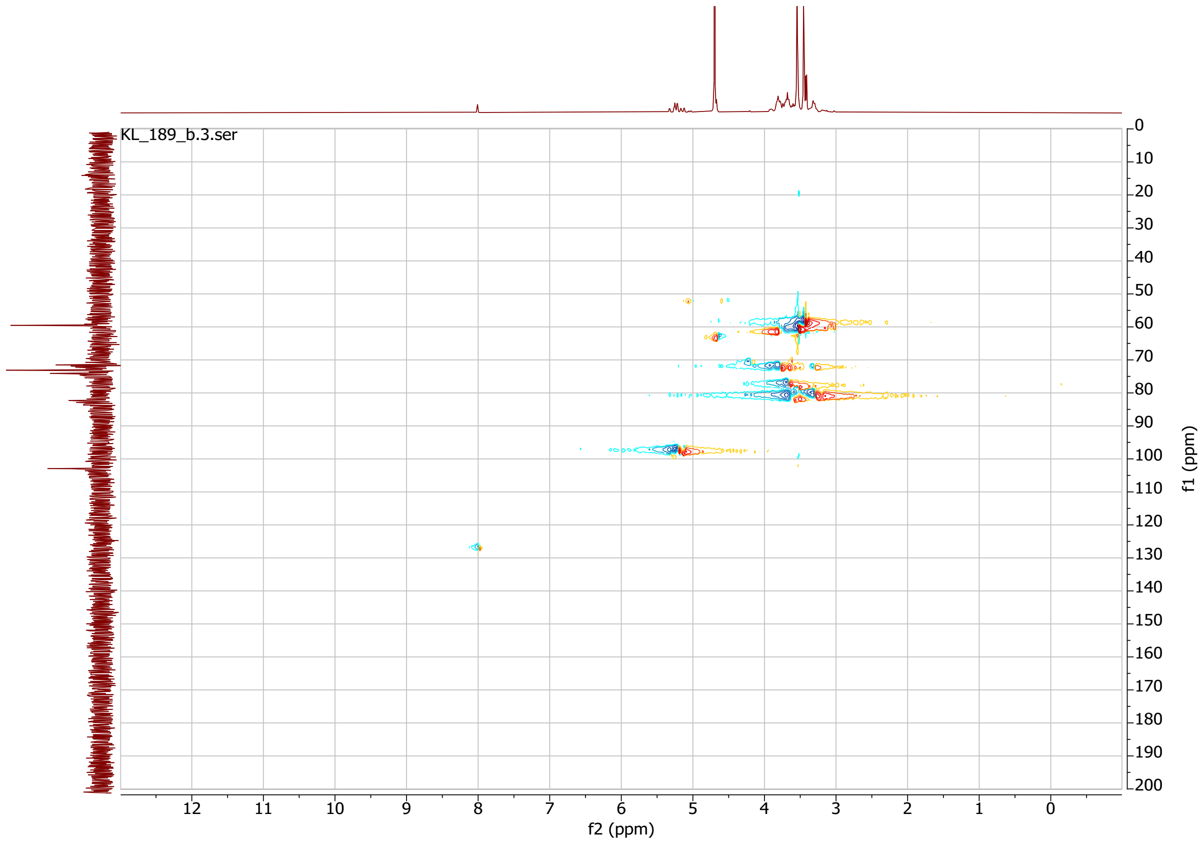

Fig. S23 NOESY spectrum of **4** in D_2O at 25 °C.

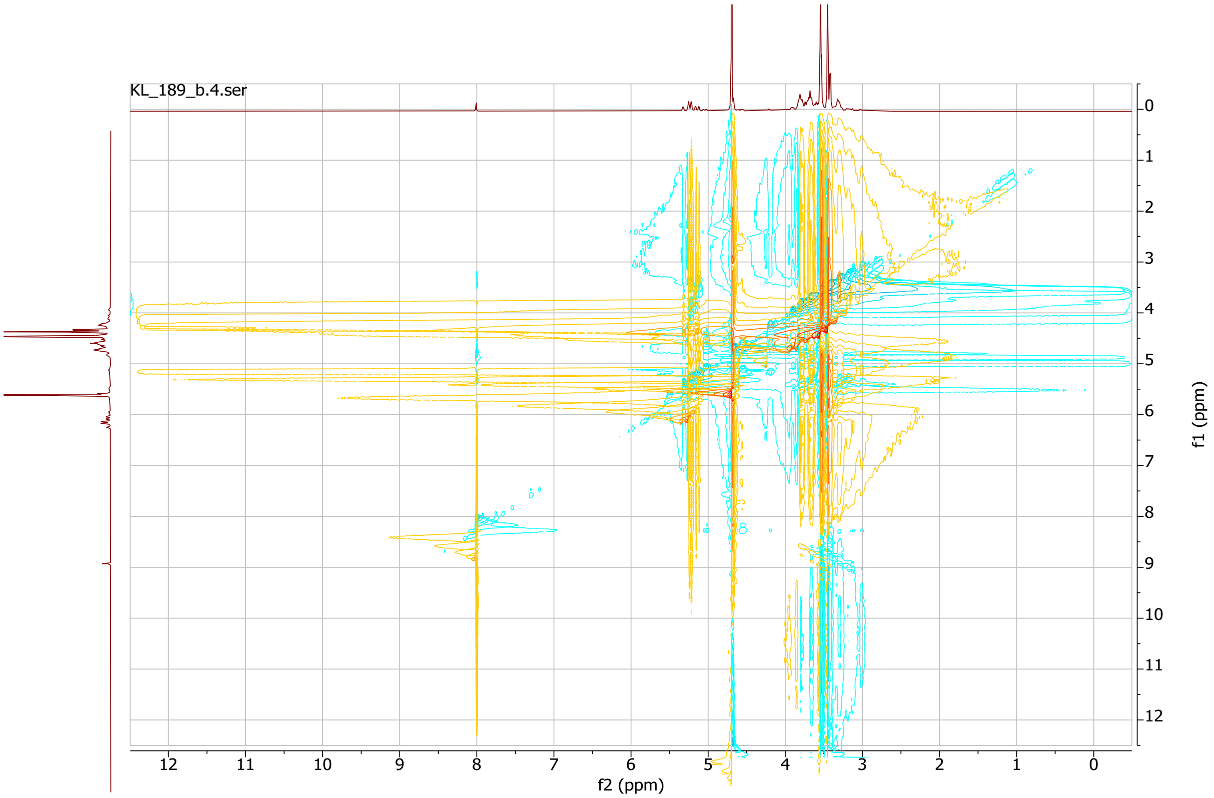

Fig. S24 IR spectrum of **4** in KBr pellet.

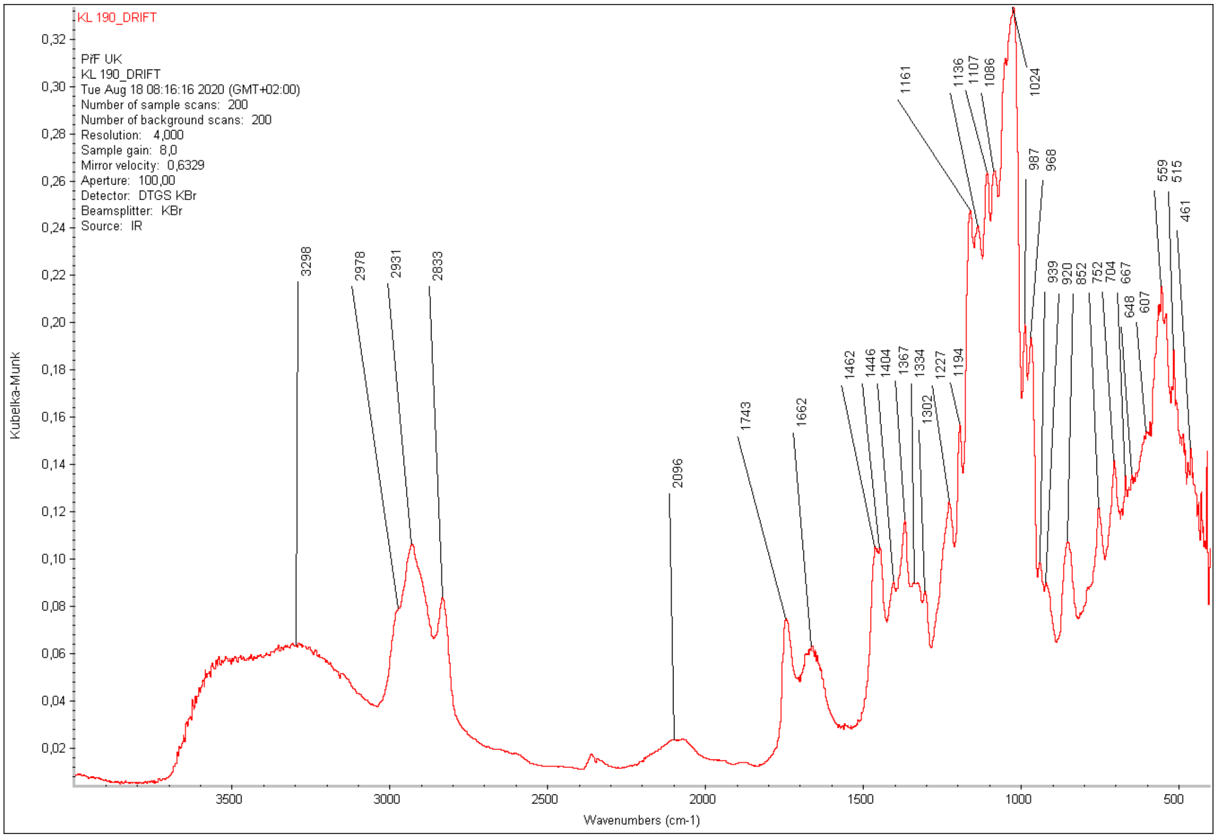

Fig. S25 UV–vis of **4** in $CHCl_3$.

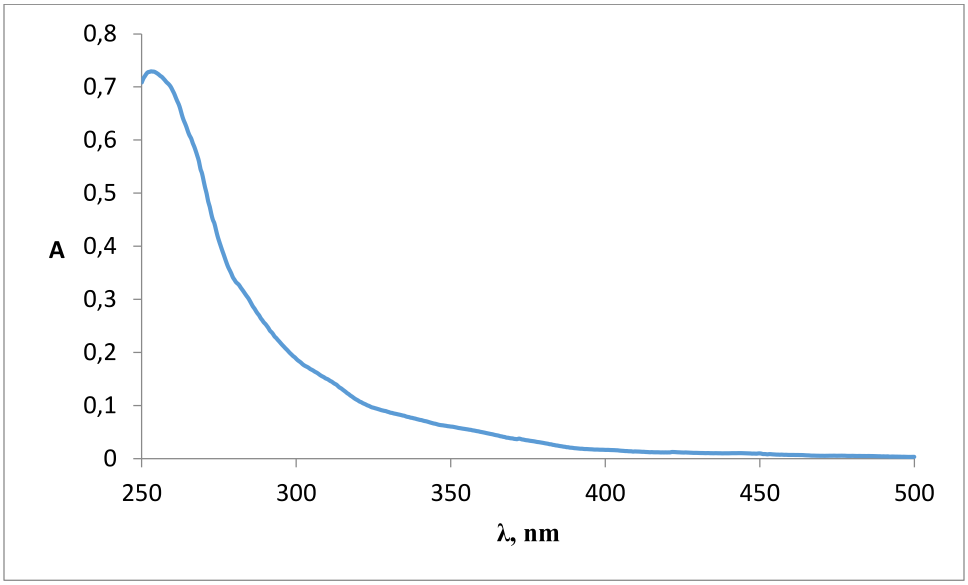

N,N'- Bis(6^A^-deoxy-2,3-per-*O*-methyl-β-cyclodextrin-6^A^-*C*-yl)urea (**5**).

6^A-Azido-6^A-deoxy-per-2,3-*O*-methyl-β-cyclodextrin (**3**, 0.91 g; 0.67 mmol) together with triphenylphosphine (0.265 g; 1.01 mmol) were dissolved in dry DMF (19 mL). The reaction mixture was bubbled with dry CO_2 overnight. Then all solvent was distilled off under vacuum, and the residue was purified by column chromatography with acetonitrile/water/conc. aq NH_3 12/1/1 (0.49 g; 54% yield).

^{1}H-NMR (400 MHz, D_2O) δ=5.21 (H-C^1, 14H); 3.22-3.88 (H-C^2, H-C^3, H-C^4, H-C^5, H-C^6); 3.53 (s, Me-O-C^2), 3.43 (s, Me-O-C^3). ^{13}C-NMR (100 MHz, D_2O) δ=97.40 (C^1); 81.14 (C^3); 77.34 (C^4); 71.76 (C^5); 60.04 (Me-O-C^2); 60.62 (C^6); 58.30 (Me-O-C^3). HRMS (ESI): *m/z* calcd for $C_{113}H_{196}N_2O_{69}$+2H$^+$: 1344.10 [M+2H$^+$] found: 1344.096; [α]$CHCl_3$= +155.4°.

Fig. S26 ^{1}H-NMR spectrum of **5** in D_2O at 25 °C.

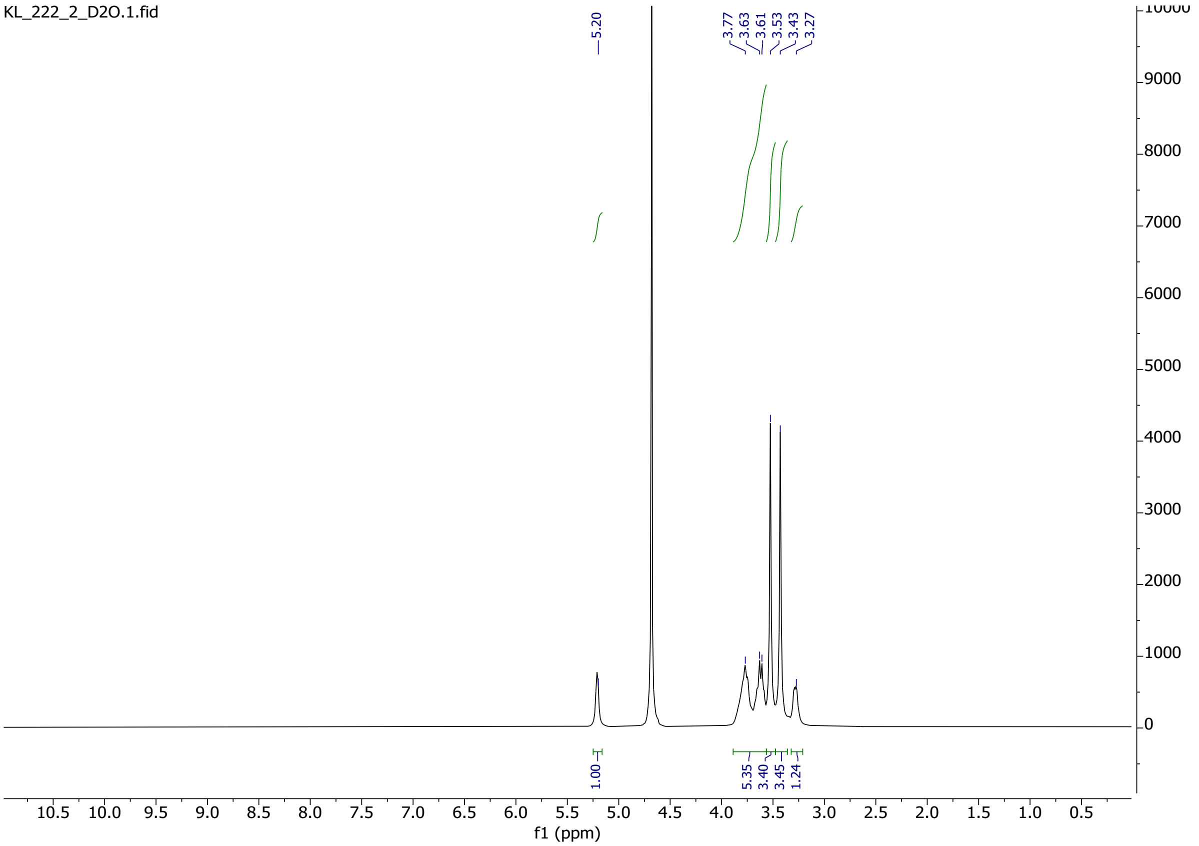

Fig. S27 DEPT-^{13}C-NMR spectrum of **5** in D_2O at 25 °C.

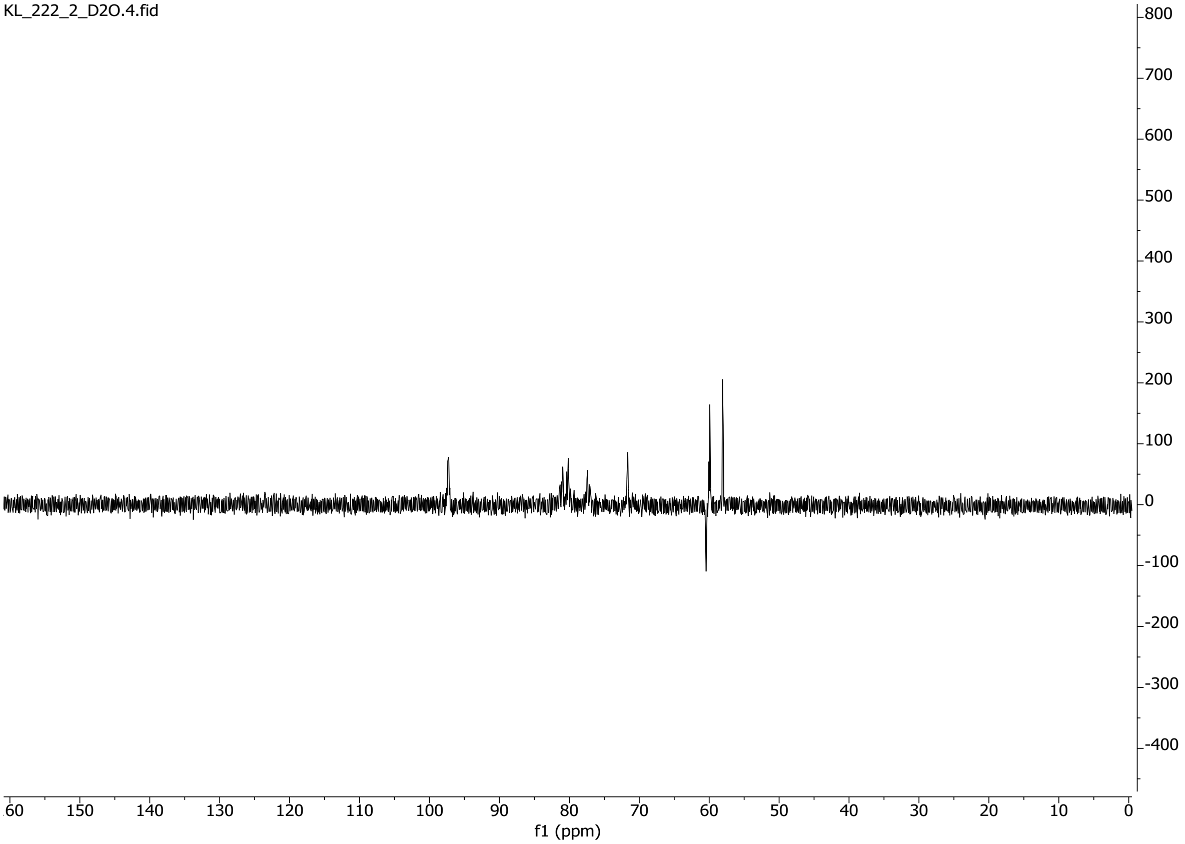

Fig. S28 COSY spectrum of **5** in D_2O at 25 °C.

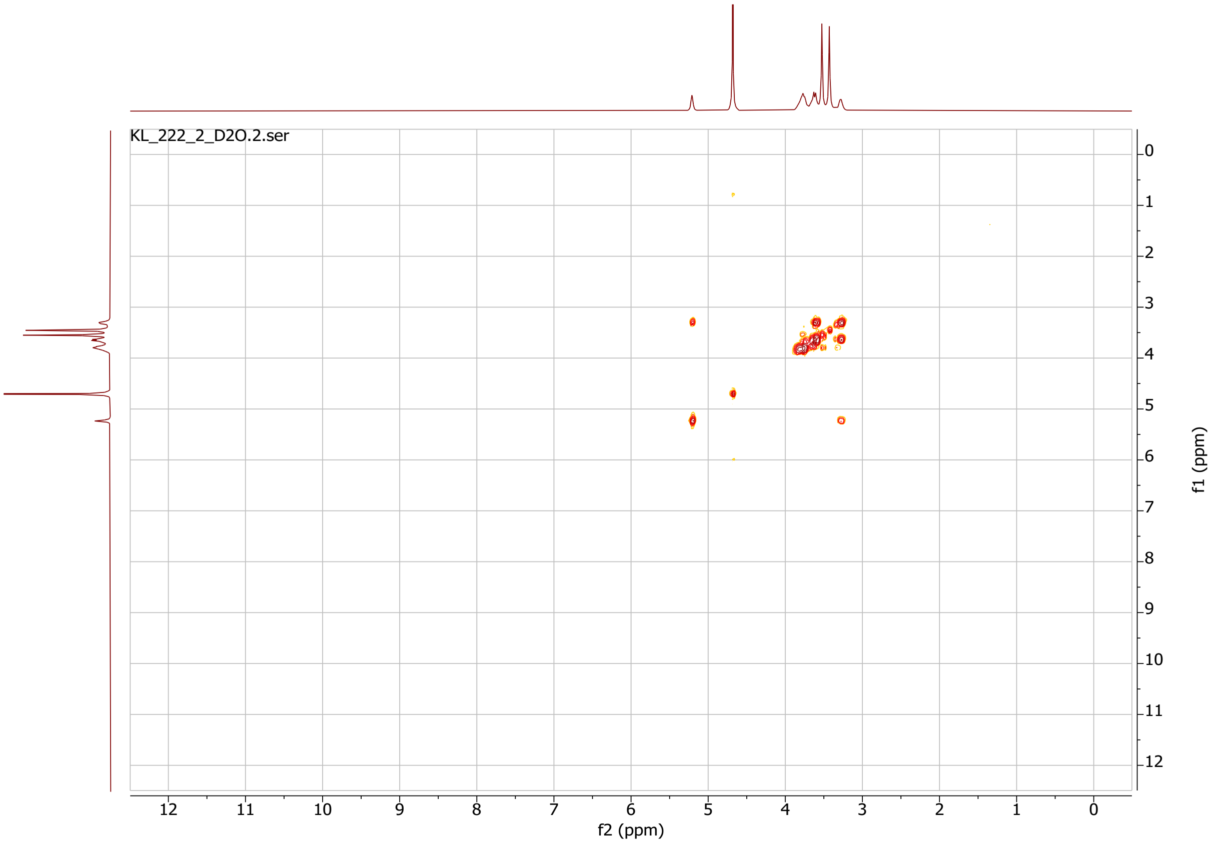

Fig. S29 HSQC spectrum of **5** in D_2O at 25 °C.

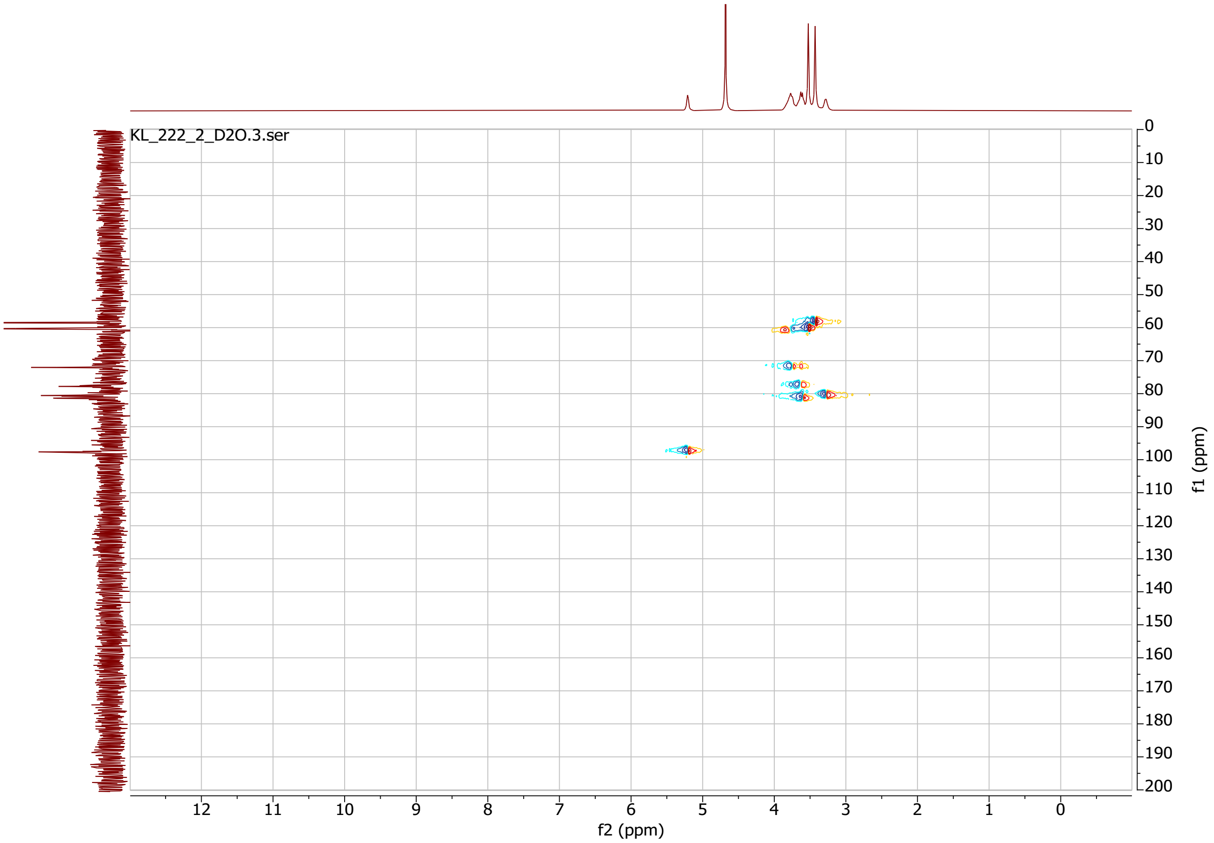

Fig. S30 IR spectrum of **5** in KBr pellet.

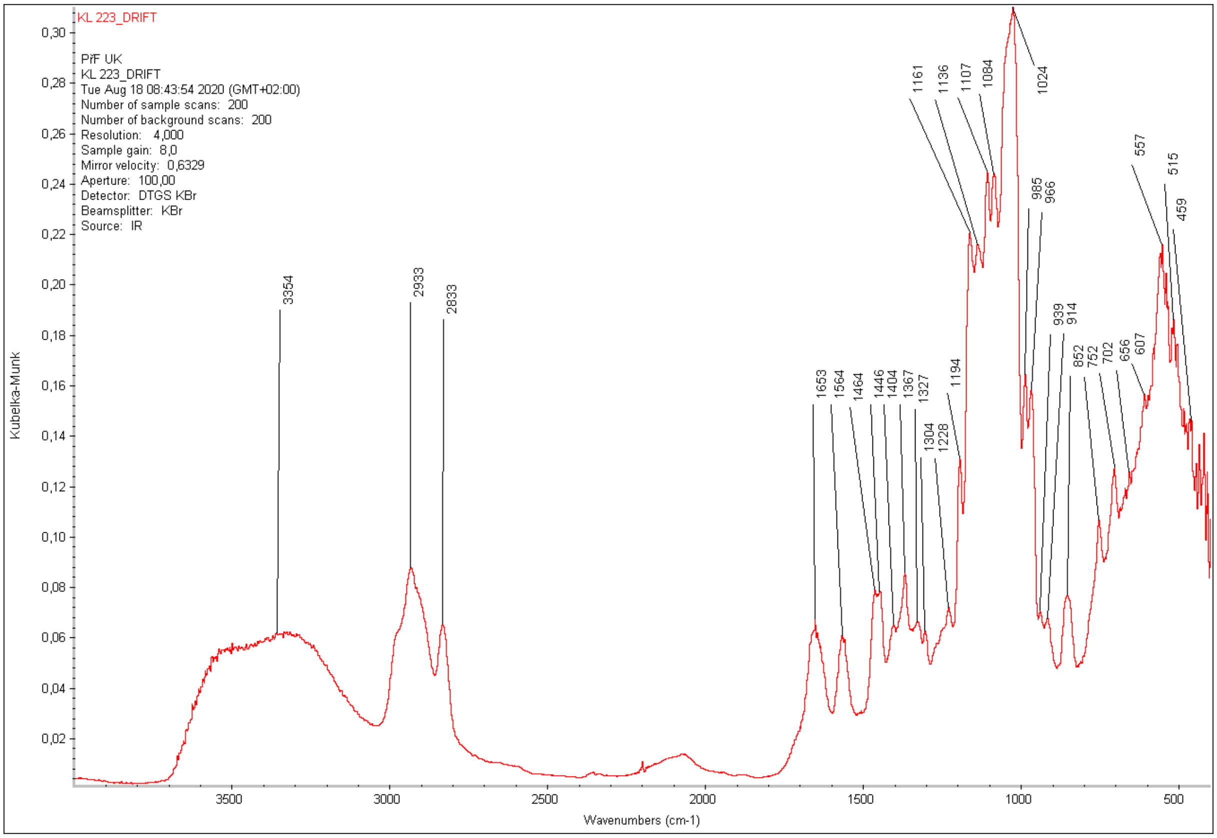

Fig. S31 UV–vis spectrum of **5** in $CHCl_3$.

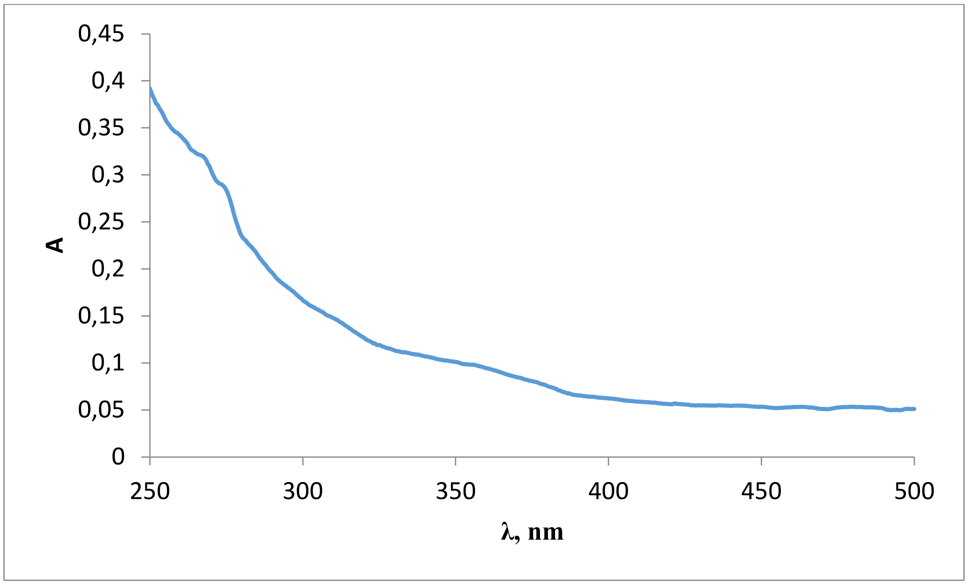

6^{A}-Azido-6^{A}-deoxy-hexakis-$6^{B,C,D,E,F,G}$-*O* -*tert*-butyl-dimethylsilyl-per-2,3-*O*-acetyl-β-cyclodextrin (**6**)

6^{A}-Azido-6^{A}-deoxy-hexakis-$6^{B,C,D,E,F,G}$-*O*-*tert*-butyldimethylsilyl-β-cyclodextrin (**1**, 1.27 g, 0.6887 mmol) was dissolved in pyridine/acetic anhydride 2/1 mixture (25 mL). The reaction mixture was allowed to stir at 80 °C overnight. After this, the reaction mixture was diluted by chloroform and washed in a dropping funnel successively with 1 M hydrochloric acid, saturated $NaHCO_3$ solution, and brine (all 50 mL). Drying of the organic part gave the pure compound (1.64 g; 97% yield).

^{1}H-NMR (400 MHz, $CDCl_3$) δ=5.27-5.45 (H-C^3, 7H); 5.11-5.21 (H-C^1, 7H), 3.50-4.27 (H-C^2; H-C^6; H-C^4; H-C^5); 2.0-2.13 (Me-C(O), 42H); 0.84-0.94 (t-Bu-Si, 54H); 0.04-0.09 (Me-Si, 36H). ^{13}C-NMR (100 MHz, $CDCl_3$) δ=95.96-97.36 (C^1); 74.9-77.12 (C^4); 70.33-72.31 (C^3, C^2, C^5); 61.42-62.18 ($C^{6B,C,D,E,F,G}$); 51.49 (C^{6A}); 25.87 (t-Bu-Si); 20.91 (Me-C(O)); -5.67 (Me-Si). HRMS (ESI): *m/z* calcd for $C_{106}H_{181}N_3O_{48}Si_6$+$Na^+$: 2455.03 [M+$Na^+$]; found: 2455.04. [α]$CHCl_3$= +90°.

Fig. S32 ^{1}H-NMR spectrum of **6** in $CDCl_3$ at 25 °C.

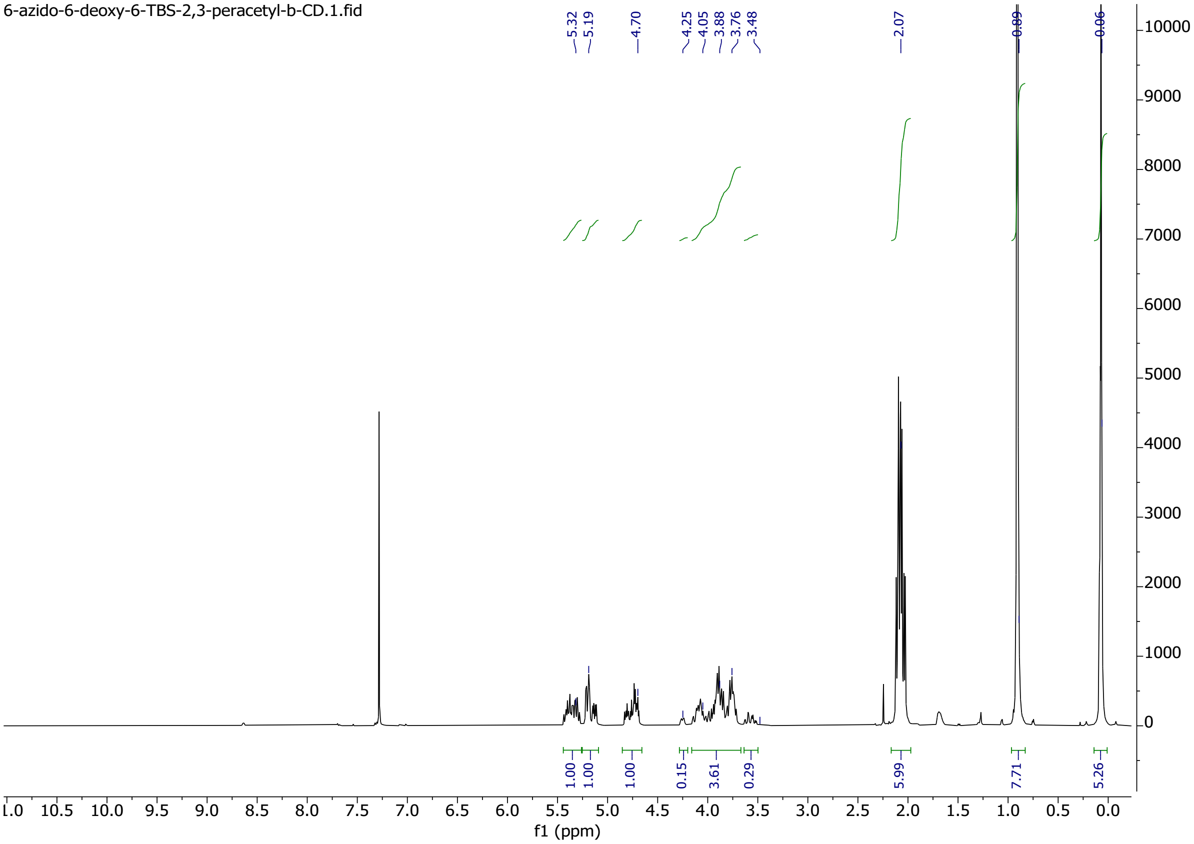

Fig. S33 DEPT-^{13}C-NMR spectrum of **6** in $CDCl_3$ at 25 °C.

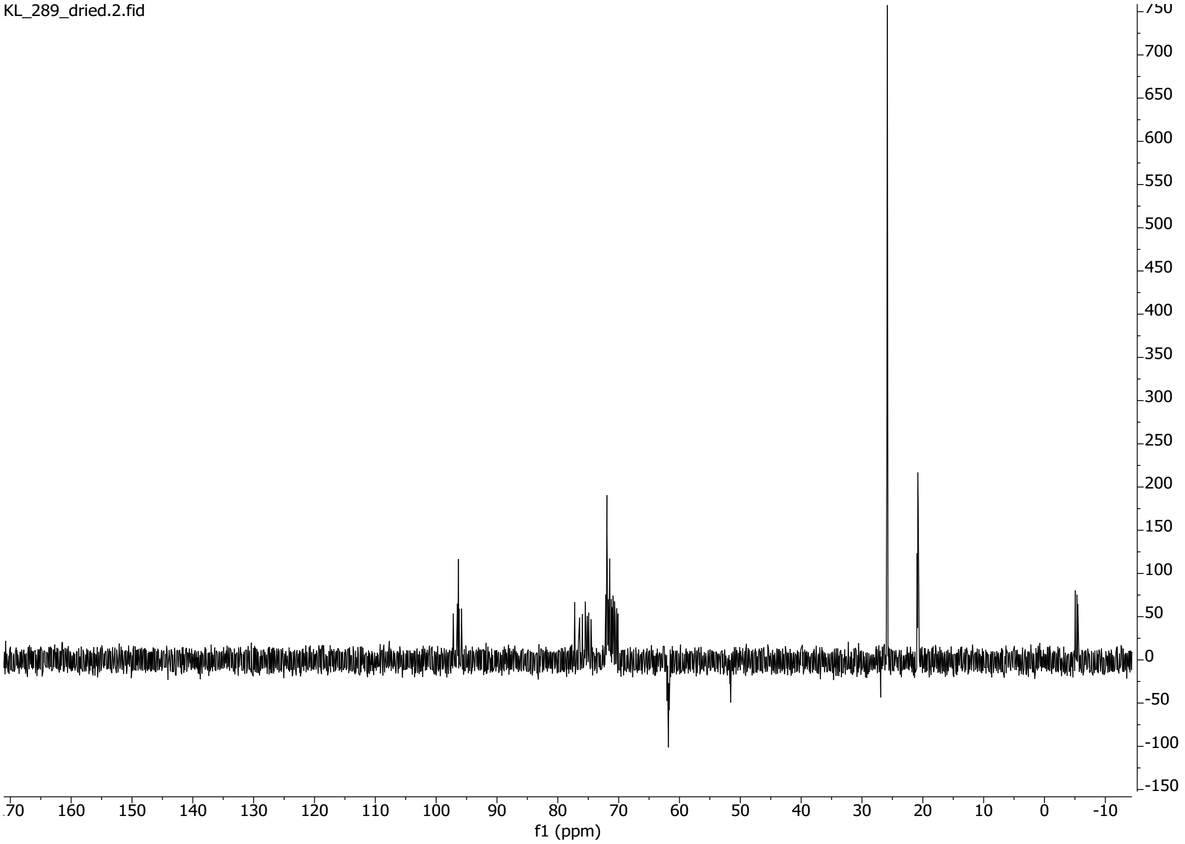

Fig. S34 COSY spectrum of **6** in $CDCl_3$ at 25 °C.

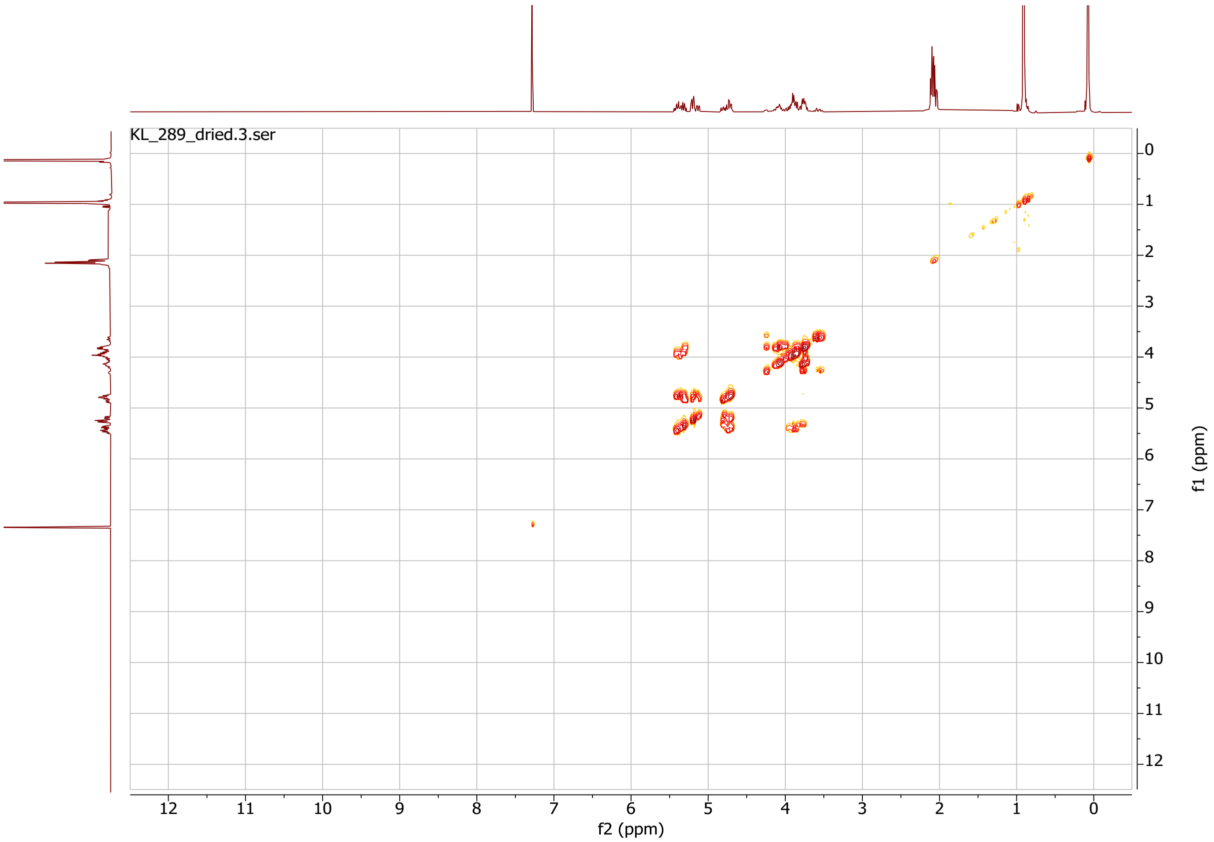

Fig. S35 HSQC spectrum of **6** in $CDCl_3$ at 25 °C.

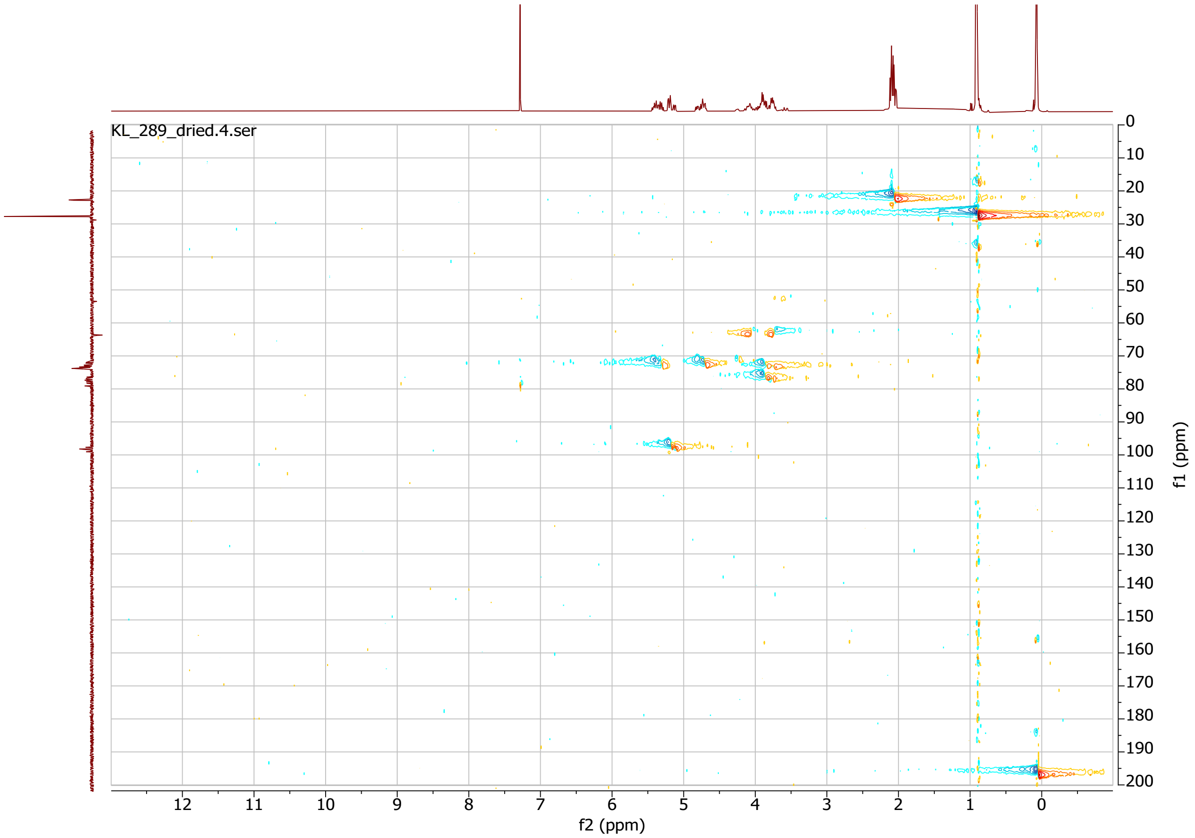

Fig. S36 IR spectrum of **6** in KBr pellet.

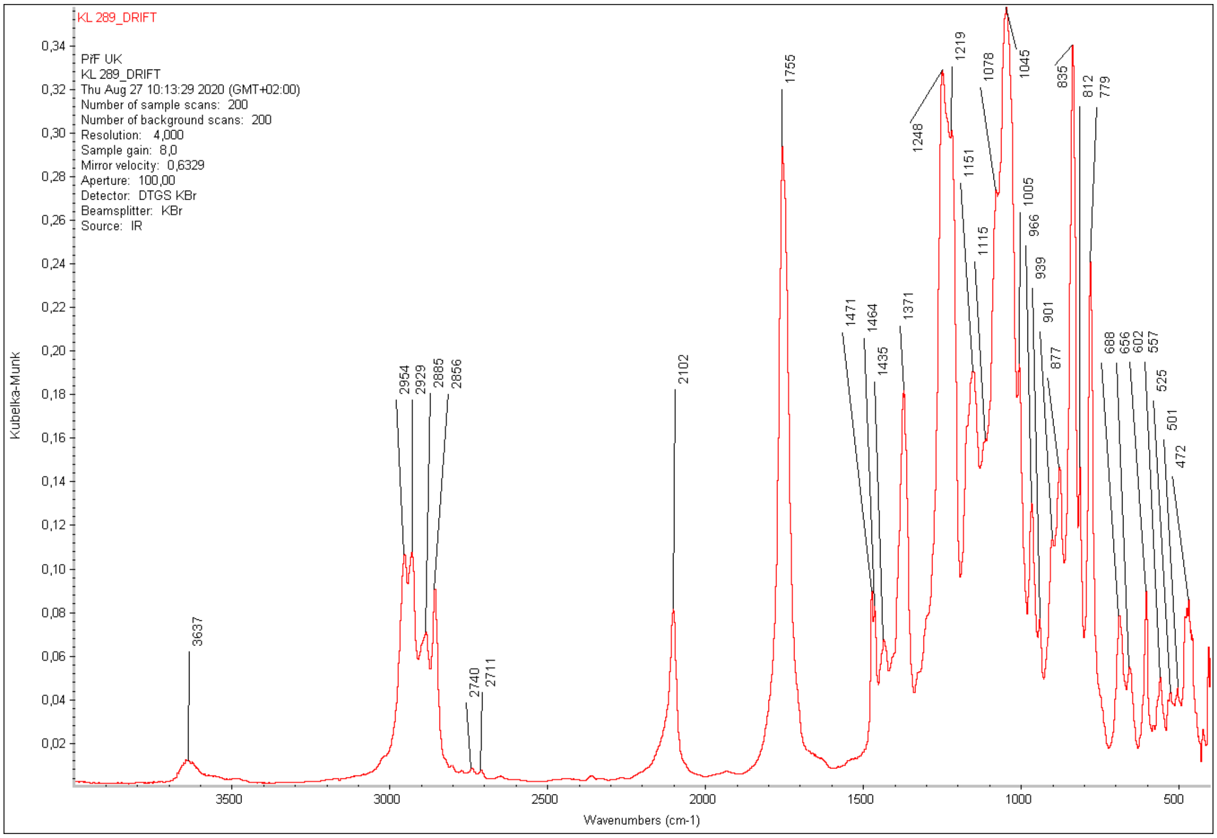

Fig. S37 UV–vis spectrum of **6** in $CHCl_3$.

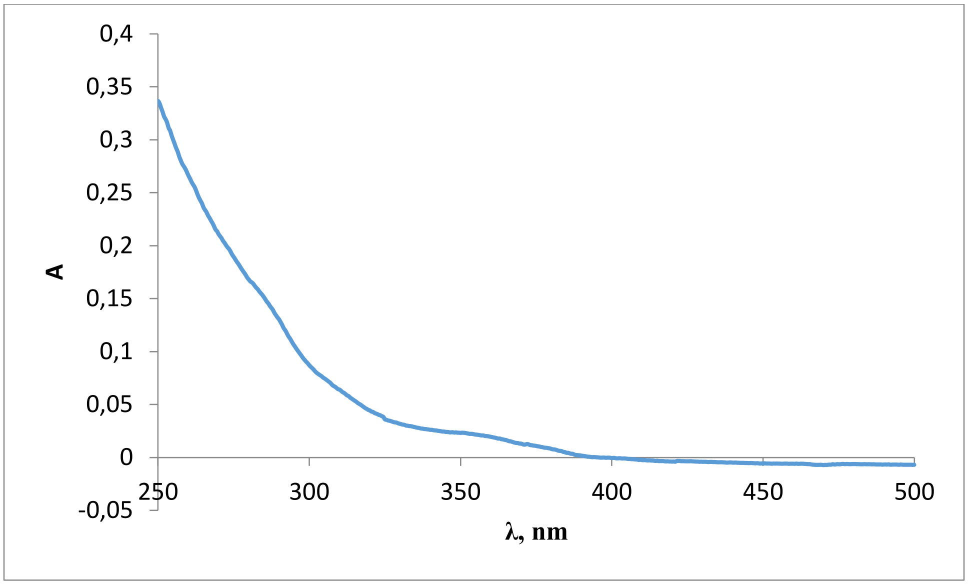

6^A-Azido-6^A-deoxy-per-2,3-*O*-acetyl-β-cyclodextrin (**7**)

6^A-Azido-6^A-deoxy-hexakis-$6^{B,C,D,E,F,G}$-*O*-*tert*-butyl-dimethylsilyl-per-2,3-*O*-acetyl-β-cyclodextrin (**6**, 1.64 g, 0.676 mmol) was dissolved in DCM (24 mL). The flask with the reaction mixture was set up in a magnetic stirrer at room temperature, and boron trifluoride etherate (1.6 mL, 12.96 mmol) was added dropwise to the reaction mixture. After 3 h, the reaction mixture was washed with ice-cold $NaHCO_3$ solution in a dropping funnel, concentrated on rotavap. The product was isolated by chromatography on a silica gel column (100 g). The main product was eluted with chloroform/methanol mixture 9/1 in the 88% yield (1.03 g).

^{1}H-NMR (400 MHz, [d_4] MeOH) δ=5.38-5.48 (H-C^3, 7H); 5.13-5.24 (H-C^1, 7H); 4,75-4,82 (H-C^2, 7H); 3.68-4.15 (H-C^4, H-C^5, H-C^6); 2.05-2.15 (Me-C(O), 42H). ^{13}C-NMR (100 MHz, [d_4] MeOH) δ=96.17-96.78 (C^1); 75.43-76.93 (C^4); 72.38 (C^5); 70.43-71.32 (C^2, C^3); 60.37 ($C^{6B,C,D,E,F,G}$); 51.25 (C^{6A}); 19.62 (Me-C(O)). HRMS (ESI): *m/z* calcd for $C_{70}H_{97}N_3O_{48}+Na^+$:1770.51 [M+$Na^+$]; found 1770.51; [α]$CHCl_3$= +118,5°.

Fig. S38 ^{1}H-NMR spectrum of **7** in [d$_4$]-MeOH at 25 °C.

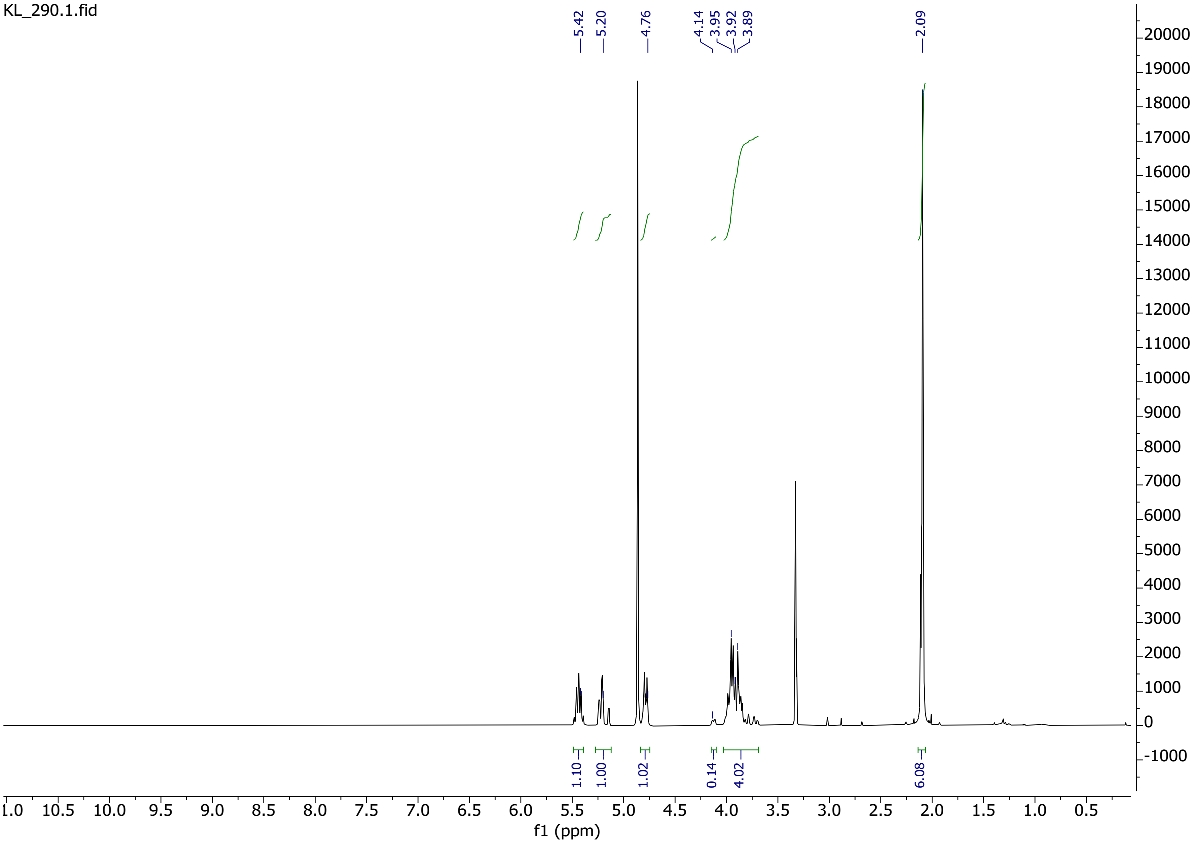

Fig. S39 DEPT-^{13}C-NMR spectrum of **7** in [d_4]-MeOH at 25 °C.

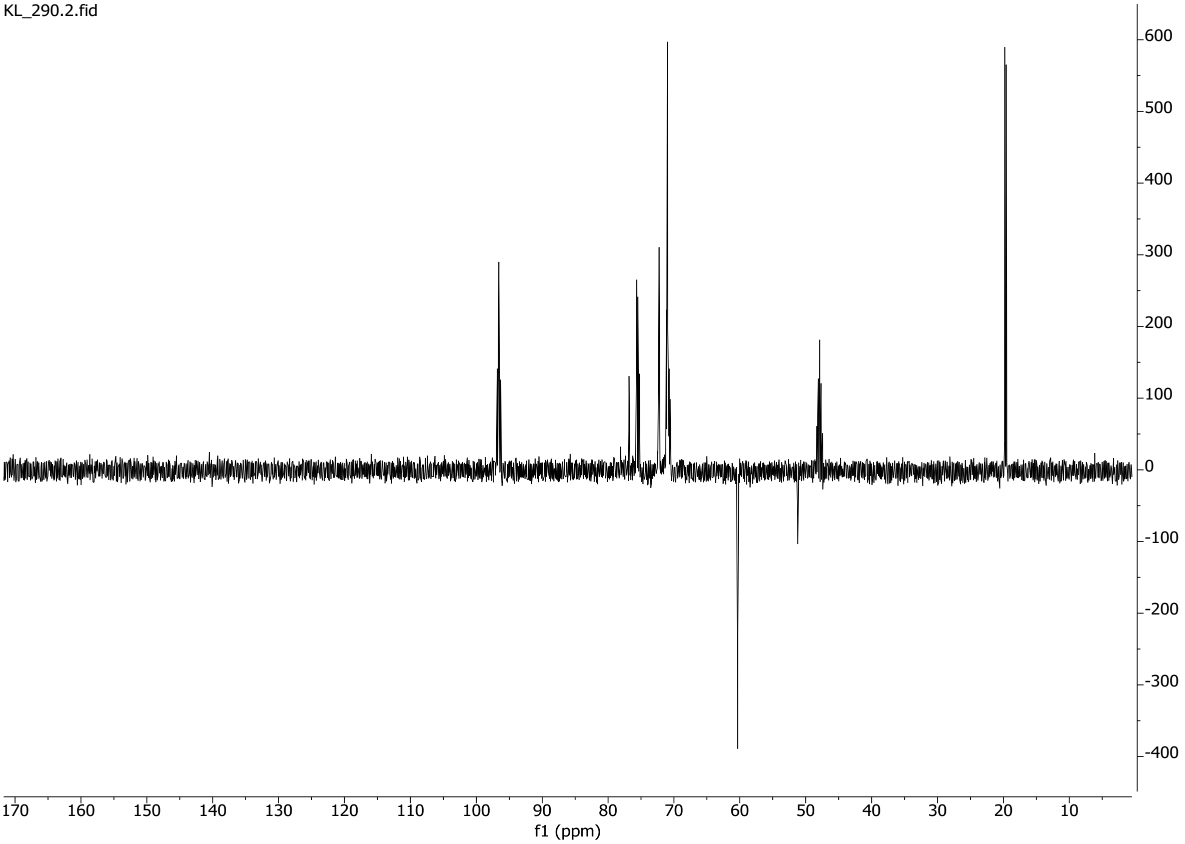

Fig. S40 COSY spectrum of **7** in [d_4]-MeOH at 25 °C.

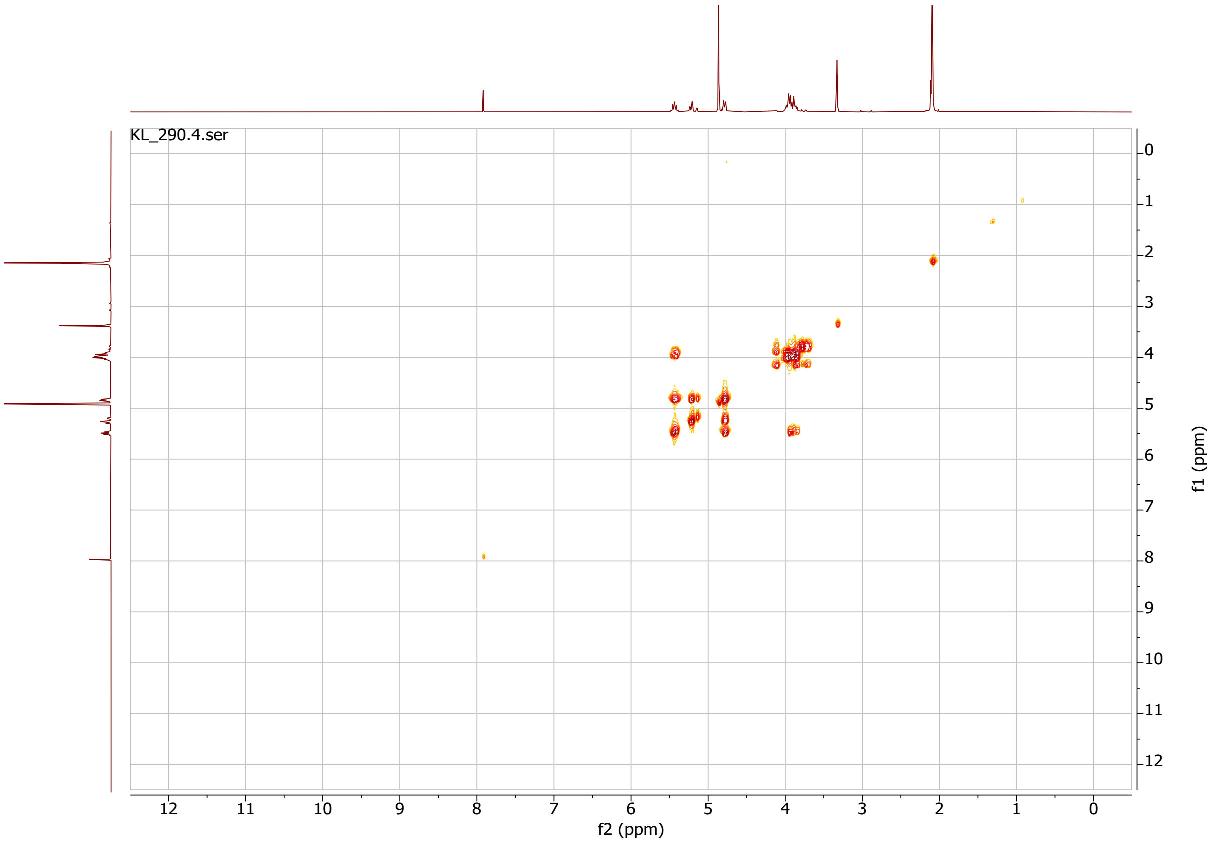

Fig. S41 HSQC spectrum of **7** in [d_4]-MeOH at 25 °C.

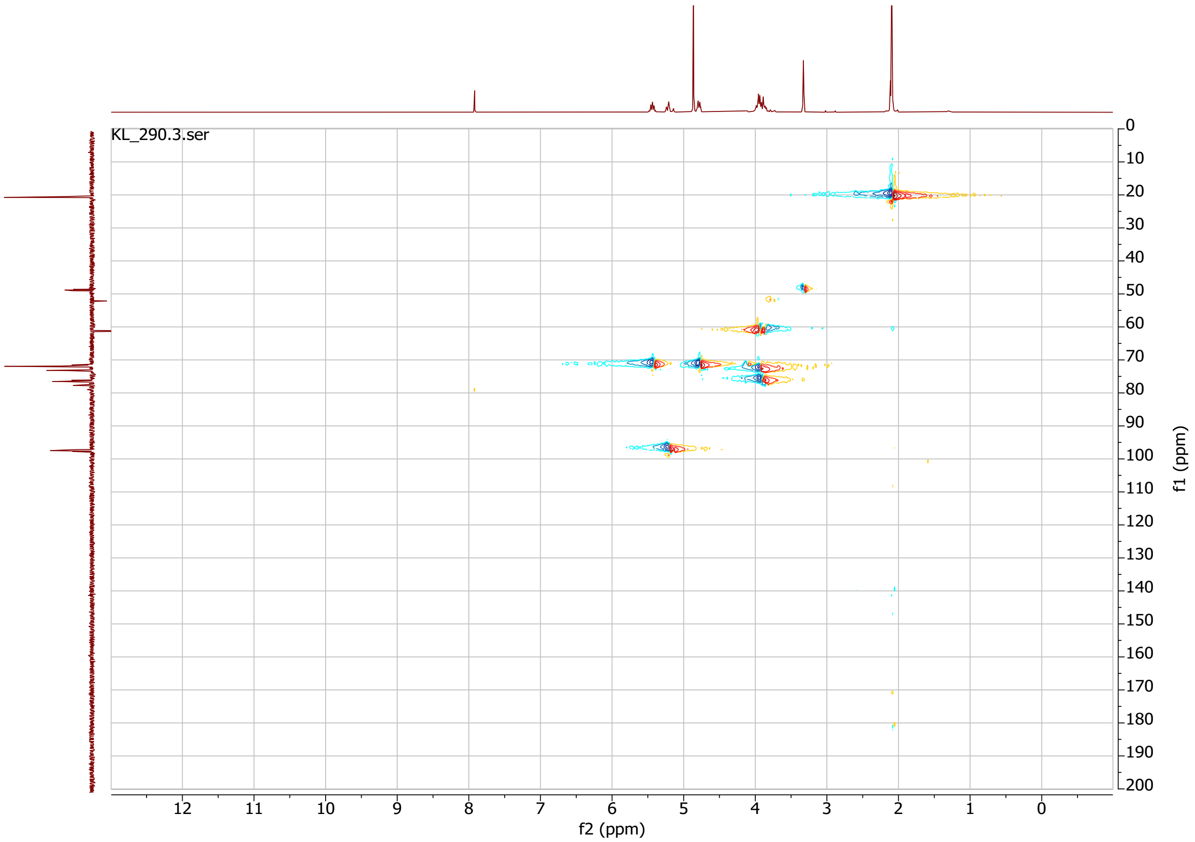

Fig. S42 IR spectrum of **7** in KBr pellet.

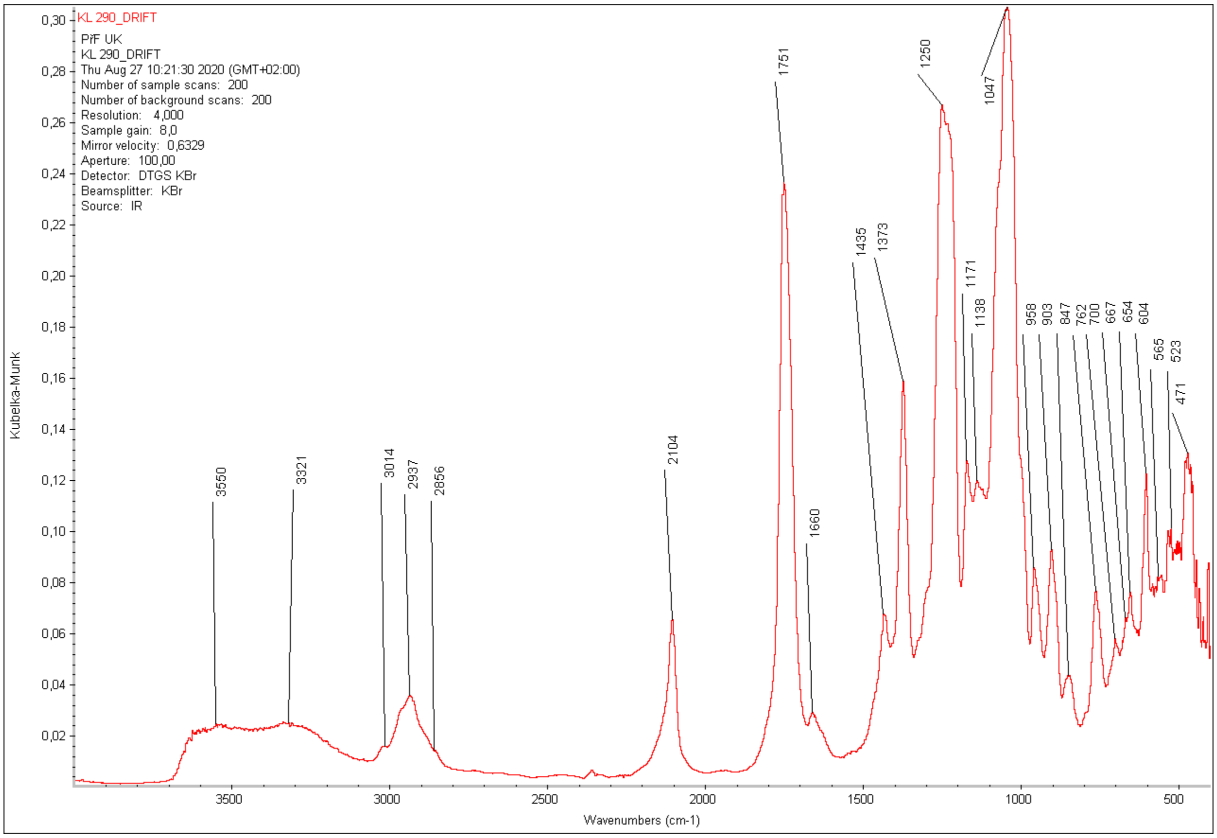

Fig. S43 UV–vis spectrum of **7** in $CHCl_3$.

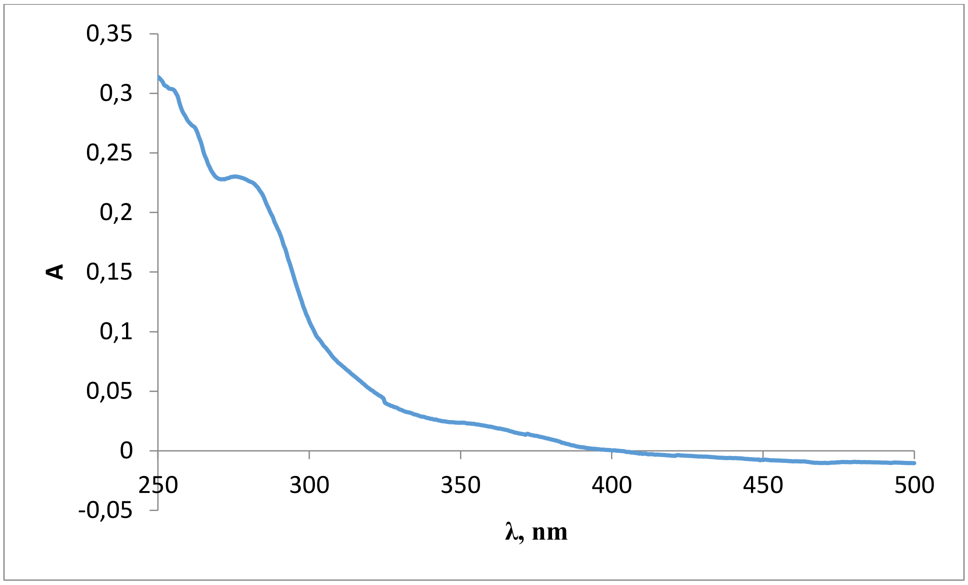

6^A-Azido-6^A-deoxy-hexakis-$6^{B,C,D,E,F,G}$-*O*-methyl-per-2,3-*O*-acetyl-β-cyclodextrin (**8**)

6^A-Azido-6^A-deoxy-per-2,3-*O*-acetyl-β-cyclodextrin (**7**, 2.64 g, 1.51 mmol) was dissolved in purified methyl *p*-toluenesulfonate (26 mL, 177 mmol) in a 100 mL flask. The flask was set in a magnetic stirrer at room temperature under the argon flow. After this, a portion of *N*,*N*-diisopropylethylamine (6.5 mL, 38 mmol) was slowly added to the reaction mixture. Then the process was allowed to proceed at 100 °C. Every two hours, the pH of the reaction mixture was checked by an indicator paper, and small portions of *N*,*N*-diisopropylethylamine (0,5 mL, 2,9 mmol) were added to maintain the basicity. The reaction mixture was finally left overnight when the pH of the mixture had stopped decreasing. After this, the reaction mixture was diluted by chloroform and washed successively with 9/1 ethanol/water solution of NaI, 1 M HCl, saturated solution of $NaHCO_3$, saturated solution of $Na_2S_2O_3$, and brine (all 50 mL). The organic layer was concentrated and applied to a silica gel column (40 g of silica gel). The product (2.25 g; 81% yield) was eluted with chloroform/methanol 75/1 mixture.

^{1}H-NMR (400 MHz, $CDCl_3$) δ=5.28-5.43 (H-C^3, 7H); 5.12-5.23 (H-C^1, 7H); 4.65-4.83 (H-C^2, 7H); 4.16- 3.55 (H-C^4, H-C^5, H-C^6, 28H); 3.42 (Me-O-C^6, 18H); 2.02-2.12 (Me-C(O), 42H). ^{13}C-NMR (100 MHz, $CDCl_3$) δ=96.36 (C^1); 75.55-76.40 (C^4); 69.80-72.01 (C^2, C^3, C^5, $C^{6B,C,D,E,F,G}$); 59.41 (Me-O-C^6); 51.98 (C^{6A}); 20.74 (Me-C(O)). HRMS (ESI): *m/z* calcd for $C_{76}H_{109}N_3O_{48}$+NH_4^+: 1849.65 [M+NH_4^+]; found: 1849.64870; [α]$CHCl_3$= +132.8°.

Fig. S44 ^{1}H-NMR spectrum of **8** in $CDCl_3$ at 25 °C.

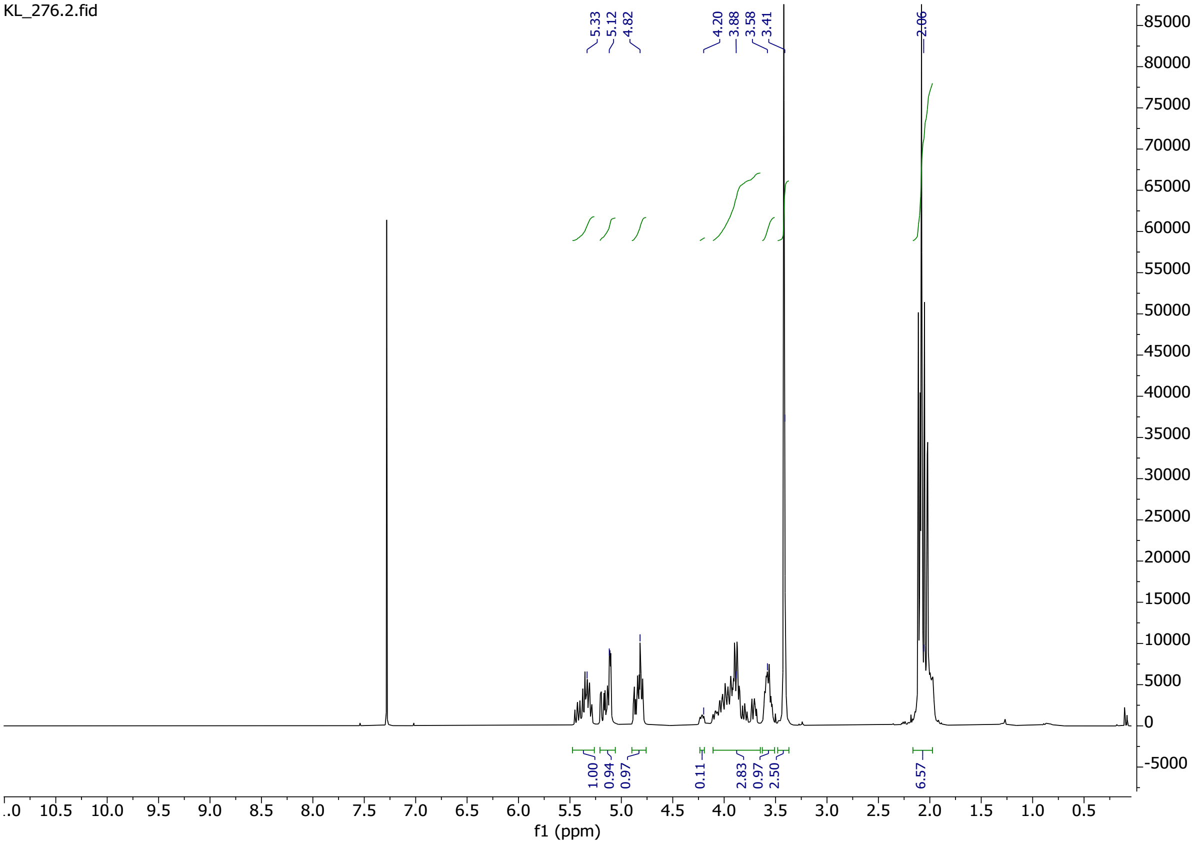

Fig. S45 DEPT-^{13}C-NMR spectrum of **8** in $CDCl_3$ at 25 °C.

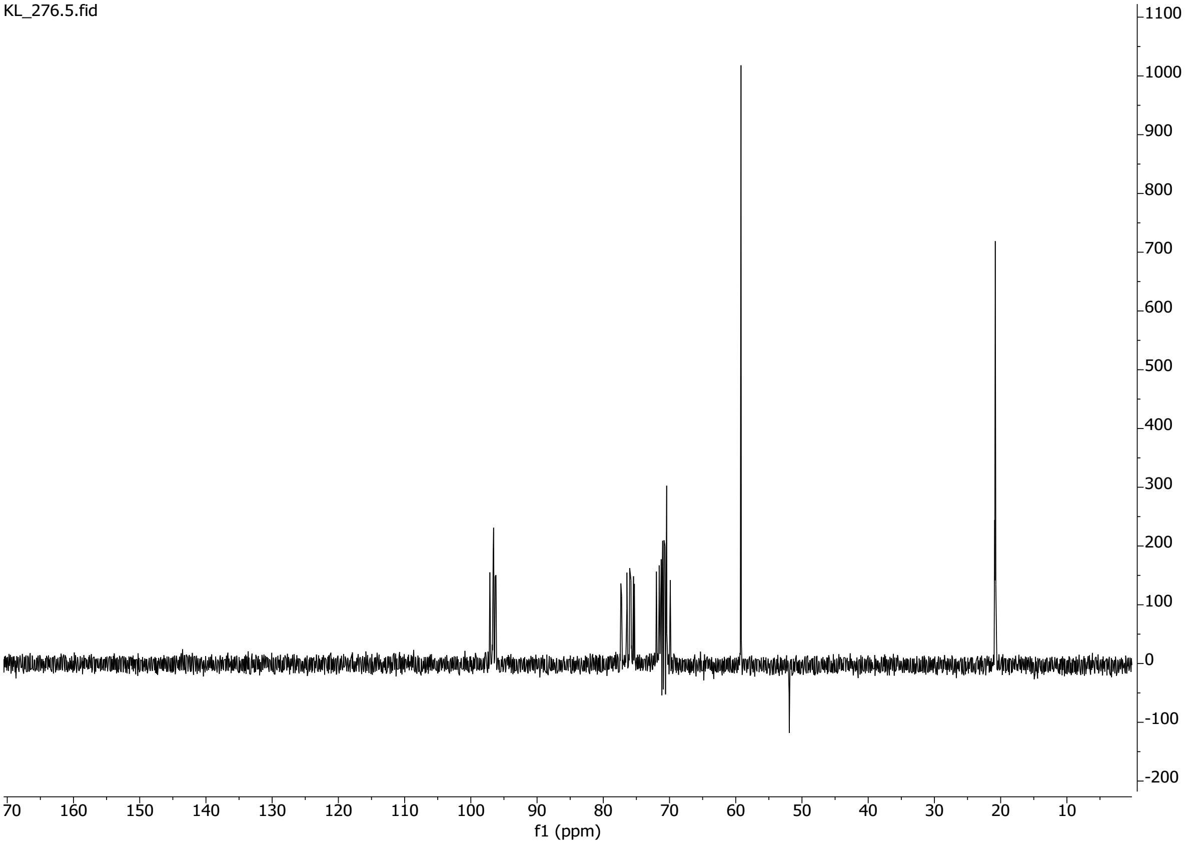

Fig. S46 COSY spectrum of **8** in $CDCl_3$ at 25 °C.

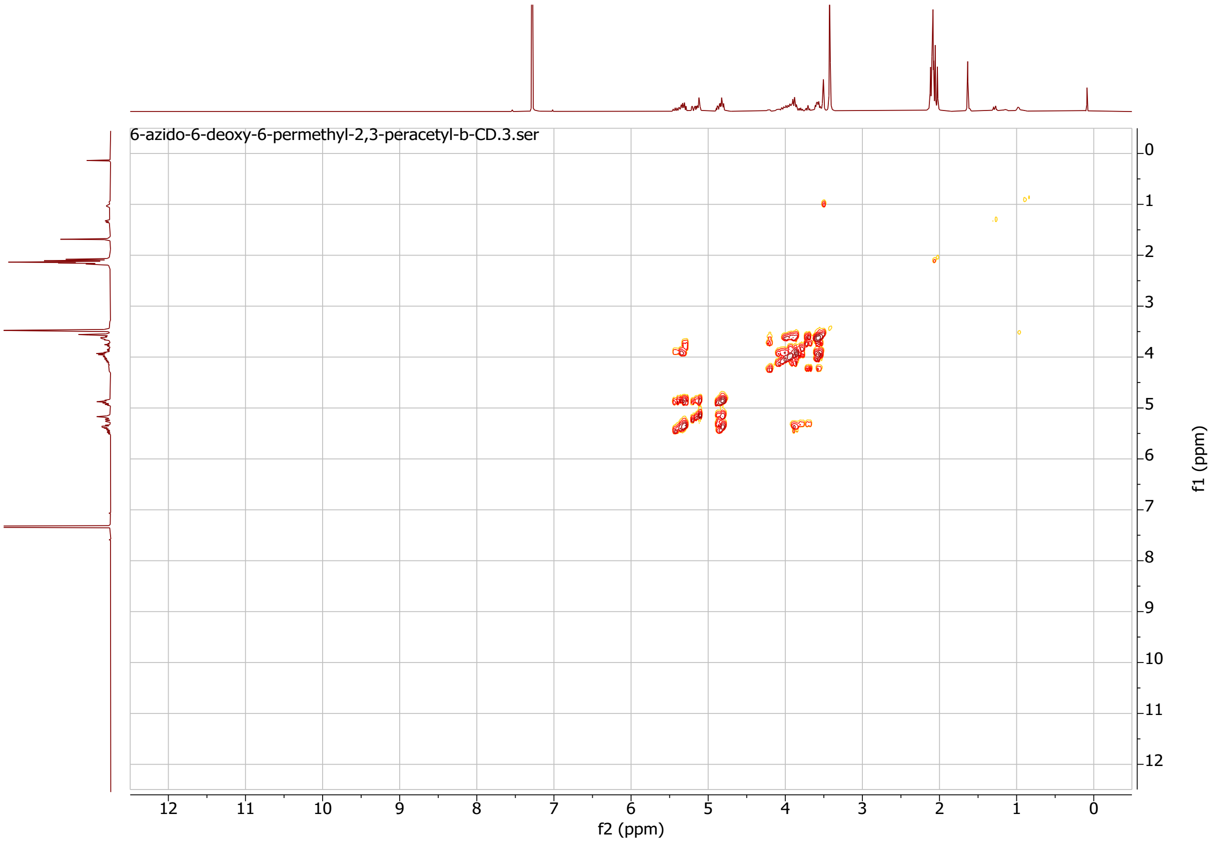

Fig. S47 HSQC spectrum of **8** in $CDCl_3$ at 25 °C.

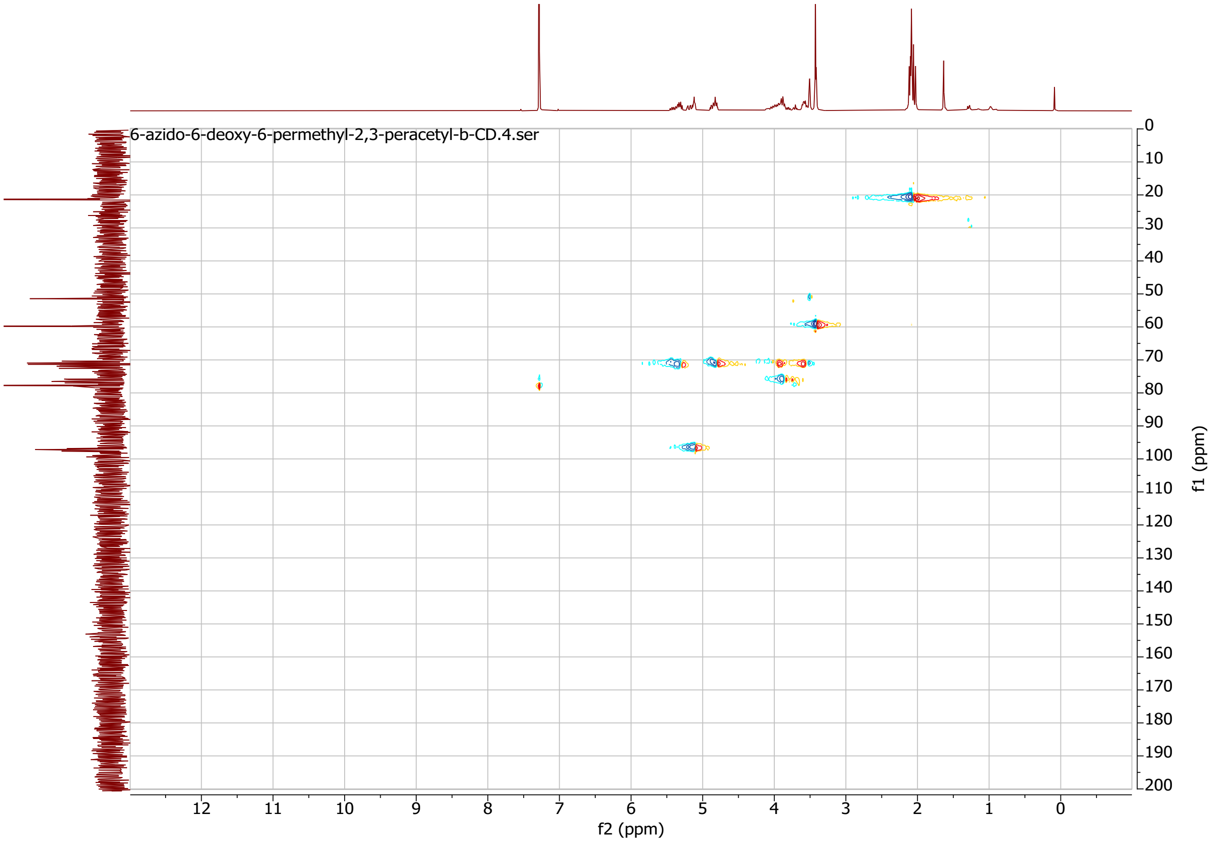

Fig. S48 IR spectrum of **8** in KBr pellet.

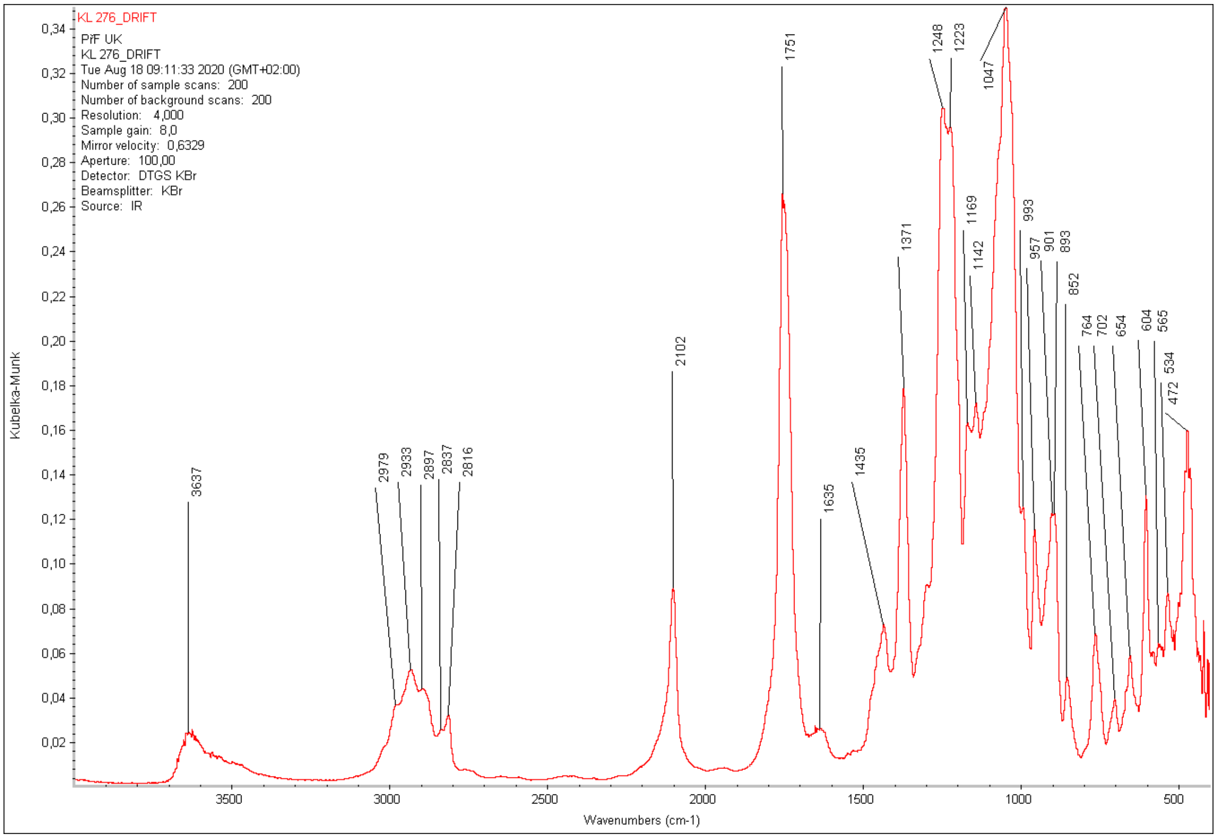

Fig. S49 UV–vis spectrum of **8** in $CHCl_3$.

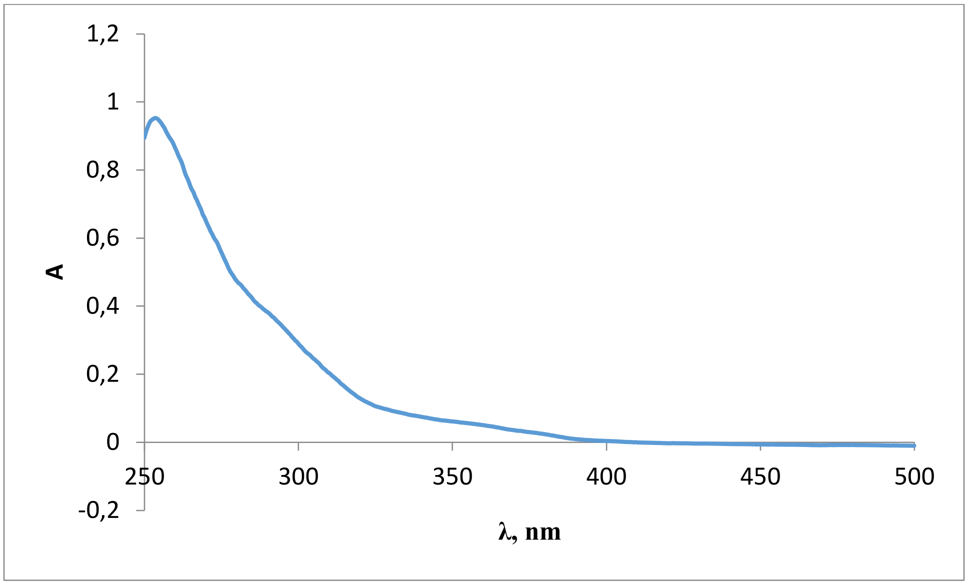

Bis-1,3-[1-(6^{A}-deoxy-hexakis-6^{B,C,D,E,F,G}-*O*-methyl-per-2,3-*O*-acetyl-β-cyclodextrin-6^{A}-*C*-yl)-1,2,3-triazol-4-yl]methoxymethylene (**9**)

6^{A}-Azido-6^{A}-deoxy-hexakis-6^{B,C,D,E,F,G}-*O*-methyl-per-2,3-*O*-acetyl-β-cyclodextrin (**8**, 1 g; 0.546 mmol) was dissolved in DMF (25 mL), and the solution was bubbled by argon for 30 minutes. Then, portions of 0.5 M propargyl ether solution in DMF (0.49 mL; 0.2457 mmol), copper sulfate hydrate (0.136 g; 0.546 mmol), and sodium ascorbate (0.105 g; 0.546 mmol) were added to the reaction mixture, and the reaction mixture was allowed to stir at room temperature. After 72 hours, the temperature increased to 50 °C, and the reaction mixture was stirred for another 48h. After that, the solvent was removed in vacuo at 90 °C, and a column chromatography (30 g silica gel) with chloroform/methanol 50/1 to 25/1 mixture gave 0.6 g of the desirable product (60% yield).

^{1}H-NMR (400 MHz, $CDCl_3$) δ=7.68 (s, H-triazole, 2H); 5.23-5.44 (H-C^{3}, 14H); 5.05-5.2 (H-C^{1}, 14H); 4.63-4.96 (H-C^{2}, -CH_2-triazole, 18H); 3.27-4.37 (H-C^{4}, H-C^{5}, H-C^{6}, Me-O-C^{6}); 2.02-2.12 (Me-C(O), 42H). ^{13}C-NMR (100 MHz, $CDCl_3$) δ=125.63 (C-triazole); 95.65-96-94 (C^{1}); 75.13-76.37 (C^{4}); 69.63-72.10 (C^{3}, C^{5}, C^{2}, C^{6}); 63.77 (–CH_2-triazole); 20.51-20.90 (Me-C(O)). HRMS (ESI): *m/z* calcd for $C_{158}H_{224}N_6O_{97}$+H^++Na^+: 1889.15 [M+H^++Na^+]; found 1889.15856.

Fig. S50 ^{1}H-spectrum of **9** in $CDCl_3$ at 25 °C.

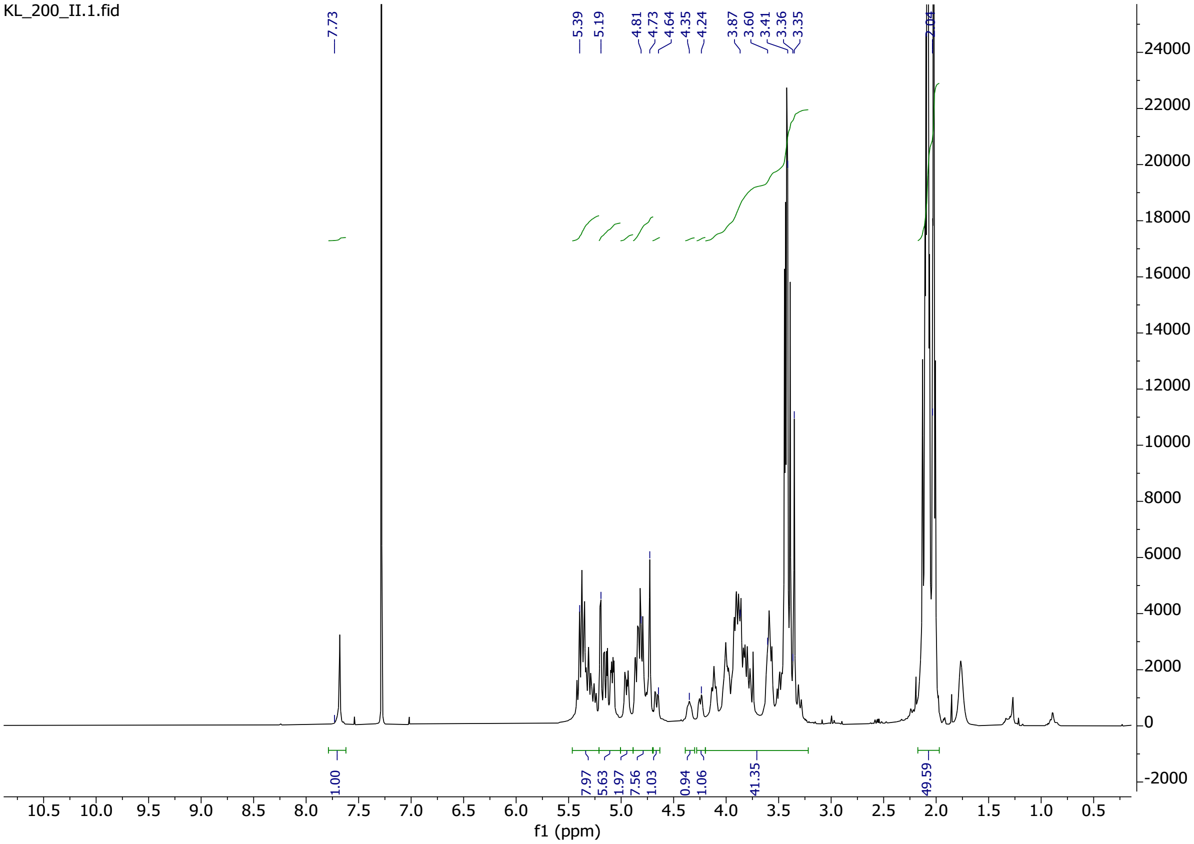

Fig. S51 DEPT-^{13}C-NMR spectrum of **9** in $CDCl_3$ at 25 °C.

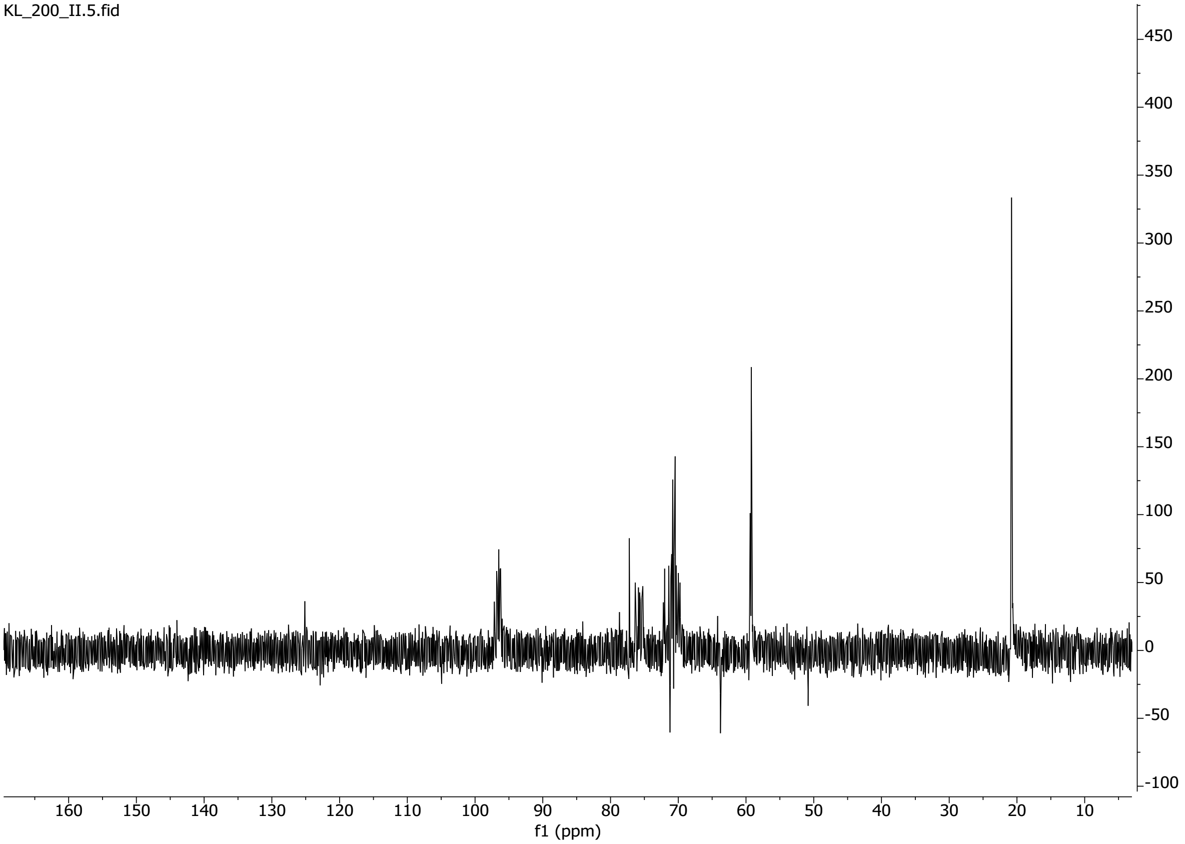

Fig. S52 COSY spectrum of **9** in $CDCl_3$ at 25 °C.

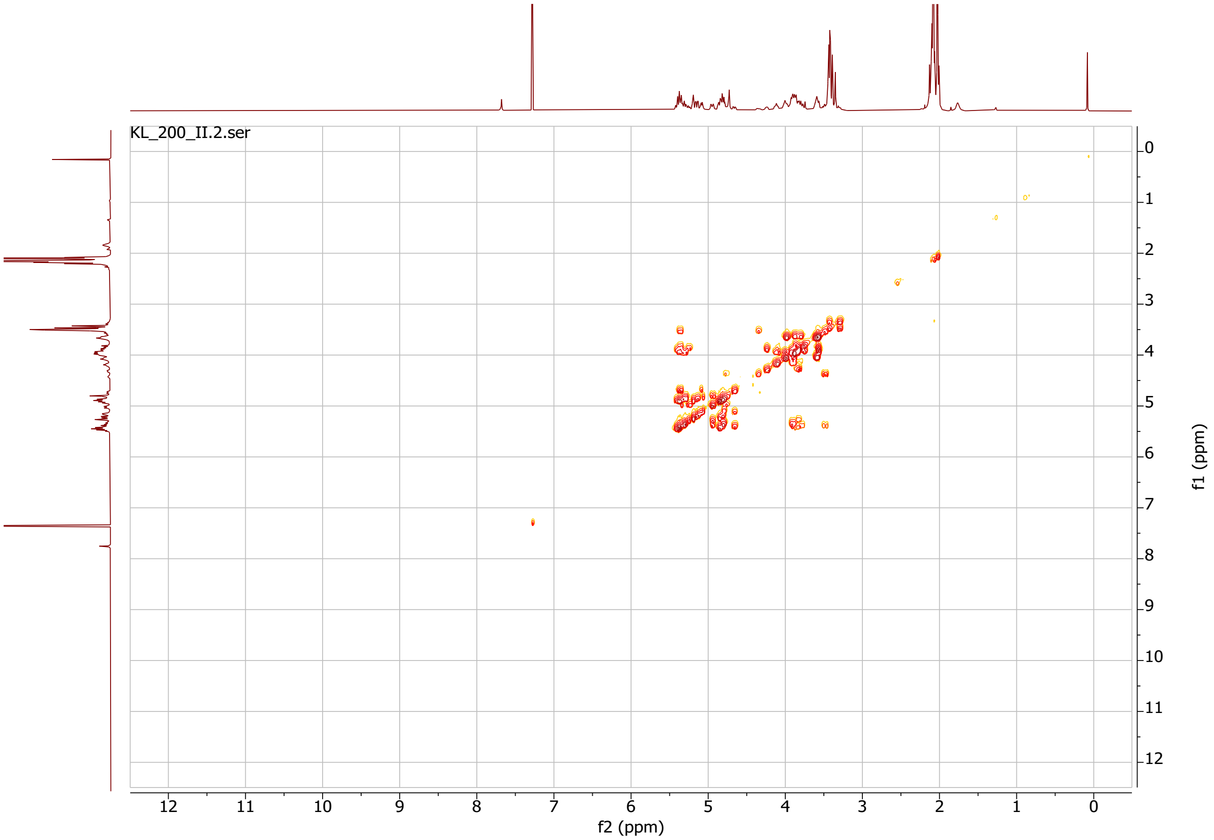

Fig. S53 HSQC spectrum of **9** in $CDCl_3$ at 25 °C.

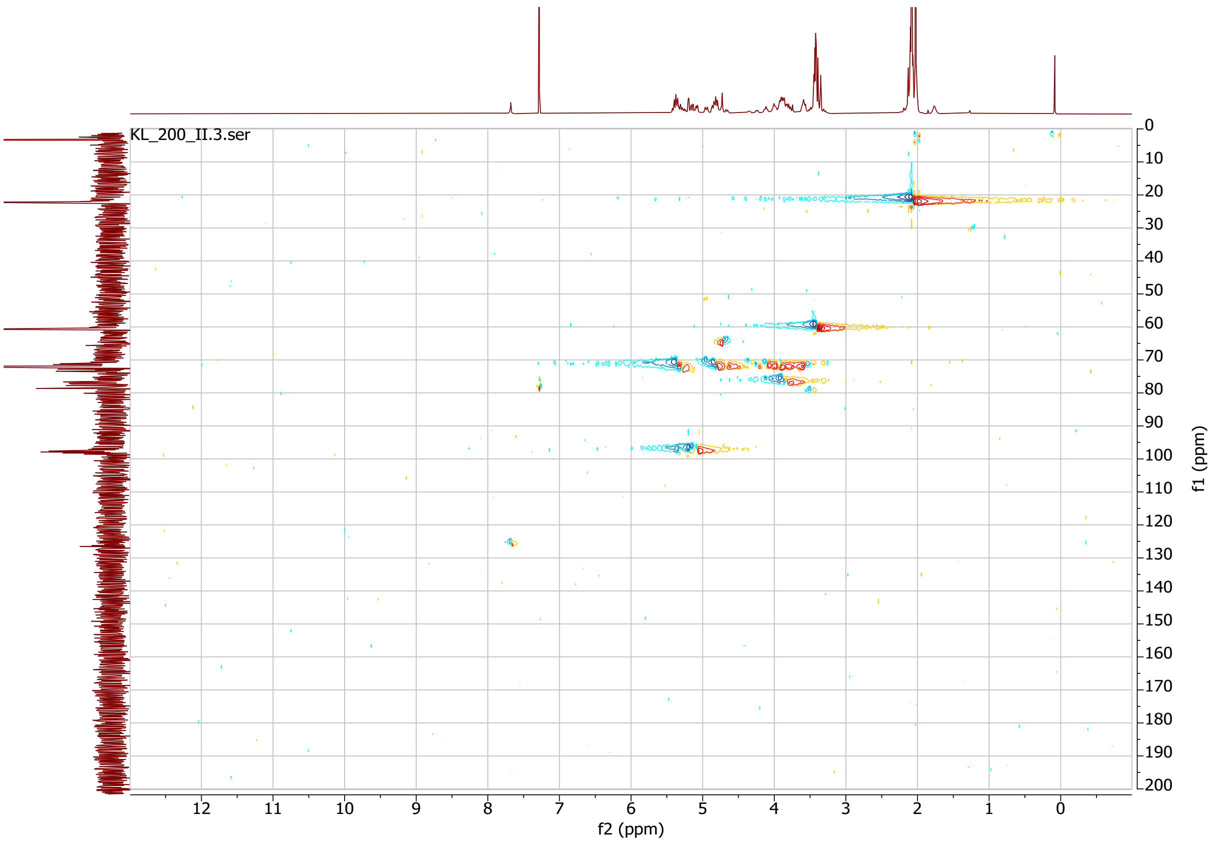

Fig. S54 NOESY spectrum of **9** in $CDCl_3$ at 25 °C.

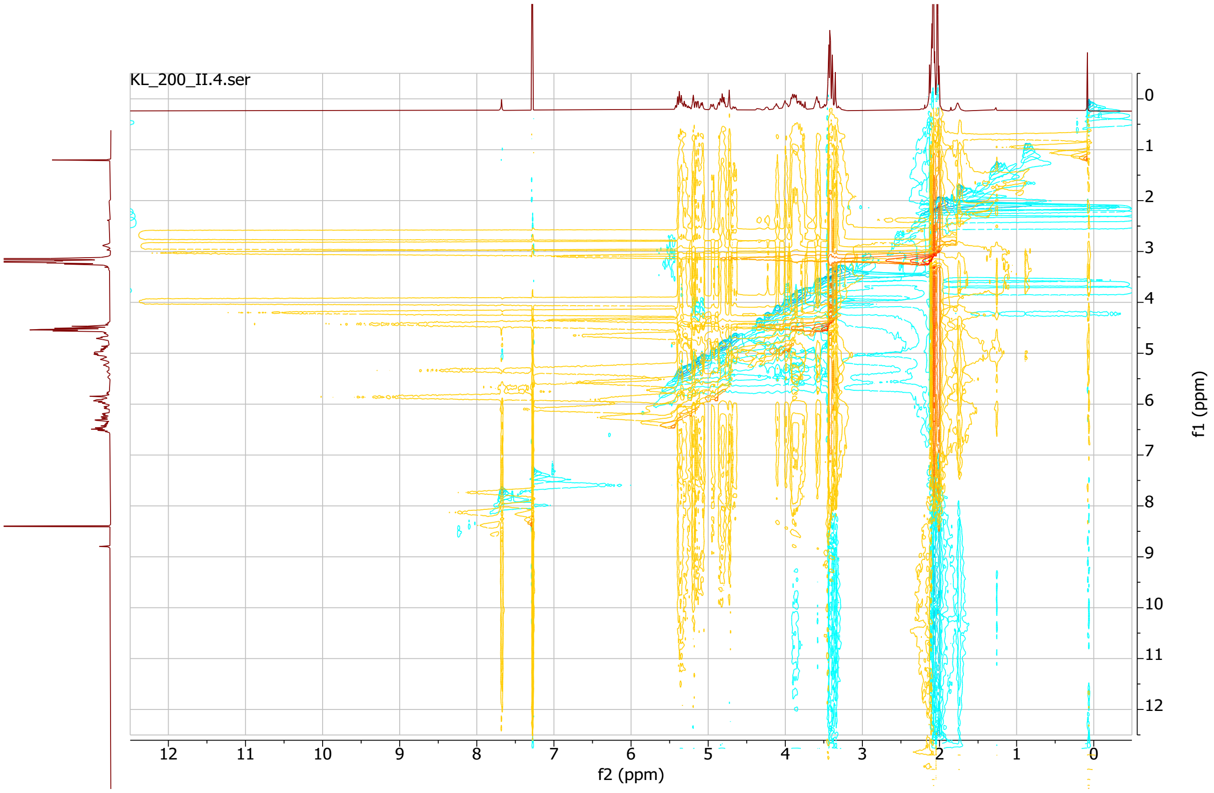

Bis-1,3-[1-(6^{A}-deoxy-hexakis-6^{B,C,D,E,F,G}-*O*-methyl-β-cyclodextrin-6^{A}-yl)-1,2,3-triazol-4-yl]methoxymethylene (**10**)

Bis-1,3-[1-(6^{A}-deoxy-hexakis-6^{B,C,D,E,F,G}-*O*-methyl-per-2,3-*O*-acetyl-β-cyclodextrin-6^{A}-*C*-yl)-1,2,3-triazol-4-yl]methoxymethylene (**9**, 0.6 g; 0.165 mmol) was treated by 1 mL of 1 M solution of sodium methoxide in 20 mL of methanol for 1 h at room temperature. The solution was neutralized by 10 g of Amberlite 120 resin and dried to get the final product (0.38 g; 97% yield).

^{1}H-NMR (400 MHz, D_2O) δ=8,04 (s, H-triazole, 2H); 4,88-5,1 (H-C^{1}, H-C^{6A}, 18H); 4.69 (–CH_2-triazole overlapped with H_2O); 4.48-4.56 (H-C^{6A}, 2H, diastereotopic); 4.17-4.23 (H-C^{5A}, 2H); 3,41-4.06 (H-C^{2}, H-C^{3}, H-C^{4}, H-C^{5}, H-C^{6}); 3,05-3,35 (Me-O-C^{6}, 18H); 2.64-2.98 (H-C^{6} 4H). ^{13}C-NMR (100 MHz, D_2O) δ=126.48 (C-triazole); 101.34-102.13 (C^{1}); 80.44-83.27 (C^{2}); 69.08-73.04 (C^{3}, C^{4}, C^{5}, C^{6}); 62.78 (–CH_2-triazole, C^{6}); 58.43 (Me-C^{6}); 51.21 (C^{6A}). HRMS (ESI): *m/z* calcd for $C_{102}H_{168}N_6O_{69}+2H^+$: 1291.99 [$M+2H^+$] found: 1291.99549; [α]DMF=+106.3°.

Fig. S55 ^{1}H-NMR spectrum of **10** in D_2O at 25 °C.

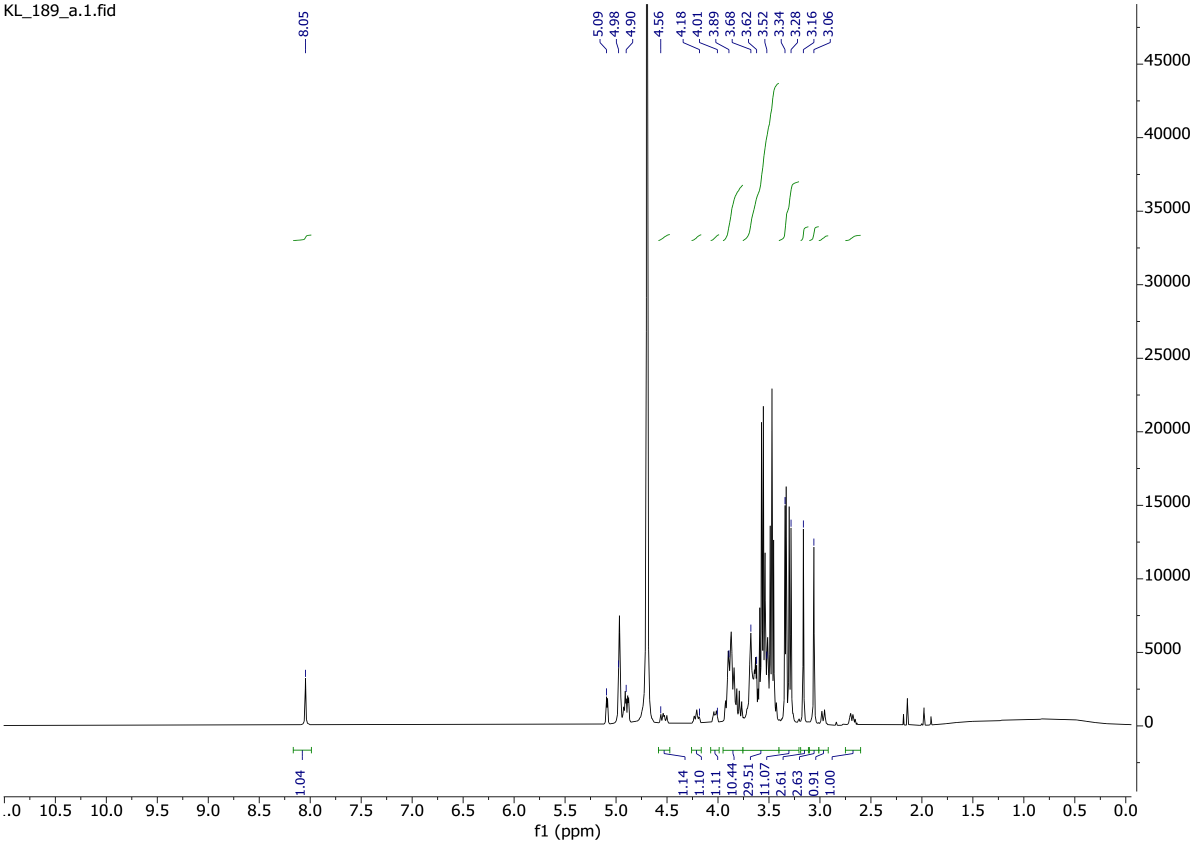

Fig. S56 DEPT-^{13}C-NMR spectrum of **10** in D_2O at 25 °C.

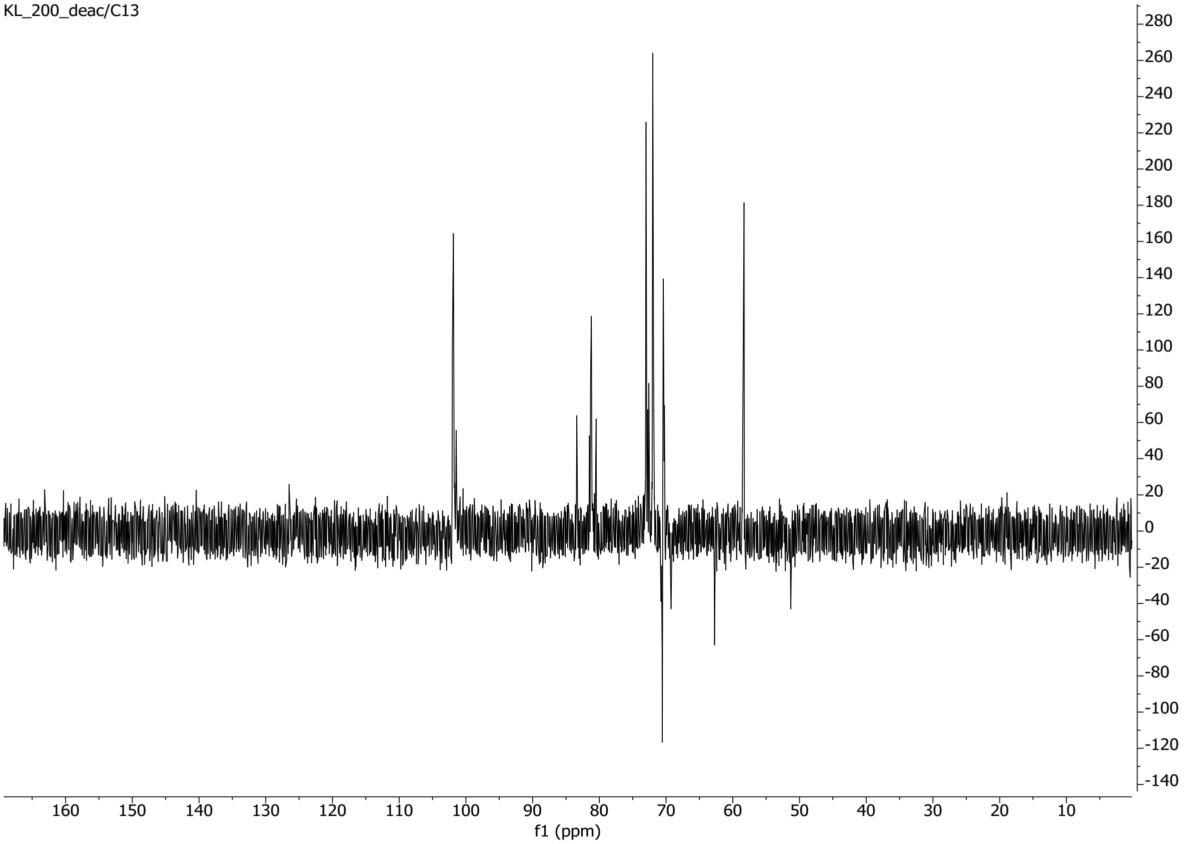

Fig. S57 COSY spectrum of **10** in D_2O at 25 °C.

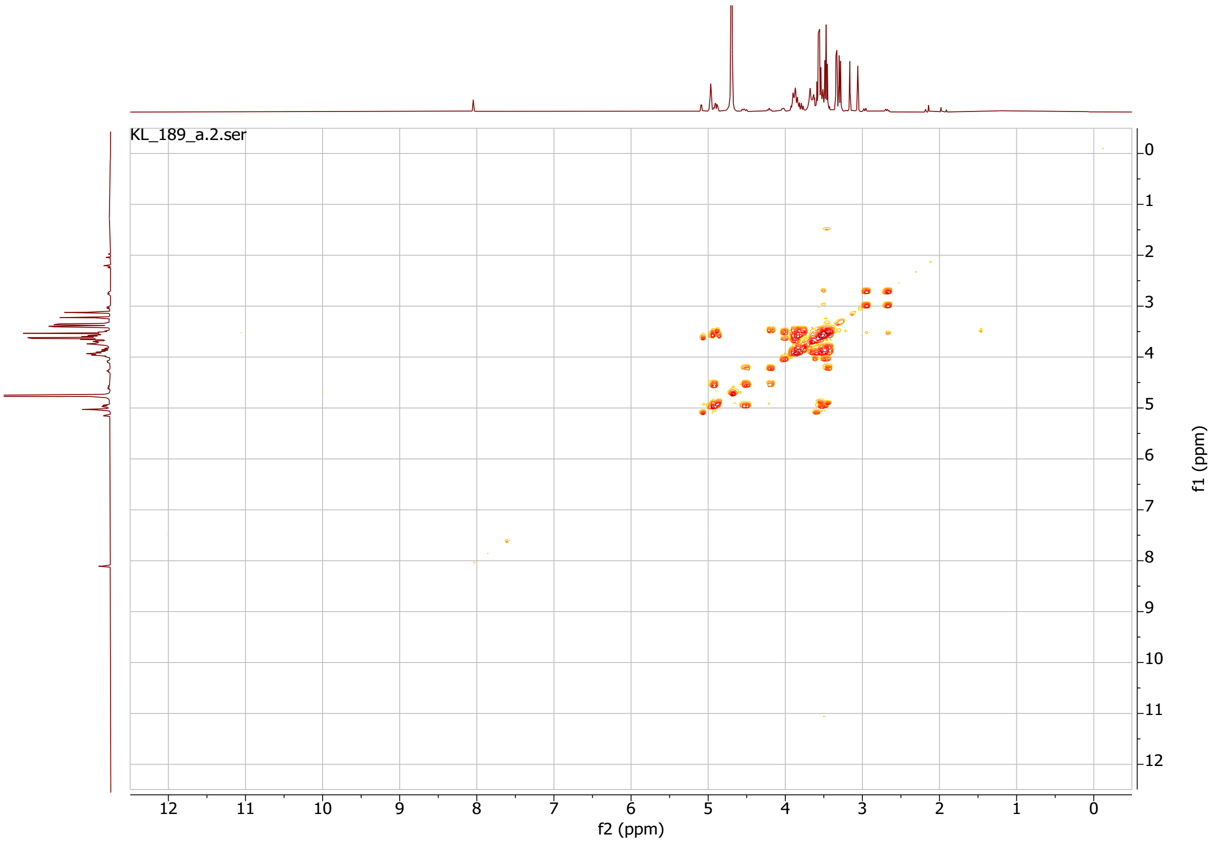

Fig. S58 HSQC spectrum of **10** in D_2O at 25 °C.

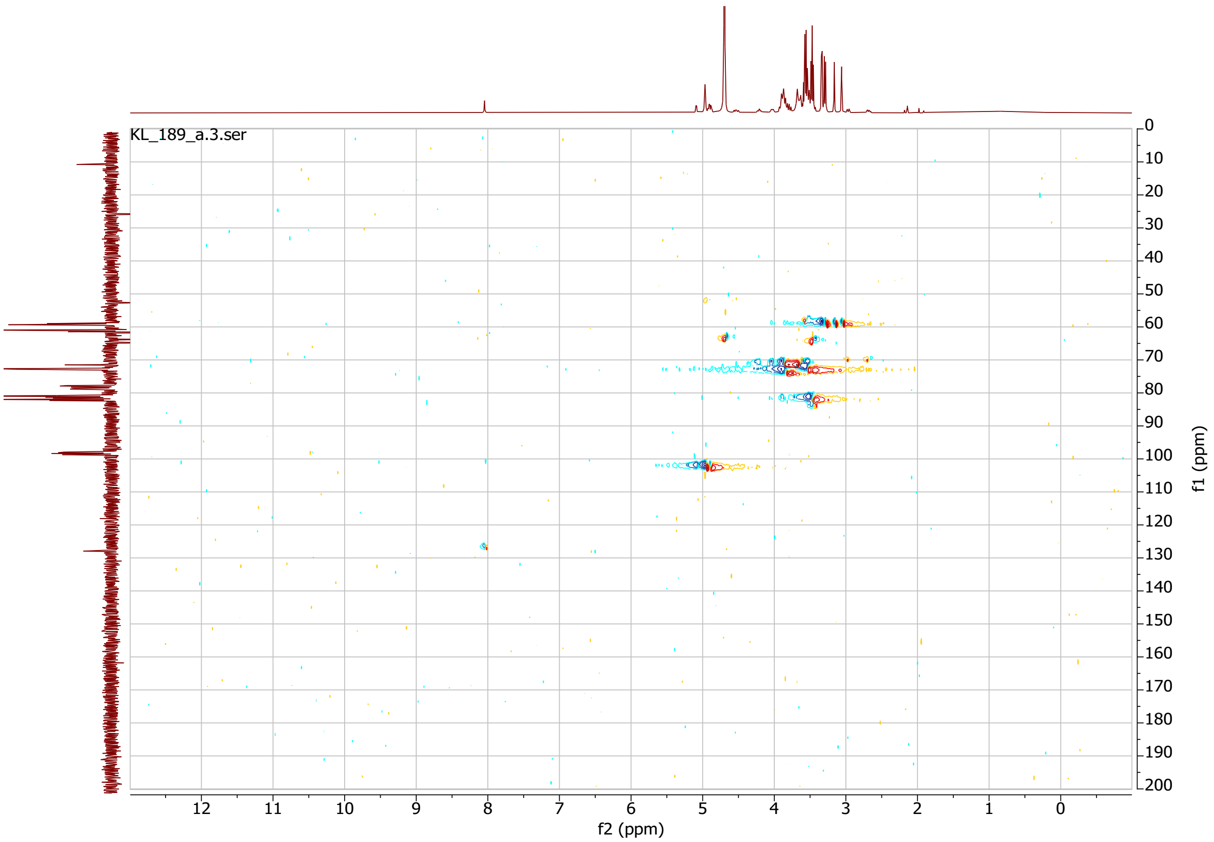

Fig. S59 NOESY spectrum of **10** in D_2O at 25 °C.

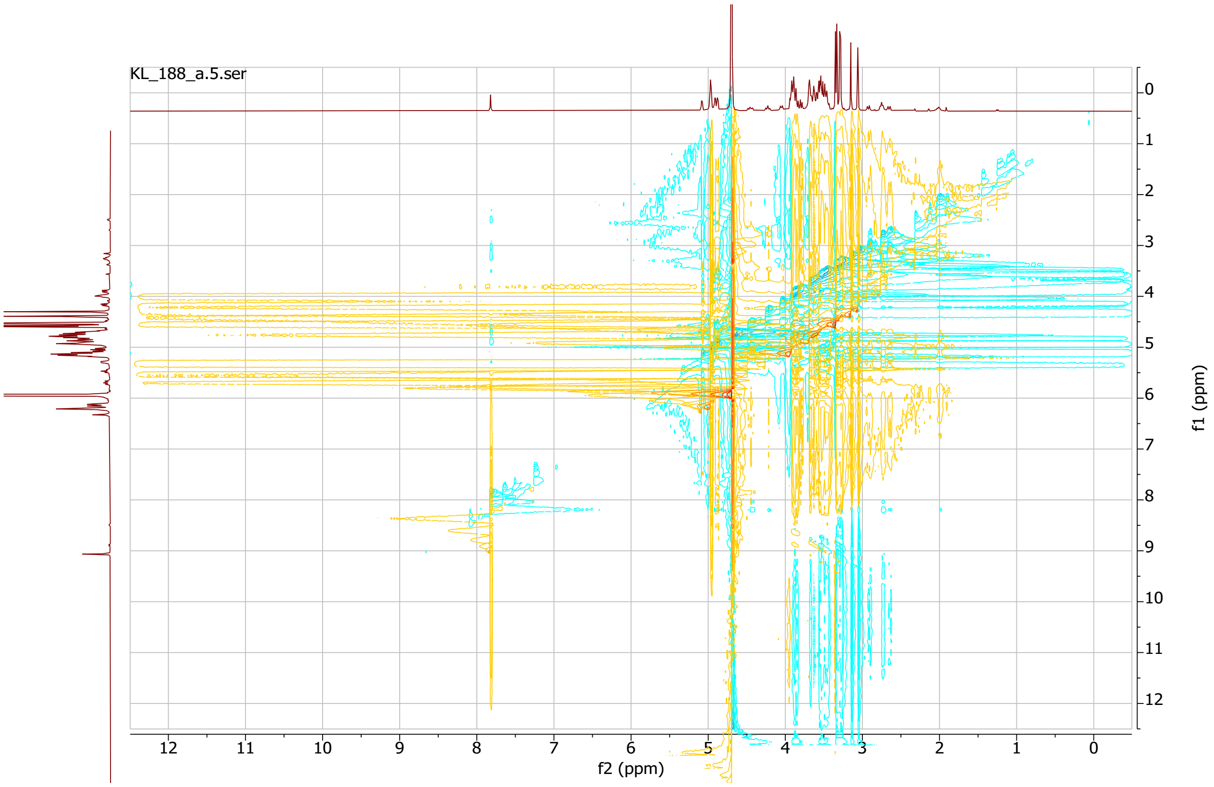

Fig. S60 IR spectrum of **10** in KBr pellet.

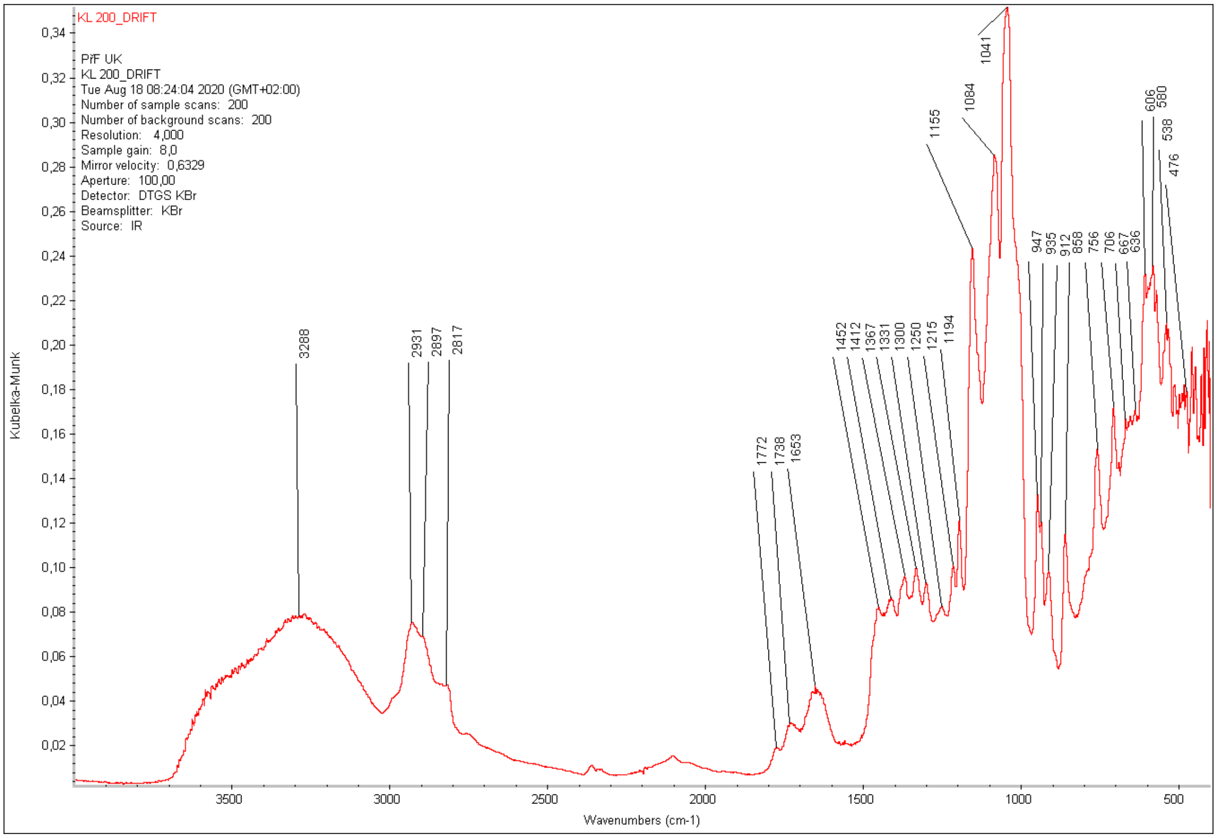

Fig. S61 UV–vis spectrum of **10** in H_2O.

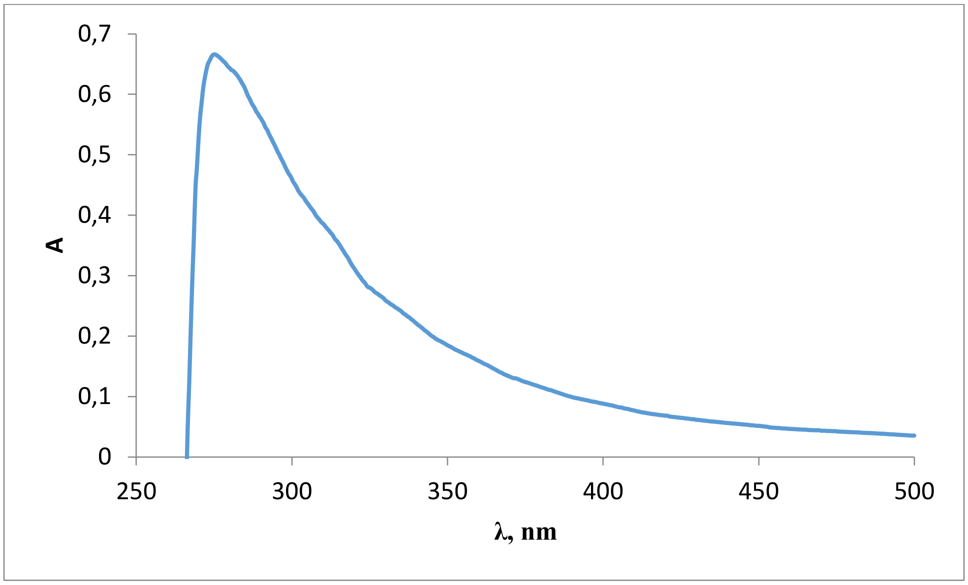

6^A-Azido-6^A-deoxy-hexakis-$6^{B,C,D,E,F,G}$-*O*-methyl -β-cyclodextrin (**11**)

6^A-Azido-6^A-deoxy-hexakis-$6^{B,C,D,E,F,G}$-*O*-methyl-per-2,3-*O*-acetyl-β-cyclodextrin (**8**, 0.8 g; 0.643 mmol) was treated by 1 mL of 1 M solution of sodium methoxide in 20 mL of methanol for 1 h at room temperature. The solution was neutralized by 10 g of Amberlite 120 resin and dried to get the final product in the quantitative yield.

^{1}H-NMR (400 MHz, [d_6] DMSO) δ= 6.08-6.47 (HO-C^2, HO-C^3, 14H); 4.71-4.84 (H-C^1, 7H); 3.15-3.79 (H-C^2, H-C^3, H-C^4, H-C^5, H-C^6, Me-O-C^6 (overlapped with H_2O). ^{13}C-NMR (100 MHz, [d_6] DMSO) δ=103.28 (C^1); 82.65-83.94 (C^2); 70.60-73.44 (C^3, C^4, C^5, C^6); 69.51, 61.00 (C^6); 58.54 (Me-C^6, C^{6A}). HRMS (ESI): *m/z* calcd for $C_{48}H_{81}N_3O_{34}+NH_4^+$: 1261.50 [$M+NH_4^+$]; found: 1261.49846. [α]DMF=+140.8°.

Fig. S62 ^{1}H-NMR spectrum of **11** in [d_6]-DMSO at 25 °C.

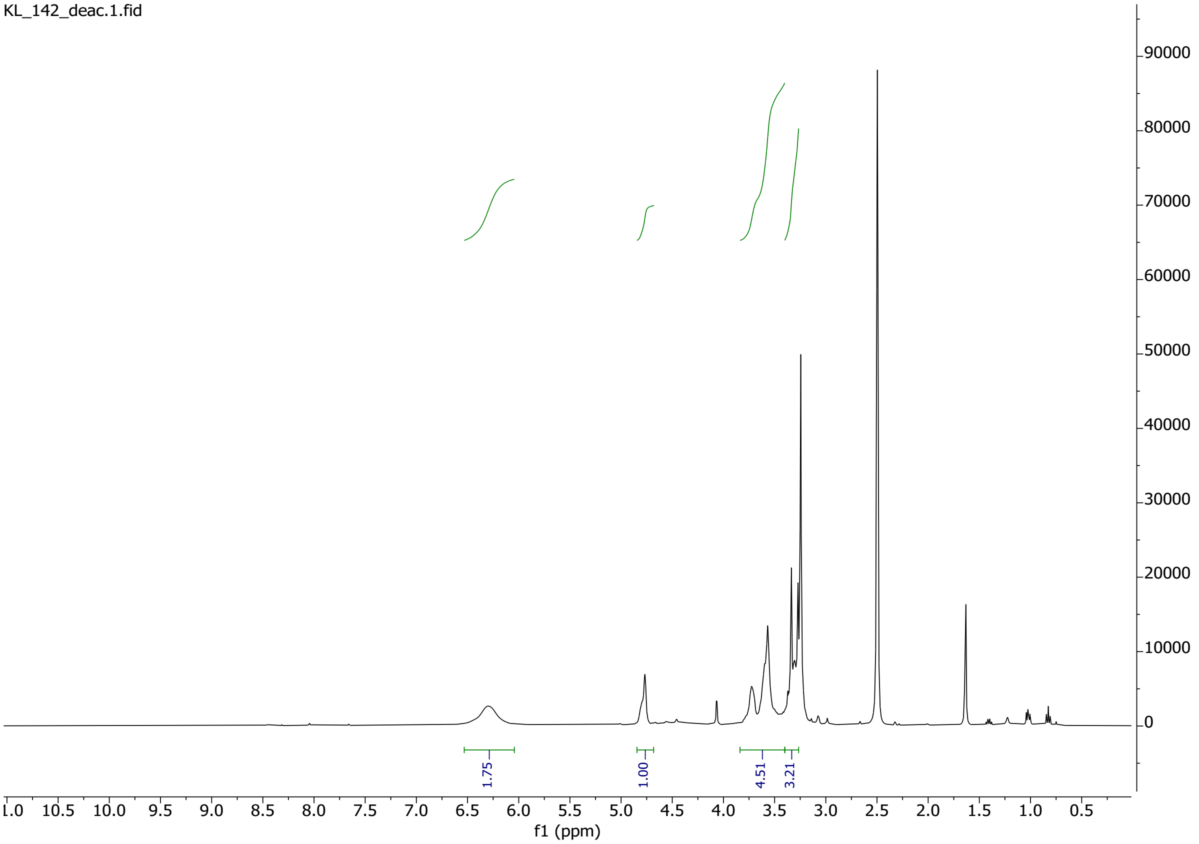

Fig. S63 DEPT-^{13}C-NMR spectrum of **11** in [d_6]-DMSO at 25 °C.

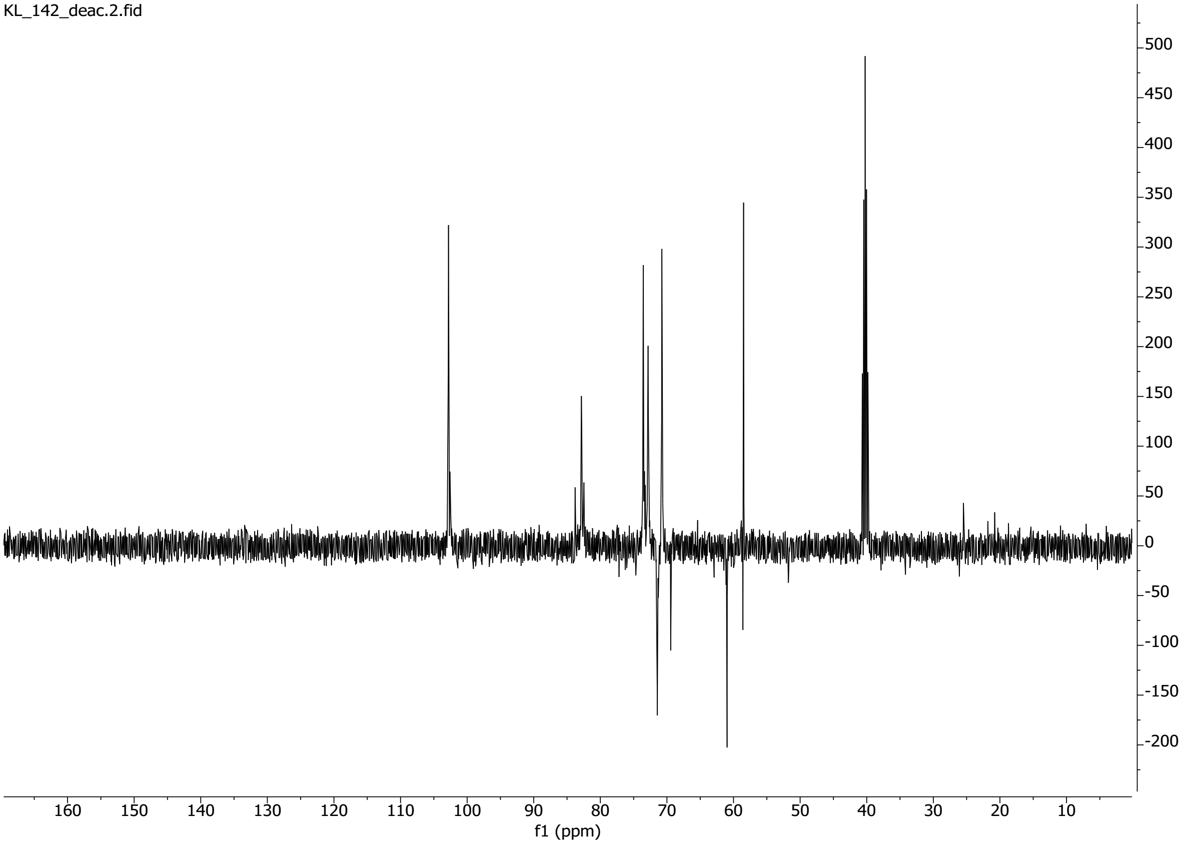

Fig. S64 COSY spectrum of **11** in [d_6]-DMSO at 25 °C.

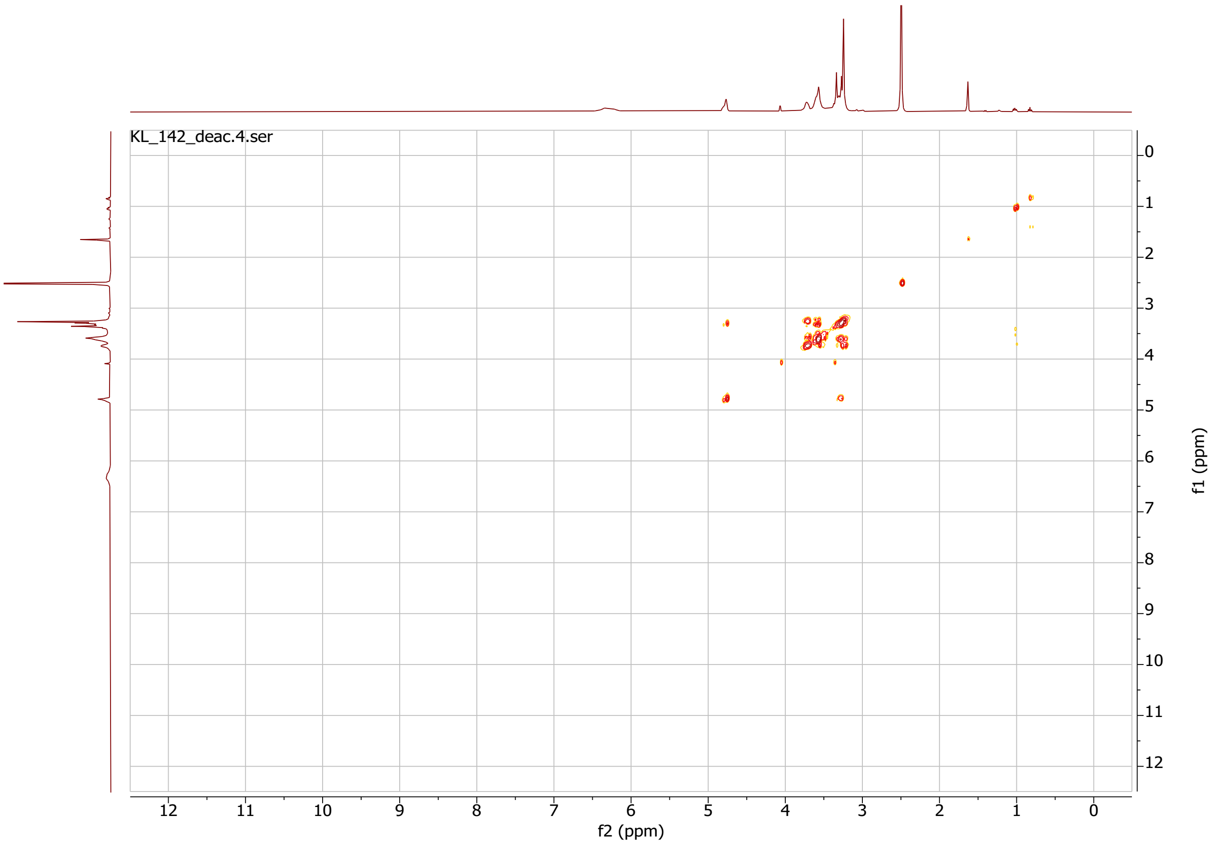

Fig. S65 HSQC spectrum of **11** in [d_6]-DMSO at 25 °C.

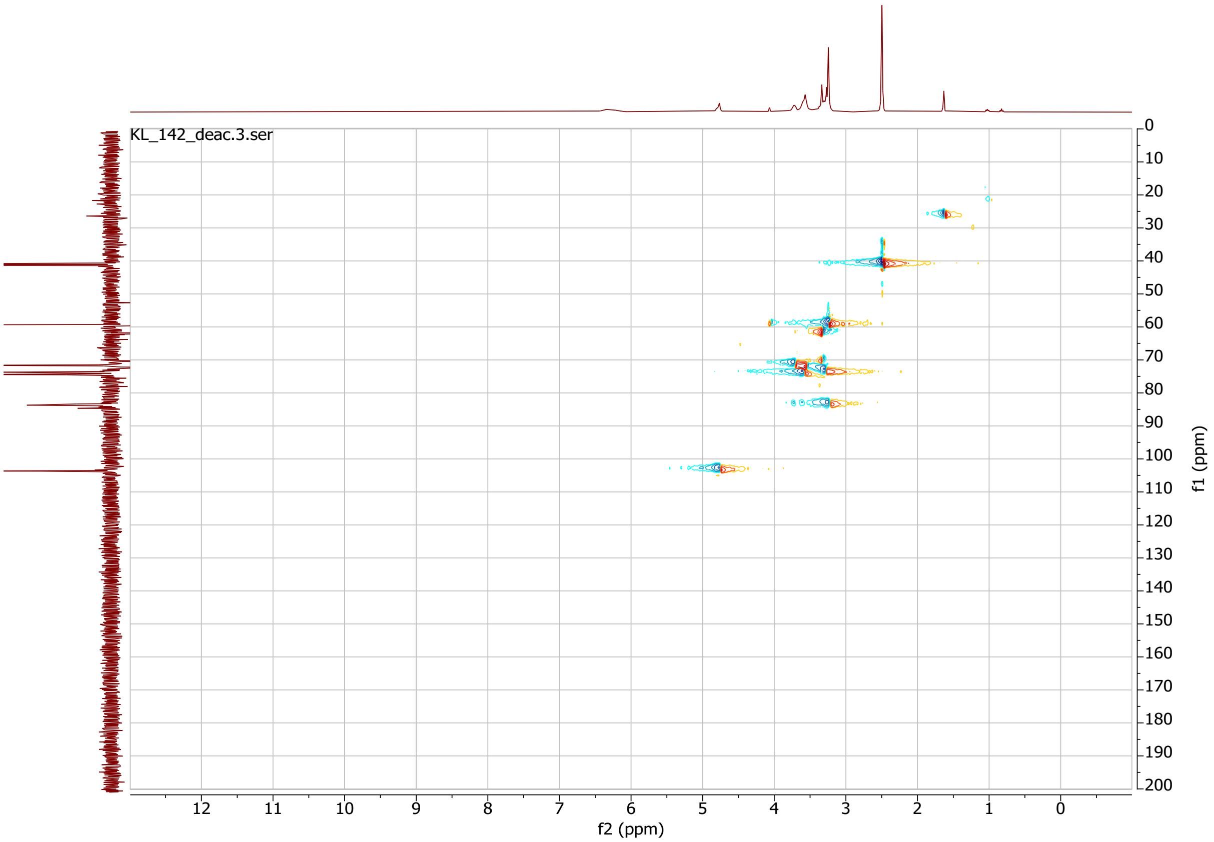

Fig. S66 IR spectrum of **11** in KBr pellet.

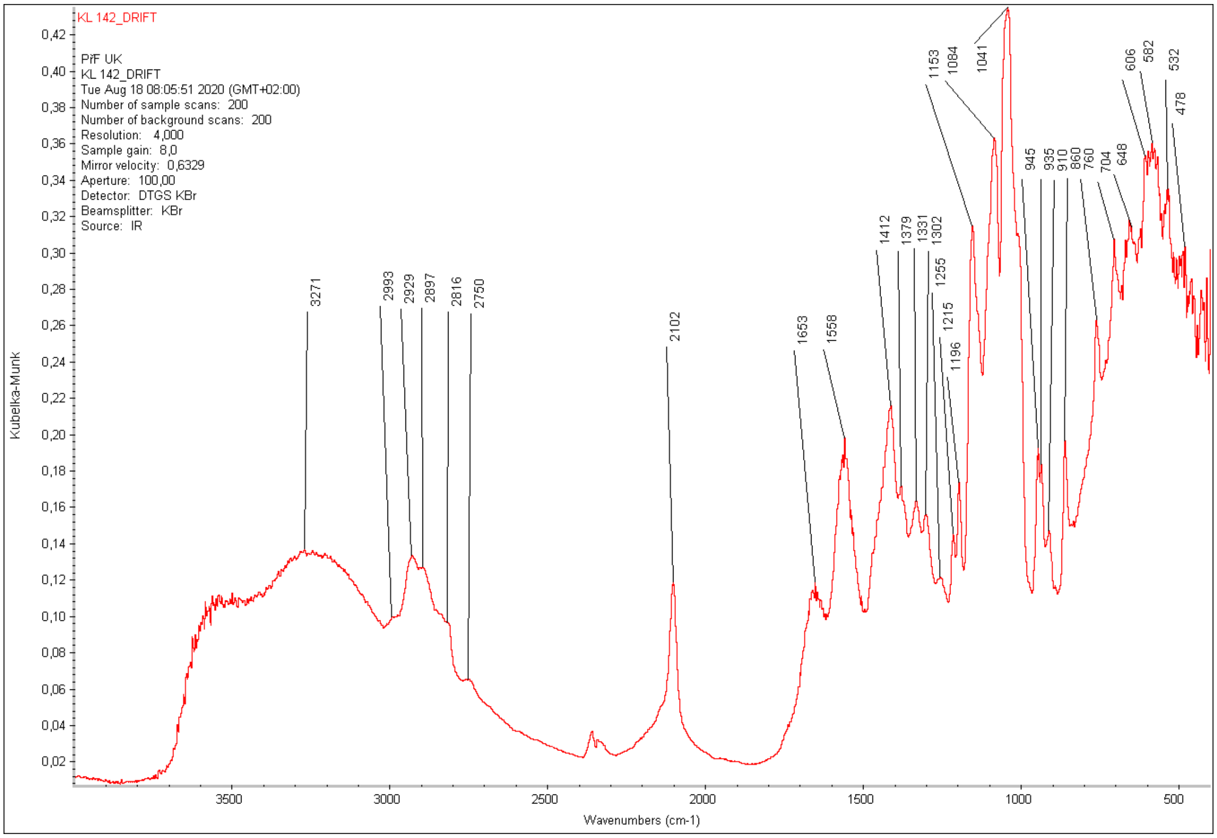

Fig. S67 UV–vis spectrum of **11** in DMSO.

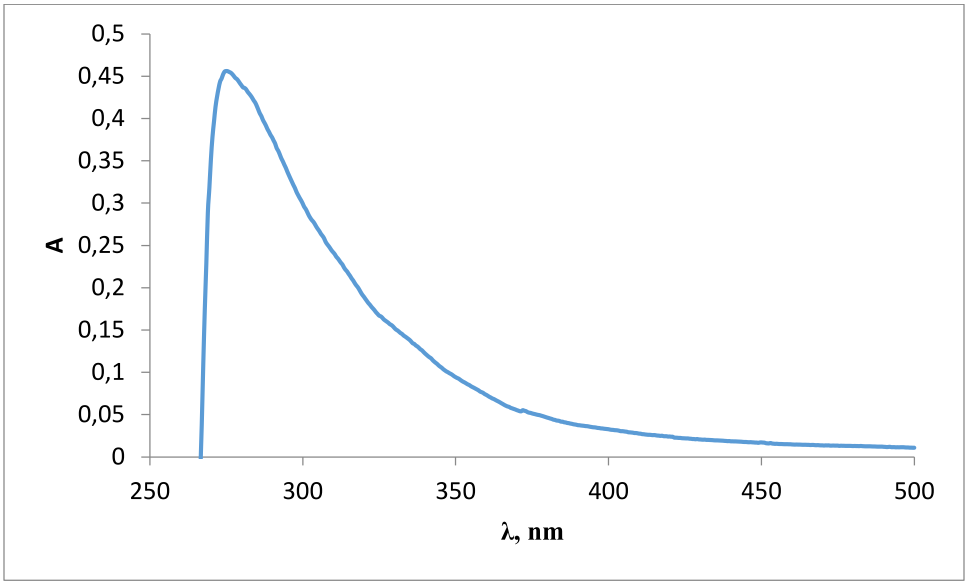

N,N'-Bis(6^{A}-deoxy-$6^{B,C,D,E,F,G}$-*O*-hexakis-methyl-β-cyclodextrin-6^{A}-C-yl)urea (12)

6^{A}-Azido-6^{A}-deoxy-hexakis-$6^{B,C,D,E,F,G}$-*O*-methyl-β-cyclodextrin (**11**, 0.1 g; 0.0804 mmol) together with triphenylphosphine (0.05 g; 0.1907 mmol) and lithium iodide (0.023 g; 0.1717 mmol) were dissolved in dry DMF (19 mL). The reaction mixture was bubbled by dry CO_2 at 40 °C overnight. The solvent was distilled off, and column chromatography (10 g silica gel) with acetonitrile/water/ conc. aq. NH_3 12/3/1 afforded 0.04 g of the desirable product (41% yield).

^{1}H-NMR (400 MHz, D_2O) δ=4.93-4.99 (H-C^{1}, 14H); 3.45-3.91 (H-C^{2}, H-C^{3}, H-C^{4}, H-C^{5}, H-C^{6}); 3.33 (Me-O-C^{6}, 36H). ^{13}C-NMR (100 MHz, D_2O) δ=101.92 (C^{1}); 81.47 (C^{2}); 72.96 (C^{4}); 72.15 (C^{5}); 71.13 (C^{6}); 70.38 (C^{5}); 58.63 (Me-C^{6}). HRMS (ESI): *m/z* calcd for $C_{97}H_{164}N_2O_{69}$+2H^{+}: 1231.98 [M+2H^{+}]; found: 1231.96992; [α]DMF=+115.4°.

Fig. S68 ^{1}H-NMR spectrum of **12** in D_2O at 25 °C.

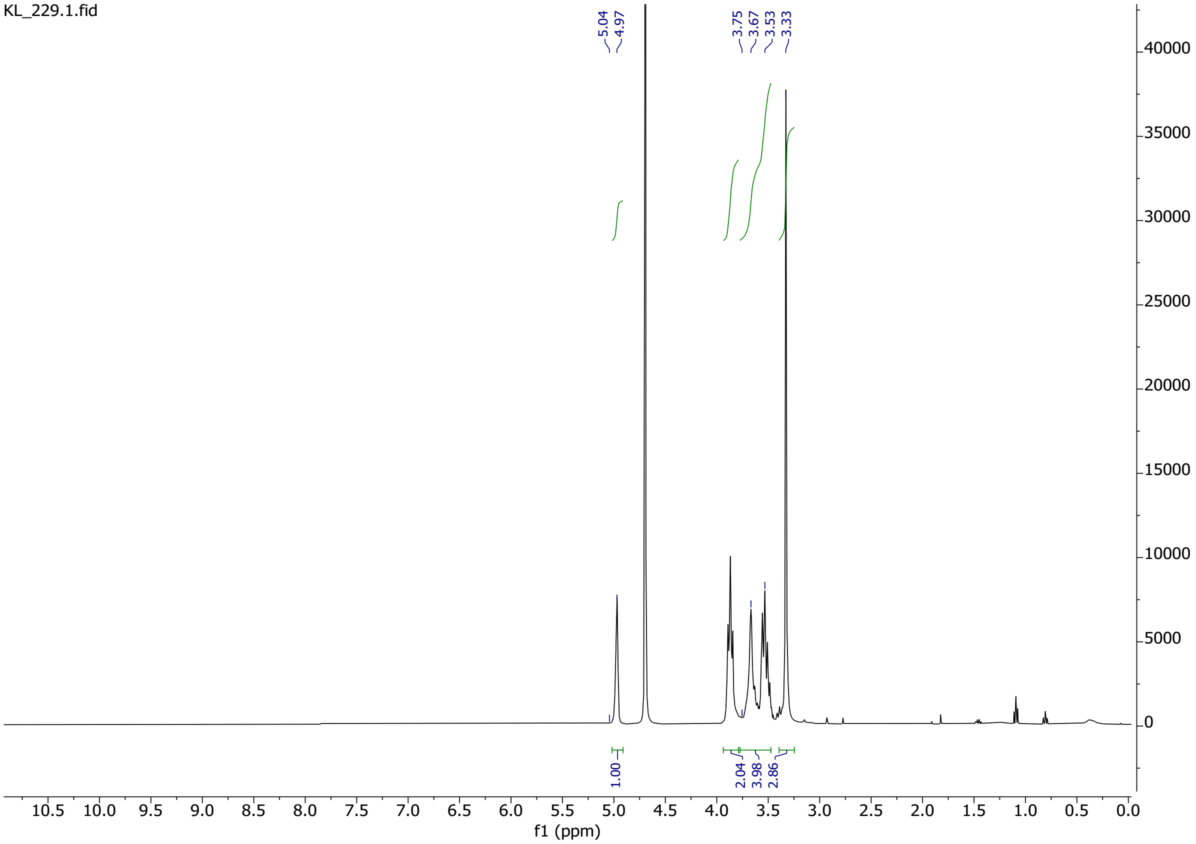

Fig. S69 DEPT-^{13}C-NMR spectrum of **12** in D_2O at 25 °C.

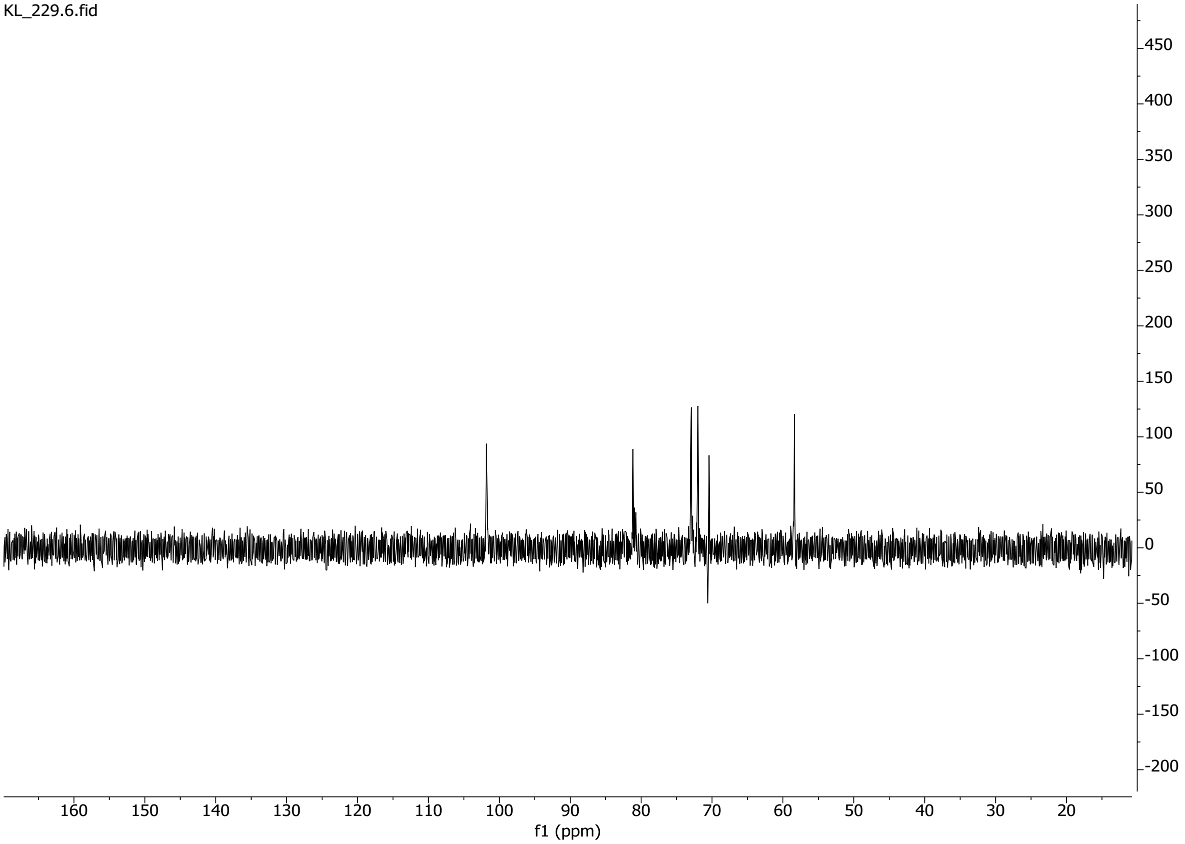

Fig. S70 COSY spectrum of **12** in D_2O at 25 °C.

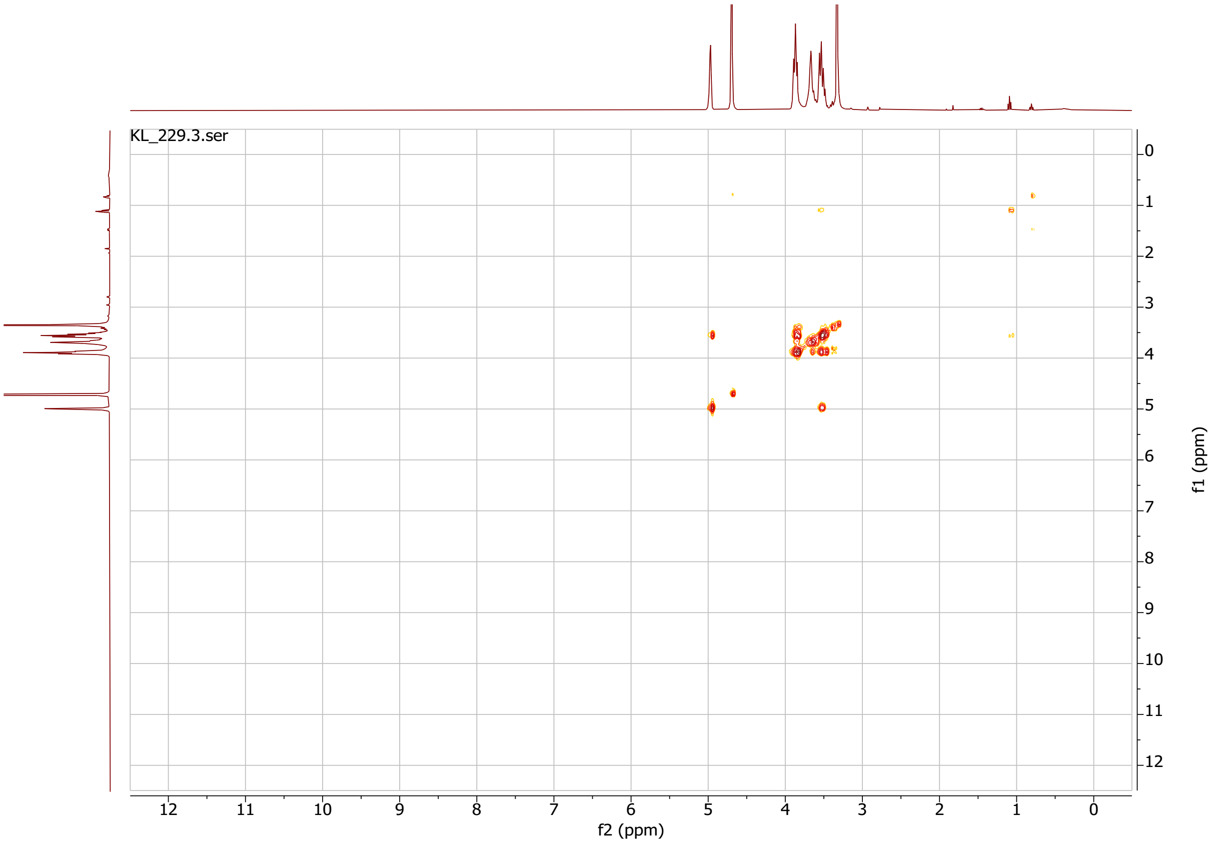

Fig. S71 HSQC spectrum of **12** in D_2O at 25 °C.

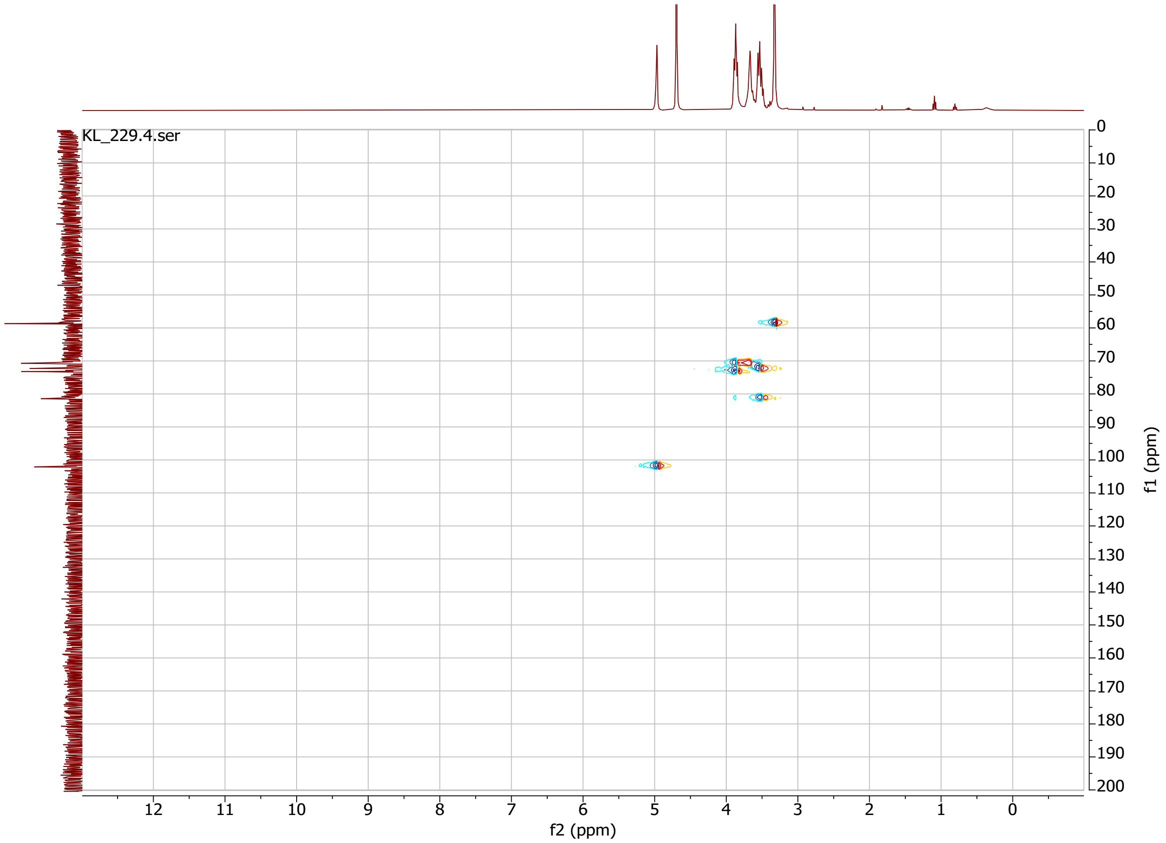

Fig. S72 IR spectrum of **12** in KBr pellet.

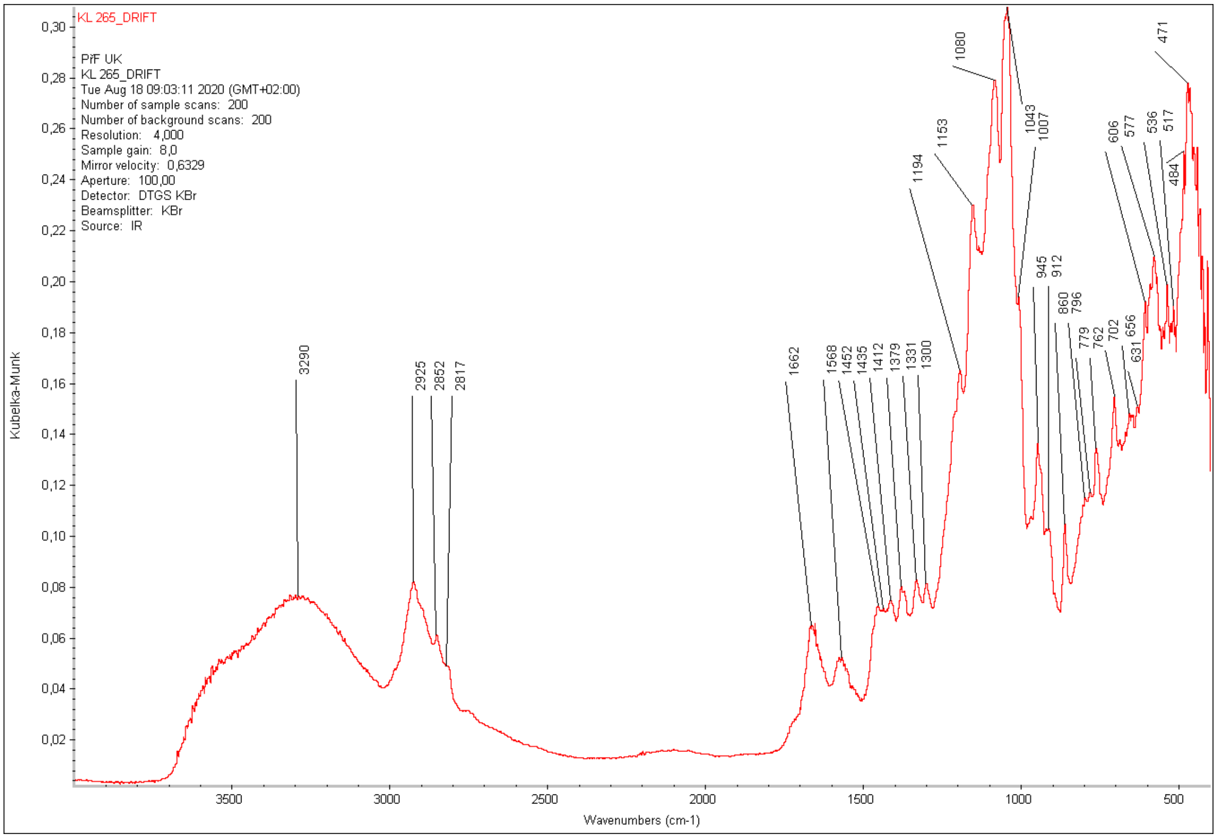

Fig. S73 UV–vis spectrum of **12** in DMSO.

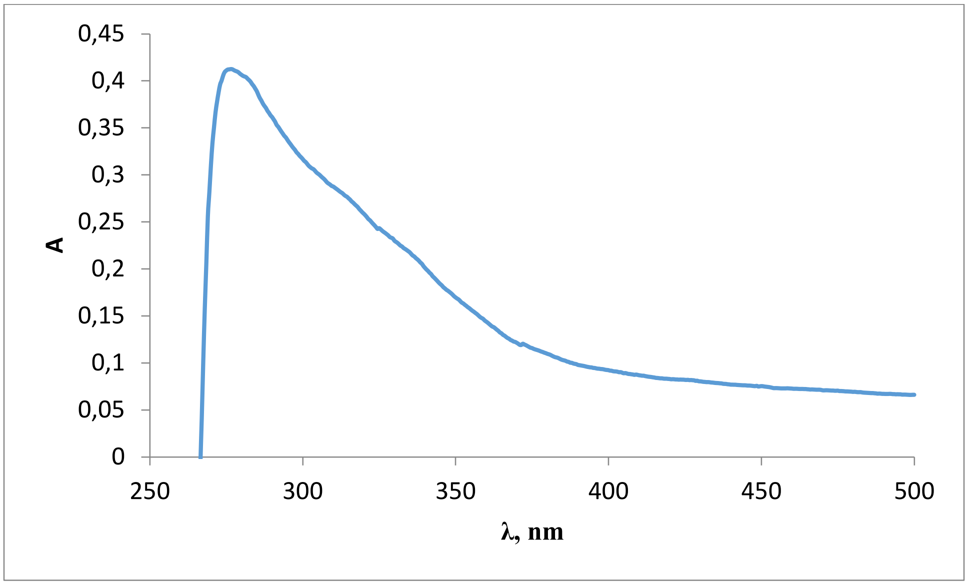

II UV experiments with tetracene

Fig. S74 UV–vis spectrum of tetracene in DMSO at different concentrations.

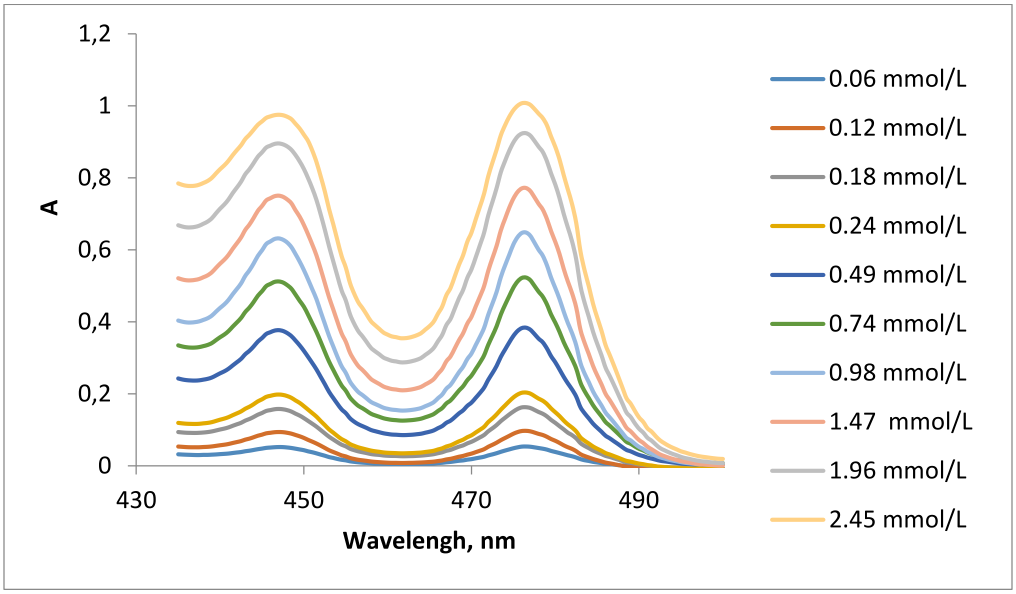

Fig. S75 Concentration dependence of tetracene's light absorbance in DMSO at 476 nm.

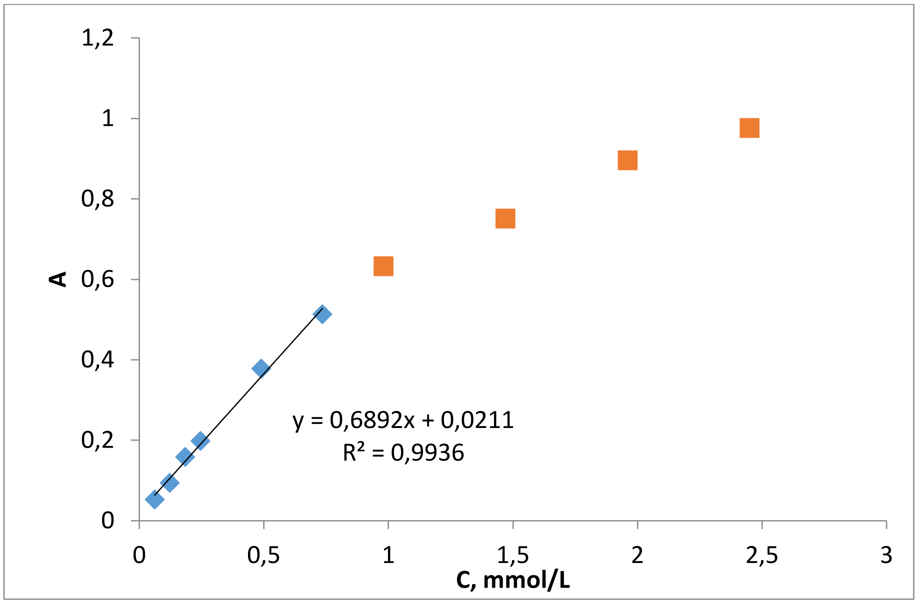

Fig. S76 UV–vis spectrum of 5-times diluted saturated tetracene solutions with the studied CDs and without any supramolecular host (O).

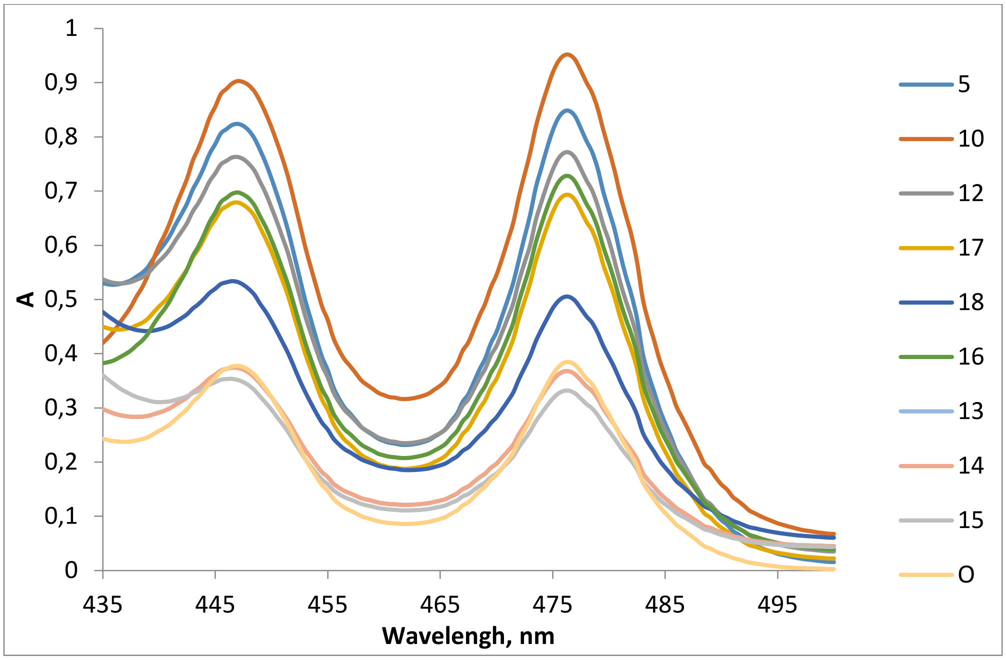

Fig. S77 UV–vis spectrum of 10-times diluted saturated tetracene solutions with the studied CDs and without any supramolecular host (O).

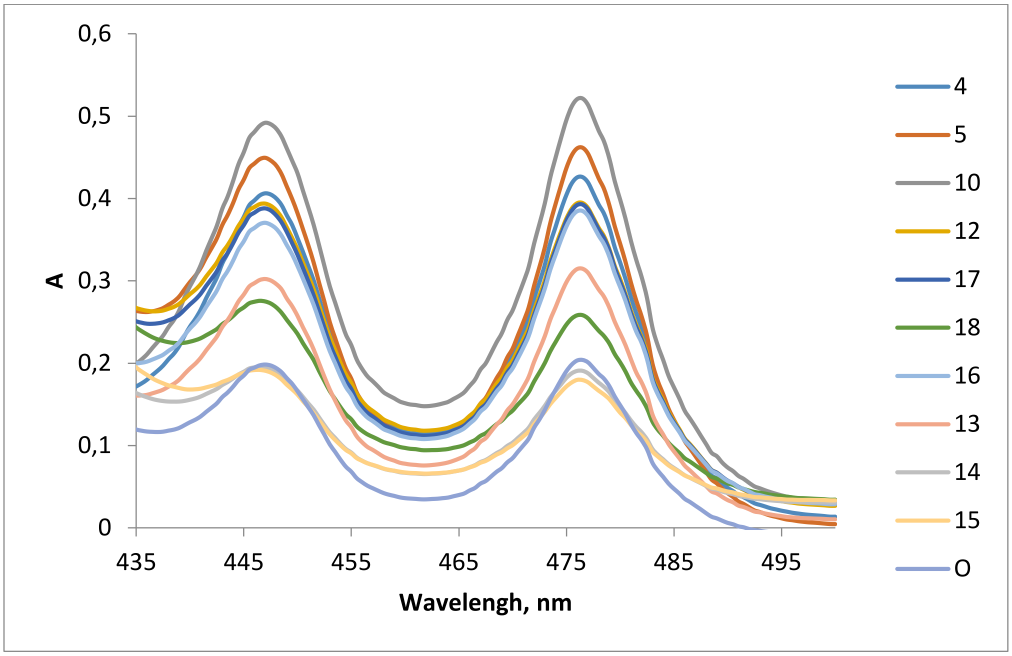

III ITC experiments

Fig. 78 ITC thermograms.

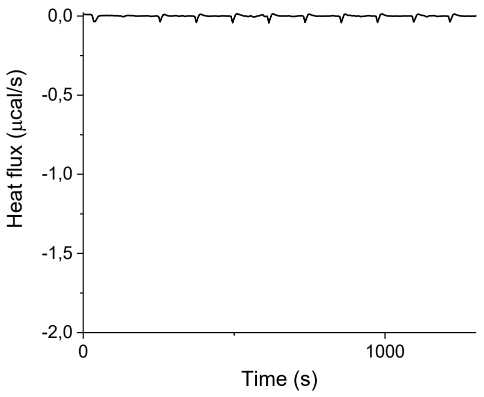

a) DMSO to DMSO

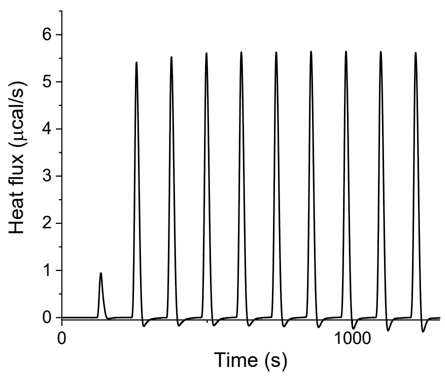

b) DMSO to DMSO with 5% vol. water

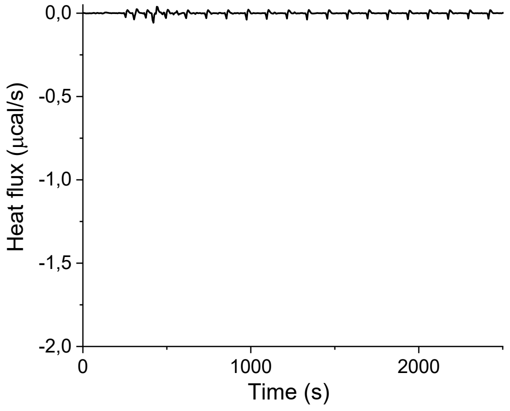

c) DMSO to tetracene in DMSO

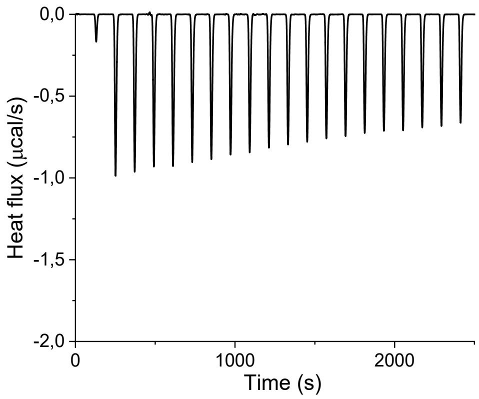 	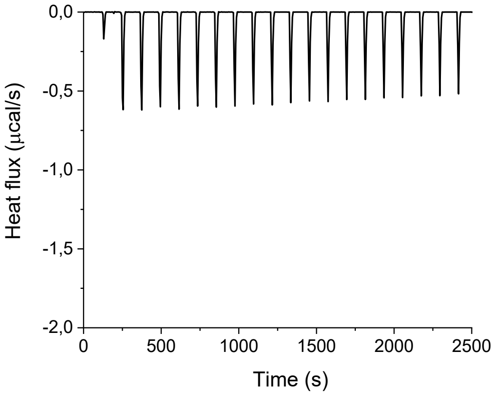
d) **13** dilution to DMSO	e) **13** to tetracene in DMSO
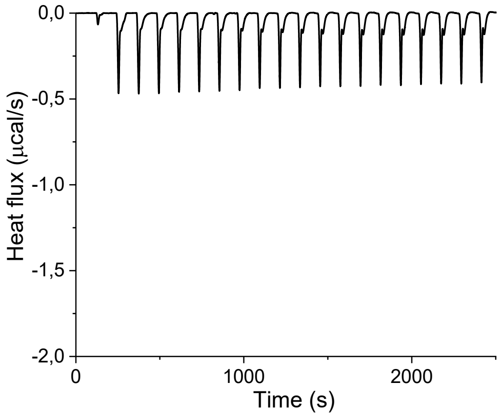 	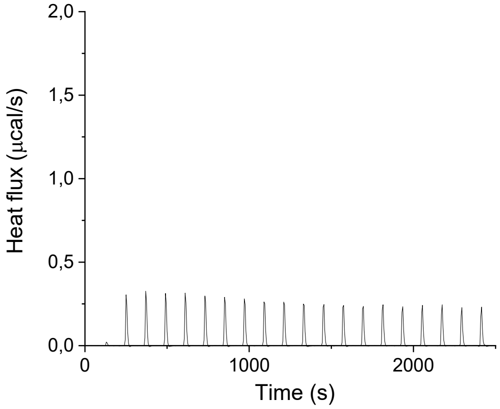
f) **16** to DMSO	g) **16** to tetracene in DMSO
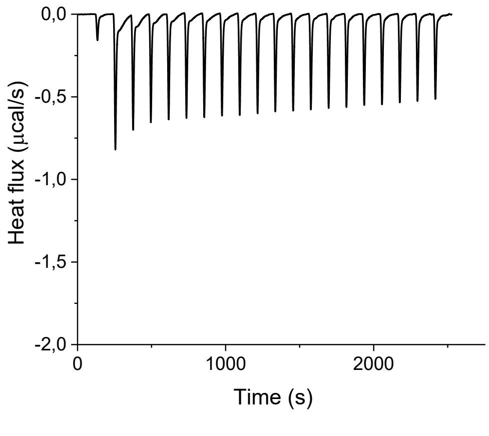 	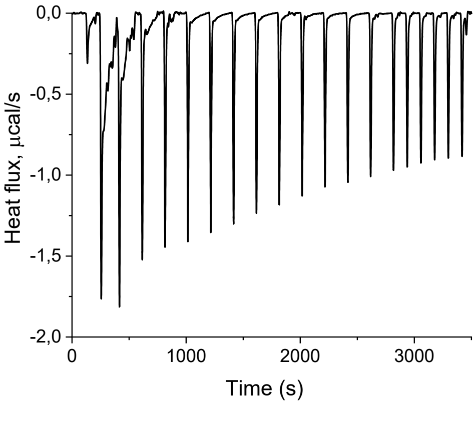

h) **4** to DMSO

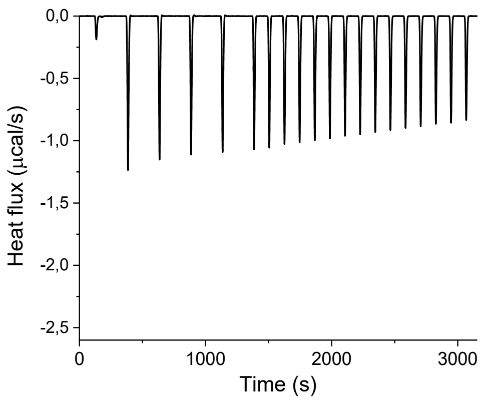

i) **4** to tetracene in DMSO

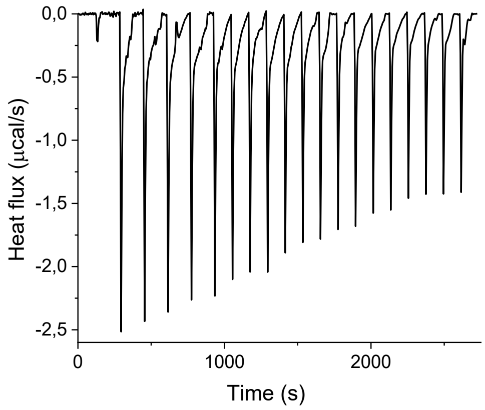

j) **5** to DMSO

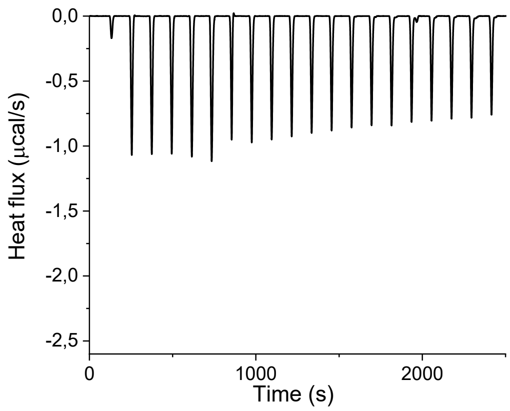

k) **5** to tetracene in DMSO

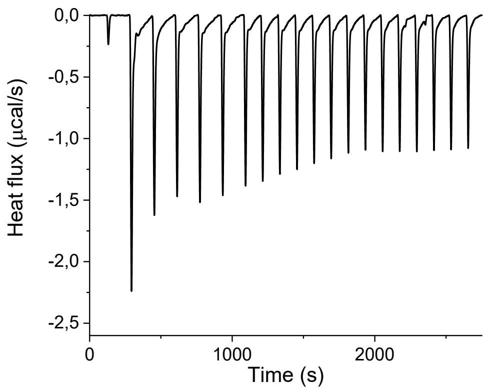

g) **10** to DMSO	h) **10** to tetracene in DMSO

IV References for SI

[1] *Org. Synth.*, **2000**, 77, 220.

[2] Tang, W.; Ng, S.-C. *Nat. Protoc.*, **2008**, 3, 691–697.

[3] Zhou, Y.; Lindbäck, E.; Marinescu, L. G.; Pedersen, C. M.; Bols, M. *Eur. J. Org. Chem.*, **2012**, 2012, 4063–4070.

[4] Varga, E.; Benkovics, G.; Darcsi, A.; Varnai, B.; Sohajda, T.; Malanga, M.; Beni, S. Electrophoresis, **2019**, 40, 2789–2798.

[5] Carofiglio, T.; Cordioli, M.; Fornasier, R.; Jicsinszky, L.; Tonellato, U. *Carbohydr. Res.*, **2004**, 339, No. 7, 1361–1366.
